# Supplementary material for: Identification of substrates of palmitoyl protein thioesterase 1 highlights roles of depalmitoylation in disulfide bond formation and synaptic function
Source: PLoS Biol. 2022 Mar 31;20(3):e3001590. doi: 10.1371/journal.pbio.3001590 (PMC9004782; doi:10.1371/journal.pbio.3001590)
Supplement: S1 Table — Average KO/WT protein expression ratios for data shown in Fig 2D. p-Values were calculated using a 2-tailed t test. Blue denotes decreases (<1.5 fold), red denotes increases (>1.5 fold), while black denotes unchanged expression. KO, knockout; PPT1, palmitoyl protein thioesterase 1; WT, wild-type. (PDF) [file pbio.3001590.s002.pdf]

S1 Table. WT and PPT1 KO synaptic protein expression. Average KO/WT protein expression ratios for data shown in **Figure 2D**. P-values were calculated using two-tailed t-tests. Blue denotes decreases (<1.5 fold), red denotes increases (>1.5 fold), while black denotes unchanged expression.

Uniprot ID	Average Ratio (KO/WT)	P value
PPT1_MOUSE	0.1324065	1.9306E-07
ASAH1_MOUSE	2.03207633	7.1104E-08
CATD_MOUSE	1.9598571	2.0852E-10
CX7A2_MOUSE	1.89377338	0.02926753
GFAP_MOUSE	1.58759041	0.00525781
NDUF4_MOUSE	1.55884846	0.0073044
NU4M_MOUSE	1.53679812	0.01790171
SAP_MOUSE	3.07387932	6.389E-11
SCRB2_MOUSE	1.87158589	0.00153495
TOM7_MOUSE	1.74901867	0.00948743
TPP1_MOUSE	2.73139373	7.4374E-08
1433B_MOUSE	0.98583501	0.5571686
1433E_MOUSE	0.98173666	0.47927337
1433F_MOUSE	0.92138057	0.09023641
1433G_MOUSE	0.94088453	0.13610869
1433T_MOUSE	0.9064798	0.05521134
1433Z_MOUSE	0.91939427	0.03163892
2A5B_MOUSE	0.91767961	0.17958149
2A5E_MOUSE	0.93588692	0.20594846
2A5G_MOUSE	0.92516276	0.27527372
2AAA_MOUSE	0.95194269	0.21285607
2ABA_MOUSE	0.95367966	0.22598709
3HIDH_MOUSE	1.04327957	0.79398386
4F2_MOUSE	1.02898308	0.66595994
68MP_MOUSE	1.2916347	0.1393345
6PGD_MOUSE	0.98481313	0.75742688
6PGL_MOUSE	0.99064213	0.75351609
A4_MOUSE	0.91521678	0.11759947
AACS_MOUSE	0.90033075	0.14738068
AAK1_MOUSE	0.98735689	0.64757685
AATC_MOUSE	0.99724389	0.88741276
AATM_MOUSE	1.01539692	0.81680797
ABCB7_MOUSE	1.03437721	0.72455275
ABCB8_MOUSE	0.92454716	0.04636432

ABCD3_MOUSE	1.05480501	0.51271416
ABD12_MOUSE	1.01144992	0.72044301
ABHDA_MOUSE	1.11382811	0.31998147
ABHGA_MOUSE	0.94834703	0.23558947
ABI1_MOUSE	1.00358768	0.84630625
ABI2_MOUSE	0.89302221	0.16879112
ABLM1_MOUSE	0.99187713	0.62553276
ABLM2_MOUSE	0.98401907	0.70335008
ABR_MOUSE	0.98729854	0.6186061
ACACA_MOUSE	0.94863088	0.29824119
ACAD8_MOUSE	0.95668948	0.37781067
ACAD9_MOUSE	0.9803481	0.49322535
ACADL_MOUSE	1.11932528	0.07927052
ACADM_MOUSE	1.04129721	0.52577499
ACADS_MOUSE	1.0463713	0.82805935
ACADV_MOUSE	1.03872925	0.38873496
ACBD6_MOUSE	0.89777266	0.10916865
ACBG1_MOUSE	0.9736729	0.50146583
ACBP_MOUSE	0.94736514	0.34919227
ACDSB_MOUSE	1.02078461	0.76794946
ACES_MOUSE	0.96364846	0.54106011
ACLY_MOUSE	0.98858338	0.64280925
ACO11_MOUSE	0.97019004	0.46162536
ACO13_MOUSE	1.07276753	0.30443162
ACOC_MOUSE	1.02463082	0.62056124
ACON_MOUSE	1.02583766	0.81032263
ACOT9_MOUSE	1.05205746	0.23715939
ACOX1_MOUSE	1.35749642	0.01158117
ACPM_MOUSE	1.15626307	0.05123628
ACSF2_MOUSE	1.19753129	0.02615009
ACSF3_MOUSE	1.02599796	0.83642539
ACSL1_MOUSE	1.03678754	0.49813489
ACSL3_MOUSE	1.00424691	0.83509572
ACSL6_MOUSE	1.04949839	0.22151683
ACTB_MOUSE	0.92146447	0.09707842
ACTC_MOUSE	1.08163565	0.54517998
ACTG_MOUSE	0.94785731	0.3123261
ACTN1_MOUSE	1.04246345	0.58964527
ACTN2_MOUSE	1.00436901	0.9617644

ACTN4_MOUSE	0.89503777	0.0658385
ACTY_MOUSE	1.01662349	0.64068865
ACTZ_MOUSE	1.00274656	0.90438894
ACY2_MOUSE	0.75121584	0.00017773
ACYP1_MOUSE	0.95202532	0.200298
ADA11_MOUSE	1.01879609	0.85015647
ADA22_MOUSE	1.04358024	0.76282963
ADA23_MOUSE	0.99141222	0.6482934
ADCK1_MOUSE	0.99950778	0.98478884
ADCY5_MOUSE	0.93989396	0.1990365
ADCY9_MOUSE	0.97061256	0.48192215
ADDA_MOUSE	1.00947852	0.86514912
ADDB_MOUSE	1.04320106	0.21928638
ADDG_MOUSE	0.98292048	0.55179324
ADHX_MOUSE	0.95361516	0.0875614
ADK_MOUSE	1.02157436	0.87405279
ADPRH_MOUSE	1.03361479	0.68142929
ADT1_MOUSE	1.00478358	0.92765781
ADT2_MOUSE	0.99952531	0.75706883
AFAD_MOUSE	1.0307234	0.78500273
AFG32_MOUSE	0.99103875	0.6276594
AGAP2_MOUSE	0.98011748	0.47876885
AGAP3_MOUSE	0.99019542	0.68336469
AGFG1_MOUSE	1.00131728	0.92463358
AGK_MOUSE	1.08320684	0.62013365
AGM1_MOUSE	0.88484876	0.0086762
AGRL1_MOUSE	1.01473453	0.78894441
AGRL3_MOUSE	0.94233571	0.25288306
AHSA1_MOUSE	1.03581732	0.67341969
AIFM1_MOUSE	0.97016079	0.40862561
AINX_MOUSE	1.15678731	0.12331887
AK1A1_MOUSE	0.98262512	0.52720552
AKA12_MOUSE	0.88688352	0.23433627
AKA7A_MOUSE	1.01473012	0.82872316
AKAP5_MOUSE	1.01304215	0.95406776
AKCL2_MOUSE	1.01667196	0.94332204
AL1A1_MOUSE	1.01979462	0.79180123
AL1B1_MOUSE	0.99870187	0.77295367
AL1L1_MOUSE	1.033779	0.55294913

AL3A2_MOUSE	0.96422921	0.43583518
AL4A1_MOUSE	1.07133763	0.26487501
AL7A1_MOUSE	1.11255825	0.11033686
AL9A1_MOUSE	1.04619003	0.33937178
ALBU_MOUSE	0.73056189	0.00300301
ALDH2_MOUSE	1.01015857	0.8397427
ALDOA_MOUSE	1.01221097	0.9584375
ALDOC_MOUSE	1.05019299	0.51227259
ALDR_MOUSE	0.97163715	0.36689111
ALG2_MOUSE	0.94537116	0.35234149
ALR_MOUSE	0.9387795	0.46413448
AMD_MOUSE	1.09569923	0.22764743
AMPB_MOUSE	0.9187945	0.13921191
AMPD2_MOUSE	1.04600762	0.52170757
AMPH_MOUSE	0.8869798	0.07170537
AMPL_MOUSE	1.08568661	0.23477562
AMRP_MOUSE	0.95903601	0.41822429
AN32A_MOUSE	0.86474585	0.03681287
ANK1_MOUSE	1.04075792	0.81146394
ANK2_MOUSE	1.00200971	0.84572697
ANK3_MOUSE	1.01682377	0.85371332
ANM5_MOUSE	0.90454203	0.07330723
ANS1B_MOUSE	0.9868218	0.55807941
ANXA3_MOUSE	0.96377447	0.57577386
ANXA5_MOUSE	1.06104112	0.0196424
ANXA6_MOUSE	1.02629433	0.33126602
ANXA7_MOUSE	0.90795603	0.21982937
AOFA_MOUSE	1.03214509	0.27253897
AOFB_MOUSE	1.13364749	0.10433588
AP180_MOUSE	1.01811375	0.86287515
AP1B1_MOUSE	1.0048851	0.85289081
AP1G1_MOUSE	1.02365273	0.58922965
AP1M1_MOUSE	1.00142115	0.91196347
AP2A1_MOUSE	0.9960234	0.77657554
AP2A2_MOUSE	1.00618472	0.9535714
AP2B1_MOUSE	0.99987012	0.83752095
AP2M1_MOUSE	0.98002538	0.58189395
AP2S1_MOUSE	0.95923402	0.36236442
AP3B2_MOUSE	0.97649273	0.38641444

AP3D1_MOUSE	1.03504899	0.44215605
AP3M2_MOUSE	0.92518115	0.25674898
APEH_MOUSE	1.02170056	0.86370785
APMAP_MOUSE	1.15227943	0.07341667
APOE_MOUSE	1.0812543	0.56552118
AQP4_MOUSE	1.28825636	0.00808742
ARBK1_MOUSE	1.0158497	0.9584319
ARC1A_MOUSE	1.0531272	0.6923467
ARF1_MOUSE	1.0347915	0.83563863
ARF5_MOUSE	1.06912599	0.44875724
ARF6_MOUSE	1.14332595	0.18694691
ARFG1_MOUSE	0.96255979	0.33486025
ARHG2_MOUSE	0.99281239	0.67501943
ARHG7_MOUSE	1.27181402	0.418026
ARK72_MOUSE	1.08551521	0.16493593
ARL3_MOUSE	1.04119279	0.60250279
ARL6_MOUSE	0.95776521	0.33337084
ARL8A_MOUSE	1.37307239	0.20876189
ARL8B_MOUSE	1.18856673	2.5796E-07
ARLY_MOUSE	0.99671467	0.86622609
ARM10_MOUSE	0.97750237	0.47536565
ARMC1_MOUSE	1.10962526	0.34182471
ARP10_MOUSE	0.98782761	0.70727122
ARP2_MOUSE	1.00865463	0.8602501
ARP3_MOUSE	1.02234308	0.85924876
ARP3B_MOUSE	0.97602424	0.38563952
ARP5L_MOUSE	0.97062534	0.32796031
ARPC2_MOUSE	1.05881648	0.64536015
ARPC3_MOUSE	0.98864959	0.60010269
ARPC4_MOUSE	1.00251771	0.97396826
ARPC5_MOUSE	1.00454984	0.88192802
ARRB1_MOUSE	1.17781089	0.09450326
ASAP1_MOUSE	1.02446025	0.44293523
ASGL1_MOUSE	0.98519099	0.50916938
ASNA_MOUSE	1.04192616	0.38204748
ASSY_MOUSE	1.11586184	0.02223865
ASTN1_MOUSE	0.9876944	0.77449576
AT1A1_MOUSE	0.9595546	0.31795481
AT1A2_MOUSE	1.06757864	0.4977135

AT1A3_MOUSE	1.04236915	0.69483968
AT1A4_MOUSE	1.04424697	0.91097065
AT1B1_MOUSE	0.98238956	0.53580402
AT1B2_MOUSE	1.04328394	0.47105468
AT1B3_MOUSE	0.99751792	0.84914292
AT2A2_MOUSE	1.0596817	0.16985843
AT2B1_MOUSE	1.0270506	0.86560869
AT2B2_MOUSE	1.01730849	0.99801045
AT2B4_MOUSE	0.99535077	0.73609152
AT5F1_MOUSE	1.00319234	0.87444768
AT8A1_MOUSE	0.96619097	0.34216116
ATAD1_MOUSE	1.04746733	0.45438604
ATAD3_MOUSE	0.98255806	0.45504613
ATCAY_MOUSE	1.05933576	0.39736066
ATIF1_MOUSE	1.05066462	0.6879365
ATLA1_MOUSE	0.97194914	0.4438328
ATOX1_MOUSE	0.93376963	0.15060377
ATP5E_MOUSE	1.00809842	0.99806239
ATP5H_MOUSE	0.92005157	0.09611631
ATP5I_MOUSE	0.97613267	0.42718863
ATP5L_MOUSE	1.09959222	0.18266955
ATP5S_MOUSE	1.03155476	0.8118487
ATPA_MOUSE	0.99393389	0.72710112
ATPB_MOUSE	1.0172077	0.86278579
ATPD_MOUSE	0.99637597	0.93358862
ATPG_MOUSE	1.00122588	0.77843614
ATPK_MOUSE	0.98789021	0.60452132
ATPO_MOUSE	1.05952768	0.27717713
ATX10_MOUSE	0.98139324	0.57779581
AUHM_MOUSE	0.97972141	0.62492621
AUXI_MOUSE	0.98502072	0.58811037
AVL9_MOUSE	0.93631189	0.12373438
B2L13_MOUSE	1.06604817	0.93140479
BACH_MOUSE	0.99629742	0.82209133
BAG5_MOUSE	0.89824369	0.17397646
BAG6_MOUSE	0.92829455	0.32757858
BAIP2_MOUSE	0.91716911	0.05178492
BAP31_MOUSE	1.09602585	0.48593776
BASI_MOUSE	0.92651477	0.10129023

BASP1_MOUSE	0.8743612	0.19000108
BCAS1_MOUSE	0.90566058	0.05902413
BCAS3_MOUSE	0.96906907	0.34555007
BCAT1_MOUSE	1.07346554	0.19801492
BCS1_MOUSE	1.13379284	0.20483242
BDH_MOUSE	0.96620927	0.33887951
BGAL_MOUSE	0.93461801	0.2449892
BIG3_MOUSE	0.89871907	0.08650094
BIN1_MOUSE	0.90107359	0.04818179
BLMH_MOUSE	0.92873827	0.10889336
BOLA1_MOUSE	0.94794213	0.50879243
BORG4_MOUSE	0.93035347	0.18178535
BPHL_MOUSE	1.04955425	0.60709743
BPNT1_MOUSE	0.99460767	0.69089273
BRNP1_MOUSE	1.29594192	0.08909956
BRSK2_MOUSE	1.0463491	0.855304
BSN_MOUSE	1.00353359	0.83568539
BTBDH_MOUSE	1.212659	0.46128881
C170B_MOUSE	1.15183827	0.12726058
C1QBP_MOUSE	1.0023987	0.92550211
C1TC_MOUSE	1.00011439	0.72533191
C1TM_MOUSE	0.98036808	0.48750339
C2C2L_MOUSE	1.0179609	0.8488582
C560_MOUSE	1.25260395	0.03019851
CA198_MOUSE	1.15048926	0.74403633
CA2D1_MOUSE	0.95154417	0.26865256
CA2D2_MOUSE	1.02906302	0.94937902
CA2D3_MOUSE	1.01430167	0.95635947
CAB39_MOUSE	0.9523452	0.10933287
CAC1A_MOUSE	0.98578988	0.63522475
CAC1B_MOUSE	1.04865125	0.32909221
CAC1E_MOUSE	0.92656924	0.07838562
CACB4_MOUSE	0.96890641	0.45065068
CACP_MOUSE	1.04455971	0.36116959
CAD10_MOUSE	0.95799348	0.45151004
CAD13_MOUSE	1.08840294	0.29656473
CADH2_MOUSE	0.92883115	0.14238845
CADM1_MOUSE	0.99888376	0.92261315
CADM2_MOUSE	0.98822666	0.55928359

CADM3_MOUSE	0.96229727	0.23162547
CADM4_MOUSE	0.99921341	0.74677347
CAH2_MOUSE	0.9190263	0.15752963
CAH4_MOUSE	0.94563712	0.33906131
CALB1_MOUSE	0.94978381	0.12675795
CALB2_MOUSE	0.84185513	0.00569191
CALR_MOUSE	1.1544788	0.0004207
CALU_MOUSE	0.91843886	0.28027337
CALX_MOUSE	0.9622982	0.36377171
CAMKV_MOUSE	1.00538188	0.90275622
CAN2_MOUSE	1.2112102	0.67318094
CAN5_MOUSE	0.96056614	0.36313268
CANB1_MOUSE	0.86871042	0.01601081
CAND1_MOUSE	0.95910474	0.16247384
CAP1_MOUSE	0.89514036	0.06809104
CAP2_MOUSE	0.98840445	0.7293977
CAPS1_MOUSE	0.98369221	0.58325628
CAPS2_MOUSE	0.98320306	0.67393579
CAPZB_MOUSE	1.00533049	0.73747021
CATA_MOUSE	1.04312494	0.64012675
CATB_MOUSE	0.93026251	0.25469791
CAZA2_MOUSE	0.97450427	0.24086604
CBPE_MOUSE	1.05094231	0.23541524
CBR1_MOUSE	0.97982834	0.58748925
CBR3_MOUSE	0.93294688	0.28053853
CBR4_MOUSE	1.0835083	0.09933198
CC127_MOUSE	1.5830901	0.44604952
CC177_MOUSE	0.90653937	0.17033253
CC50A_MOUSE	1.01095507	0.83571496
CCD22_MOUSE	0.82714551	0.03377642
CCD51_MOUSE	1.14015917	0.36030134
CCD58_MOUSE	0.84892381	0.01618713
CCG8_MOUSE	0.99124283	0.63643284
CCHL_MOUSE	1.01307138	0.96567431
CCNY_MOUSE	0.92286009	0.13024897
CD166_MOUSE	1.05170851	0.60211882
CD47_MOUSE	0.96876125	0.36990625
CD81_MOUSE	1.03479067	0.93953981
CDC37_MOUSE	1.00944186	0.87190281

CDC42_MOUSE	1.16481882	0.07457434
CDK14_MOUSE	1.03842199	0.66498252
CDK5_MOUSE	0.94135374	0.18021069
CDS2_MOUSE	1.06361652	0.66715341
CDV3_MOUSE	0.84562167	0.00777649
CE170_MOUSE	0.95359754	0.19655359
CH10_MOUSE	1.01405085	0.7927479
CH60_MOUSE	1.05026896	0.44901962
CHM4B_MOUSE	0.88615465	0.07725902
CHP1_MOUSE	0.97361276	0.59807413
CHRD1_MOUSE	1.11581416	0.44098761
CI172_MOUSE	0.86262242	0.07564175
CISD1_MOUSE	0.99149775	0.63514151
CISY_MOUSE	1.01336742	0.85909404
CK054_MOUSE	0.88981071	0.03886698
CKAP5_MOUSE	0.93671912	0.00571075
CLAP1_MOUSE	1.01745178	0.89076905
CLAP2_MOUSE	1.02172874	0.88773472
CLCA_MOUSE	0.84338981	0.013501
CLCB_MOUSE	0.82982504	0.00669776
CLCN3_MOUSE	1.29392742	0.22838283
CLD11_MOUSE	0.81911986	0.01197204
CLH1_MOUSE	0.99784733	0.80263571
CLIC4_MOUSE	0.93548445	0.17646921
CLIP2_MOUSE	0.94767997	0.33256559
CLPB_MOUSE	1.00440141	0.887557
CLPP_MOUSE	1.07192347	0.51639365
CLUS_MOUSE	1.08039679	0.37687686
CLYBL_MOUSE	1.07281643	0.04874069
CMC1_MOUSE	1.03860837	0.59507996
CMPK2_MOUSE	1.08749447	0.32077969
CMTD1_MOUSE	1.34726272	0.07243413
CN166_MOUSE	0.91843793	0.19818151
CN37_MOUSE	0.92125707	0.39967553
CNDP2_MOUSE	0.9815315	0.54505329
CNKR2_MOUSE	0.920793	0.01017472
CNRP1_MOUSE	1.03108139	0.83873952
CNTFR_MOUSE	1.37733168	0.01384741
CNTN1_MOUSE	0.98686495	0.60633784

CNTN2_MOUSE	1.03331635	0.70007104
CNTP1_MOUSE	0.97170666	0.46900266
CNTP2_MOUSE	1.01686306	0.89940382
COA3_MOUSE	0.98716024	0.54723272
COASY_MOUSE	1.15434533	0.03977472
COF1_MOUSE	0.91900253	0.03557167
COF2_MOUSE	0.94253162	0.09981511
COQ3_MOUSE	0.90916542	0.05388269
COQ7_MOUSE	1.04636963	0.48710164
COQ9_MOUSE	0.96062365	0.15531676
COR1A_MOUSE	0.9190303	0.16189404
COR1B_MOUSE	1.04250741	0.81872739
COR1C_MOUSE	0.99253369	0.70772359
COR2B_MOUSE	1.03678945	0.42309674
COTL1_MOUSE	1.02943345	0.07583358
COX1_MOUSE	1.17569611	0.04054381
COX2_MOUSE	1.0305403	0.50737917
COX41_MOUSE	0.97846337	0.46640039
COX5A_MOUSE	0.84849241	0.00315668
COX5B_MOUSE	0.94679574	0.15242048
COX6C_MOUSE	1.08279404	0.37027397
CP46A_MOUSE	1.04310061	0.53548127
CPLX1_MOUSE	0.81098979	0.00115705
CPLX2_MOUSE	0.91795691	0.08469109
CPNE4_MOUSE	0.99782195	0.84431327
CPNE5_MOUSE	0.98856654	0.58965193
CPNE6_MOUSE	1.02713074	0.89167052
CPNS1_MOUSE	0.98284553	0.57231254
CPT1A_MOUSE	1.06855799	0.19259065
CPT2_MOUSE	1.11046303	0.07564793
CRIP2_MOUSE	0.94383489	0.19591961
CRK_MOUSE	0.93457824	0.25668922
CRKL_MOUSE	0.94071745	0.25477558
CRYAB_MOUSE	0.97876345	0.63069604
CRYM_MOUSE	1.05918965	0.51321635
CSDE1_MOUSE	0.99040543	0.57757061
CSK21_MOUSE	0.99106267	0.71192639
CSK22_MOUSE	1.08951809	0.45089363
CSK2B_MOUSE	0.95229064	0.4786563

CSKI1_MOUSE	0.98333622	0.58862239
CSKP_MOUSE	1.07619916	0.00986741
CSN1_MOUSE	1.05542673	0.16096471
CSN2_MOUSE	0.95422923	0.23502523
CSN3_MOUSE	0.98074366	0.56895981
CSN4_MOUSE	0.88975204	0.06950214
CSN5_MOUSE	0.98075794	0.52192456
CSN6_MOUSE	0.93798092	0.0997927
CSN7A_MOUSE	1.13906556	0.18974143
CSN8_MOUSE	0.92506182	0.14692901
CSPG2_MOUSE	0.99899671	0.78582444
CSPG5_MOUSE	0.99269714	0.83121483
CSRP1_MOUSE	1.01129448	0.97490544
CTBP1_MOUSE	1.00547639	0.95769061
CTL1_MOUSE	1.03967704	0.82031314
CTL2_MOUSE	1.14361577	0.00165209
CTNA2_MOUSE	1.01094537	0.88083354
CTNB1_MOUSE	1.05338736	0.5272842
CTND1_MOUSE	1.02841053	0.51018581
CTND2_MOUSE	1.02757612	0.98433427
CTRO_MOUSE	1.03887563	0.39144788
CTTB2_MOUSE	1.02500665	0.88456958
CUL1_MOUSE	1.11502018	0.33760585
CUL2_MOUSE	1.09130302	0.05861195
CUL3_MOUSE	0.96472128	0.30651475
CUL5_MOUSE	0.98430693	0.57982141
CX6B1_MOUSE	0.9192662	0.22444627
CXA1_MOUSE	1.2141345	0.00626056
CY1_MOUSE	1.01025959	0.99007648
CYB5_MOUSE	0.96179705	0.34467179
CYB5B_MOUSE	1.15539448	0.4025798
CYBP_MOUSE	1.07062605	0.33654221
CYC_MOUSE	0.89801836	0.28862416
CYFP1_MOUSE	1.05671175	0.369241
CYFP2_MOUSE	1.01266061	0.9889635
CYTB_MOUSE	1.17074555	0.08209781
CYTC_MOUSE	1.04549651	0.70773885
CYTSB_MOUSE	0.9534108	0.32053881
D39U1_MOUSE	1.07272007	0.63741409

DAAM1_MOUSE	0.99471311	0.59917324
DBNL_MOUSE	0.90593185	0.07662524
DC1I1_MOUSE	1.06843691	0.42622884
DC1I2_MOUSE	0.98030454	0.60169112
DC1L1_MOUSE	0.95942033	0.15816691
DC1L2_MOUSE	0.94577638	0.18708307
DCE1_MOUSE	1.01945279	0.89450169
DCE2_MOUSE	1.05164401	0.24559098
DCLK1_MOUSE	1.02631587	0.72575584
DCNL1_MOUSE	0.93018092	0.45316203
DCTN1_MOUSE	0.97410227	0.3428325
DCTN2_MOUSE	0.8723489	0.04879818
DCTN3_MOUSE	0.96010336	0.32108891
DCTN4_MOUSE	0.9120714	0.02269415
DDAH1_MOUSE	1.00657171	0.79241971
DDAH2_MOUSE	0.89077874	0.01788754
DDB1_MOUSE	1.03718516	0.31974592
DDC_MOUSE	0.94106925	0.25370814
DDX1_MOUSE	1.02867098	0.73475988
DDX3L_MOUSE	0.96044118	0.35035191
DDX6_MOUSE	0.84713051	0.02090482
DECR_MOUSE	1.12356258	0.05415661
DEMA_MOUSE	0.99371961	0.82245602
DEST_MOUSE	0.96836695	0.46617128
DGKB_MOUSE	1.03468591	0.71279534
DHB11_MOUSE	1.26549881	0.43127931
DHB12_MOUSE	1.10137074	0.13365641
DHB4_MOUSE	1.04702299	0.34573986
DHB8_MOUSE	1.00397809	0.88354124
DHE3_MOUSE	1.08900467	0.18567775
DHPR_MOUSE	1.02703101	0.97977501
DHRS1_MOUSE	1.26654408	0.00214101
DHRS4_MOUSE	1.12242795	0.02608375
DHSO_MOUSE	0.91720589	0.18594359
DIC_MOUSE	1.04545093	0.49344677
DIP2A_MOUSE	0.99926077	0.68799539
DIP2B_MOUSE	1.00294245	0.8818966
DIRA2_MOUSE	0.98643459	0.70449186
DJC11_MOUSE	1.03453362	0.61718016

DLDH_MOUSE	0.99575126	0.78047943
DLG1_MOUSE	0.9963699	0.68242344
DLG2_MOUSE	0.95438399	0.34617645
DLG3_MOUSE	0.99918288	0.6537367
DLG4_MOUSE	0.97712365	0.51314291
DLGP1_MOUSE	0.87991575	0.01500004
DLGP2_MOUSE	0.96138788	0.21621455
DLGP3_MOUSE	0.96268039	0.23103275
DLGP4_MOUSE	0.94903038	0.07619281
DLRB1_MOUSE	0.94692853	0.34518253
DMXL2_MOUSE	0.9836664	0.55030164
DNJA1_MOUSE	1.0849886	0.23511034
DNJA2_MOUSE	1.01726057	0.99256824
DNJA3_MOUSE	0.98547798	0.58687236
DNJC5_MOUSE	0.8396206	0.01771918
DNM1L_MOUSE	0.99609404	0.75402022
DNPEP_MOUSE	0.98793225	0.68215435
DOCK3_MOUSE	1.82859663	0.46020798
DOPD_MOUSE	0.99637672	0.89844903
DP13A_MOUSE	0.97109727	0.40096222
DPP10_MOUSE	1.00518735	0.92117178
DPP3_MOUSE	0.82733183	0.01200881
DPP6_MOUSE	0.99761789	0.77206678
DPYL1_MOUSE	1.05114335	0.66023886
DPYL2_MOUSE	0.96468052	0.45238635
DPYL3_MOUSE	0.92718025	0.09345604
DPYL4_MOUSE	0.93956619	0.16580306
DPYL5_MOUSE	1.02932723	0.43527973
DREB_MOUSE	0.98338056	0.6081713
DRG2_MOUSE	1.05655813	0.7342031
DRS7B_MOUSE	0.91558019	0.05046646
DTNA_MOUSE	1.10998409	0.67179201
DUS3_MOUSE	0.94153834	0.2262102
DYHC1_MOUSE	0.99996041	0.84369198
DYL2_MOUSE	0.99610748	0.80791181
DYN1_MOUSE	0.97528094	0.4249306
DYN2_MOUSE	0.88939552	0.04723384
DYN3_MOUSE	1.0115484	0.9810353
DYST_MOUSE	1.00624913	0.65550488

E41L1_MOUSE	0.99050655	0.63297553
E41L2_MOUSE	1.04101442	0.40409144
E41L3_MOUSE	1.04682247	0.27021034
EAA1_MOUSE	1.13709342	0.05145232
EAA2_MOUSE	1.11686724	0.16111993
ECH1_MOUSE	1.07770154	0.27514606
ECHA_MOUSE	1.0736688	0.11715792
ECHB_MOUSE	1.05260365	0.22163638
ECHM_MOUSE	0.99901761	0.78677796
ECI1_MOUSE	1.11886048	0.08396099
ECI2_MOUSE	1.0349254	0.93652359
EEA1_MOUSE	0.98202061	0.74602379
EF1A1_MOUSE	1.04052376	0.65534008
EF1A2_MOUSE	1.24083043	0.14898333
EF1B_MOUSE	0.92366787	0.15514706
EF1D_MOUSE	0.95404727	0.32038185
EF1G_MOUSE	1.01892642	0.997511
EF2_MOUSE	1.01448902	0.9026785
EFGM_MOUSE	0.93074539	0.14217418
EFHD2_MOUSE	0.95021554	0.16893521
EFR3B_MOUSE	1.03110436	0.28523835
EFTS_MOUSE	1.0346267	0.40961755
EFTU_MOUSE	0.99893827	0.79130208
EHD1_MOUSE	0.94490567	0.07504018
EHD3_MOUSE	0.97369621	0.35187298
EHD4_MOUSE	1.00516853	0.99944337
EI3JA_MOUSE	0.8001277	0.05996764
EIF3A_MOUSE	0.99040482	0.60801567
ELMO2_MOUSE	0.89811497	0.01129495
ELOB_MOUSE	1.14898073	0.17528143
ELOC_MOUSE	1.26510449	0.22061637
EMC1_MOUSE	1.13960032	0.12573863
ENAH_MOUSE	0.93990372	0.47147706
ENDD1_MOUSE	0.9772786	0.47484263
ENOA_MOUSE	1.00683064	0.84549813
ENOG_MOUSE	0.91257124	0.13439704
ENPL_MOUSE	1.0451462	0.32731152
ENPP6_MOUSE	0.77429732	0.00174224
ENSA_MOUSE	0.93155012	0.23966422

ENTP2_MOUSE	1.23727555	0.10957402
EP15R_MOUSE	0.93191318	0.13536493
EPHA4_MOUSE	0.93087299	0.18969246
EPMIP_MOUSE	1.34136797	0.03202506
EPN1_MOUSE	0.89128225	0.02558887
ERC2_MOUSE	0.91653563	0.05824564
ERF1_MOUSE	0.89501746	0.01896492
ERLN2_MOUSE	1.09780132	0.04843022
ERMIN_MOUSE	0.70136735	0.0006461
ERP29_MOUSE	1.0100245	0.95273278
ES1_MOUSE	1.0409925	0.44203393
ESTD_MOUSE	1.03124716	0.62795788
ETFA_MOUSE	1.0460668	0.43475353
ETFB_MOUSE	1.03113387	0.38415183
ETFD_MOUSE	1.04376991	0.43807702
ETHE1_MOUSE	0.97350823	0.41798135
EXC6B_MOUSE	0.9326987	0.25123265
EXOC1_MOUSE	0.95258354	0.30967313
EXOC2_MOUSE	1.07266349	0.08495018
EXOC3_MOUSE	1.03025299	0.59908017
EXOC4_MOUSE	1.05356086	0.40041786
EXOC5_MOUSE	0.90683323	0.17298168
EXOC7_MOUSE	1.00110641	0.84174034
EXOC8_MOUSE	0.95971674	0.5372182
EXOG_MOUSE	1.10823534	0.2551025
EZRI_MOUSE	1.13008439	0.00765815
F10A1_MOUSE	0.95167764	0.21988638
F1142_MOUSE	0.92751538	0.19115024
F126B_MOUSE	1.02347194	0.68135928
F136A_MOUSE	1.13011404	0.25662989
F162A_MOUSE	1.11419842	0.37186432
F1712_MOUSE	0.89734581	0.1516368
F213A_MOUSE	1.04187096	0.36204647
FA49B_MOUSE	0.94825525	0.29022506
FA81A_MOUSE	0.89641254	0.13158071
FAAA_MOUSE	1.07930457	0.32457786
FAAH1_MOUSE	1.18543021	0.42186774
FABP5_MOUSE	1.06887186	0.32985143
FABP7_MOUSE	0.89541554	0.01497807

FABPH_MOUSE	0.97068313	0.38553989
FAD1_MOUSE	0.95526062	0.55876374
FAF2_MOUSE	1.11270418	0.4015437
FAHD1_MOUSE	1.04220832	0.75349186
FAHD2_MOUSE	1.00052738	0.82713292
FAK1_MOUSE	0.90546026	0.03915314
FAK2_MOUSE	0.86129274	0.01378658
FARP1_MOUSE	1.08695481	0.37927058
FAS_MOUSE	1.00228816	0.9294778
FBP1L_MOUSE	1.25682396	0.39202978
FBX2_MOUSE	0.89586835	0.05059159
FBX41_MOUSE	1.084194	0.08931452
FERM2_MOUSE	1.01558709	0.7528284
FGF1_MOUSE	1.00311236	0.96833547
FIS1_MOUSE	1.05631133	0.96275341
FKB1A_MOUSE	0.99037756	0.64307739
FKBP2_MOUSE	1.09562595	0.07091616
FKBP4_MOUSE	1.06555138	0.442951
FKBP8_MOUSE	1.0166283	0.83882213
FLOT1_MOUSE	1.0500364	0.2668803
FLOT2_MOUSE	1.03616288	0.15662698
FNTA_MOUSE	1.25514043	0.10376756
FPPS_MOUSE	0.97889044	0.52279687
FRIH_MOUSE	0.98876314	0.65045371
FRS1L_MOUSE	0.94665898	0.29738819
FSCN1_MOUSE	1.00096874	0.85630928
FSD1_MOUSE	1.08443745	0.32867667
FUMH_MOUSE	1.02945542	0.69250377
FUND2_MOUSE	0.98019326	0.63108954
FXL16_MOUSE	0.95774399	0.36142269
FXYD6_MOUSE	1.00550852	0.56875657
FYN_MOUSE	1.13212404	0.02046221
G3P_MOUSE	1.00495118	0.91216678
G6PD1_MOUSE	0.99192471	0.8355243
G6PI_MOUSE	1.01287995	0.98114521
GABR1_MOUSE	1.00921034	0.87709605
GABR2_MOUSE	1.19471926	0.09213734
GABT_MOUSE	1.14023909	0.0939069
GAK_MOUSE	1.01875869	0.91231741

GANAB_MOUSE	1.03330722	0.34374353
GAS7_MOUSE	0.94527382	0.27295367
GBB1_MOUSE	0.98883087	0.63395604
GBB2_MOUSE	0.98445213	0.63578959
GBG12_MOUSE	1.21638441	0.13046898
GBRA1_MOUSE	1.03930183	0.51960803
GBRA3_MOUSE	1.05650769	0.54664987
GBRB2_MOUSE	1.00909209	0.65021137
GBRG2_MOUSE	1.03554937	0.60848859
GCDH_MOUSE	1.04903765	0.15152767
GCSP_MOUSE	1.00781441	0.90413185
GCYB1_MOUSE	0.9935508	0.76844967
GD1L1_MOUSE	1.01278793	0.96060401
GDAP1_MOUSE	1.05457175	0.52691484
GDE1_MOUSE	1.06358373	0.86682562
GDIA_MOUSE	0.95526691	0.35910827
GDIB_MOUSE	0.97499466	0.50050969
GDIR1_MOUSE	0.9310952	0.10893807
GDIR2_MOUSE	1.0145506	0.98386481
GDPD1_MOUSE	0.99640758	0.6984245
GELS_MOUSE	0.89959665	0.03404801
GEPH_MOUSE	0.96420145	0.12394965
GGT7_MOUSE	0.96157393	0.14200352
GHC1_MOUSE	1.02600894	0.69470951
GHC2_MOUSE	1.13098581	0.0148315
GHITM_MOUSE	1.01704093	0.82645665
GIT1_MOUSE	0.97039765	0.47000531
GLNA_MOUSE	1.02215468	0.90575509
GLO2_MOUSE	1.04258018	0.74734337
GLOD4_MOUSE	1.00728895	0.91548343
GLPK_MOUSE	0.99256418	0.62122889
GLRX3_MOUSE	1.01231348	0.87357842
GLRX5_MOUSE	1.00267135	0.69663536
GLSK_MOUSE	0.9905067	0.6601556
GLTP_MOUSE	0.80062921	0.00065311
GLU2B_MOUSE	0.95082672	0.58245884
GMFB_MOUSE	1.01674218	0.47509811
GNA11_MOUSE	0.96719908	0.35009611
GNA13_MOUSE	1.02471894	0.7575927

GNAI1_MOUSE	0.93551604	0.20103305
GNAI2_MOUSE	0.99326702	0.64699244
GNAL_MOUSE	0.91293456	0.23308404
GNAO_MOUSE	1.0334088	0.73332862
GNAQ_MOUSE	1.01520292	0.9240941
GNAS1_MOUSE	0.99259823	0.67681486
GNAZ_MOUSE	0.98146205	0.56178768
GNL1_MOUSE	1.01604335	0.9174514
GP158_MOUSE	0.97225362	0.33973596
GPD1L_MOUSE	0.98262733	0.4886019
GPDA_MOUSE	0.95424183	0.2820429
GPDM_MOUSE	1.02325067	0.59866467
GPM6A_MOUSE	1.04278309	0.65782388
GPM6B_MOUSE	0.93013583	0.20792359
GPX1_MOUSE	1.14533197	0.42531989
GPX41_MOUSE	1.00030779	0.87783759
GRAP1_MOUSE	0.90511427	0.2555977
GRB2_MOUSE	0.96697427	0.36363443
GRHPR_MOUSE	1.00215138	0.89001022
GRIA1_MOUSE	0.9917401	0.63537587
GRIA2_MOUSE	0.99801825	0.80235
GRIA3_MOUSE	1.03254364	0.47479562
GRIA4_MOUSE	0.91835384	0.15364045
GRIK2_MOUSE	1.01228953	0.99953405
GRIN1_MOUSE	0.96095874	0.40598644
GRM2_MOUSE	0.98199966	0.53965784
GRM3_MOUSE	1.01481414	0.95494592
GRM5_MOUSE	0.98004857	0.73559814
GRP75_MOUSE	0.98695	0.57954
GRP78_MOUSE	0.99697679	0.75812564
GRPE1_MOUSE	0.96205474	0.41323019
GSH1_MOUSE	0.96178101	0.43616771
GSHB_MOUSE	0.85033172	0.02268042
GSHR_MOUSE	1.02567347	0.7619371
GSK3A_MOUSE	0.96525689	0.35794847
GSK3B_MOUSE	0.98757273	0.52690272
GSLG1_MOUSE	0.95830048	0.73752093
GSTA4_MOUSE	1.02662765	0.75157663
GSTK1_MOUSE	1.16557025	0.01233682

GSTM1_MOUSE	1.15518006	0.08652935
GSTM5_MOUSE	0.95707989	0.08519237
GSTM7_MOUSE	1.03962189	0.97102673
GSTO1_MOUSE	0.97255265	0.40249991
GSTP1_MOUSE	1.01889342	0.97019997
GTR1_MOUSE	1.03829364	0.70525455
GTR3_MOUSE	0.97449832	0.4580693
GUAA_MOUSE	0.98635927	0.54324771
GUAD_MOUSE	0.9358393	0.0666416
H2B1B_MOUSE	1.80862575	0.09714643
H4_MOUSE	1.41146582	0.21945612
HACD3_MOUSE	1.09368568	0.30131823
HBA_MOUSE	0.9966499	0.74706481
HBB1_MOUSE	0.96325363	0.57335766
HCD2_MOUSE	1.0063288	0.93645434
HCDH_MOUSE	1.12175114	0.31525542
HCN1_MOUSE	0.94301013	0.48815647
HCN2_MOUSE	1.15046562	0.18074168
HD_MOUSE	0.91220661	0.12669536
HDHD2_MOUSE	1.16675473	0.14740017
HDHD3_MOUSE	1.19753055	0.08072622
HEBP1_MOUSE	0.82502717	0.01120527
HECAM_MOUSE	1.00378752	0.6768445
HEM2_MOUSE	1.04526424	0.97466326
HEM6_MOUSE	0.87976637	0.01536622
HEMH_MOUSE	1.06645626	0.30082923
HEXB_MOUSE	1.25357201	7.8042E-05
HGS_MOUSE	0.94769661	0.40013508
HIBCH_MOUSE	1.0059509	0.90455214
HINT1_MOUSE	1.02699273	0.93559054
HINT2_MOUSE	1.13807736	0.17038312
HIP1R_MOUSE	1.07038403	0.03480123
HMGCL_MOUSE	1.0969999	0.18029747
HMOX2_MOUSE	1.01384104	0.98304365
HNRPD_MOUSE	1.00846097	0.84607034
HNRPK_MOUSE	0.91002648	0.0314375
HNRPQ_MOUSE	0.97713969	0.35580287
HOME1_MOUSE	0.92744494	0.02917598
HOME2_MOUSE	0.96944594	0.46724973

HPCA_MOUSE	0.88978881	0.13213266
HPCL1_MOUSE	0.81808759	0.01486627
HPCL4_MOUSE	1.00947008	0.9821272
HPLN1_MOUSE	1.01779418	0.74266599
HPRT_MOUSE	0.98731662	0.64991697
HS105_MOUSE	1.01309323	0.77386254
HS12A_MOUSE	1.00111196	0.80187899
HS74L_MOUSE	0.97532925	0.52169026
HS90A_MOUSE	1.0514396	0.77030603
HS90B_MOUSE	1.05436386	0.70537067
HSDL1_MOUSE	1.16066317	0.21571423
HSDL2_MOUSE	1.19055672	0.2311899
HSP72_MOUSE	0.89959424	0.02410683
HSP74_MOUSE	0.99306137	0.72726329
HSP7C_MOUSE	0.9805482	0.4997829
HUWE1_MOUSE	1.35876657	0.13870994
HXK1_MOUSE	1.04056069	0.48317218
HYEP_MOUSE	1.35368608	0.26282332
HYES_MOUSE	1.1278738	0.11275112
HYOU1_MOUSE	1.04149257	0.33220653
ICAM5_MOUSE	1.15414603	0.18747463
IDH3A_MOUSE	1.03306397	0.79348352
IDHC_MOUSE	0.98353491	0.532206
IDHG1_MOUSE	1.05339529	0.57543681
IDHP_MOUSE	1.08284323	0.10659142
IDI1_MOUSE	0.95106736	0.36793017
IF2A_MOUSE	0.97077302	0.43973674
IF2M_MOUSE	0.95094877	0.24604319
IF4A1_MOUSE	0.97879608	0.71371297
IF4A2_MOUSE	0.99653524	0.58493436
IF4B_MOUSE	0.84114672	0.08381776
IF4G2_MOUSE	0.90824229	0.1655952
IF4G3_MOUSE	2.13082595	0.2253589
IF4H_MOUSE	0.906618	0.01605254
IF5A1_MOUSE	1.07810511	0.46918629
IGS21_MOUSE	1.02254918	0.77797533
IGSF8_MOUSE	0.90445383	0.09063181
ILDR2_MOUSE	1.16725867	0.0204878
ILEUA_MOUSE	0.90670093	0.15485945

IMA4_MOUSE	1.07562575	0.34381595
IMB1_MOUSE	0.96736532	0.22525699
IMPA1_MOUSE	0.94045773	0.06342261
INP4A_MOUSE	1.19387803	0.15036698
INPP_MOUSE	0.93495993	0.24798857
IP3KA_MOUSE	1.03272533	0.29738274
IPO5_MOUSE	1.03547547	0.50682953
IPO7_MOUSE	0.96886146	0.46091063
IPO9_MOUSE	1.12071018	0.62653297
IPYR_MOUSE	0.90182479	0.11972807
IPYR2_MOUSE	1.03919807	0.70320786
IQEC1_MOUSE	1.01964223	0.84060832
IQEC2_MOUSE	0.97027953	0.23230766
IQEC3_MOUSE	0.96746136	0.3646891
ISC2A_MOUSE	1.45600583	0.07998865
ISCA1_MOUSE	0.96274718	0.38011298
ISCA2_MOUSE	0.97379008	0.39297602
ISCU_MOUSE	1.00502571	0.92568684
ITAV_MOUSE	1.07288518	0.49259874
ITPA_MOUSE	1.01960454	0.99858423
ITPR1_MOUSE	0.94623038	0.40546749
ITSN1_MOUSE	0.90674873	0.13140552
IVD_MOUSE	1.01863486	0.58514458
JAM3_MOUSE	0.82781692	0.00443631
JIP3_MOUSE	0.98153519	0.47927761
K0513_MOUSE	0.92586537	0.05359384
K1107_MOUSE	0.98621988	0.41971006
K1468_MOUSE	0.94151881	0.21647293
KAD1_MOUSE	0.90544477	0.05105984
KAD3_MOUSE	1.07099764	0.07619417
KAD4_MOUSE	1.09068021	0.29152758
KAD5_MOUSE	0.88686762	0.21673854
KALRN_MOUSE	0.97906843	0.48625297
KAP0_MOUSE	1.00790209	0.86173492
KAP2_MOUSE	0.9940229	0.69684
KAP3_MOUSE	1.05555251	0.30122207
KAPCA_MOUSE	0.98782347	0.676332
KAPCB_MOUSE	1.13611473	0.30706864
KAT3_MOUSE	1.06803927	0.62888836

KBTBB_MOUSE	1.01297014	0.91865502
KCAB2_MOUSE	1.14419735	0.01641592
KCC1D_MOUSE	0.92286057	0.20861129
KCC2A_MOUSE	0.94621948	0.2878846
KCC2B_MOUSE	0.93683967	0.23063677
KCC2D_MOUSE	0.99126464	0.7451315
KCC2G_MOUSE	0.88936051	0.06989424
KCC4_MOUSE	0.86225744	0.00240262
KCD12_MOUSE	0.89011137	0.09686777
KCD16_MOUSE	1.1265359	0.19257008
KCJ10_MOUSE	1.18909646	0.01845462
KCMA1_MOUSE	1.07136577	0.09498372
KCNA1_MOUSE	0.95670716	0.3306516
KCNA6_MOUSE	1.00342447	0.82905461
KCNC3_MOUSE	0.99013502	0.76865145
KCND2_MOUSE	0.96983282	0.59478545
KCRB_MOUSE	0.95922771	0.24147638
KCRU_MOUSE	0.98397	0.59662836
KCY_MOUSE	0.96140981	0.20306035
KI21A_MOUSE	1.01049604	0.94805992
KIF1A_MOUSE	0.94575693	0.14319105
KIF2A_MOUSE	0.99218939	0.70145481
KIF5C_MOUSE	0.99174493	0.63416541
KINH_MOUSE	0.98484243	0.59842804
KKCC2_MOUSE	0.89425397	0.05757814
KLC1_MOUSE	0.922739	0.14459159
KLC2_MOUSE	1.03582973	0.76964758
KPCA_MOUSE	1.05799711	0.63840216
KPCB_MOUSE	0.99422957	0.68465005
KPCE_MOUSE	0.92950432	0.07828037
KPCG_MOUSE	0.94592617	0.25412851
KPRB_MOUSE	0.97140031	0.56822374
KPYM_MOUSE	0.9950205	0.74245051
KTN1_MOUSE	0.93140812	0.49453738
L1CAM_MOUSE	0.95412136	0.14742163
L2GL1_MOUSE	0.93869431	0.10888802
L2HDH_MOUSE	1.02902983	0.77627952
LACTB_MOUSE	0.99047184	0.75012811
LAMP1_MOUSE	1.40152611	0.00716468

LAMP2_MOUSE	1.26534597	0.03109832
LANC1_MOUSE	1.00586194	0.85129628
LANC2_MOUSE	0.96555558	0.37886485
LASP1_MOUSE	1.07232237	0.45671315
LAT1_MOUSE	1.09288664	0.32592752
LDHA_MOUSE	1.0037108	0.8738417
LDHB_MOUSE	1.00644869	0.93920366
LEGL_MOUSE	1.02286825	0.58196224
LETM1_MOUSE	0.96141347	0.28213711
LGI1_MOUSE	1.01443788	0.88293982
LGI3_MOUSE	0.98087268	0.64846702
LGUL_MOUSE	0.88465545	0.02776196
LIGO1_MOUSE	0.92535483	0.0662017
LIN7A_MOUSE	1.04419579	0.60689541
LIN7B_MOUSE	1.01568644	0.97559783
LIN7C_MOUSE	0.95853856	0.35366281
LIPA2_MOUSE	1.02838839	0.9503486
LIPA3_MOUSE	0.9300342	0.18815451
LIS1_MOUSE	0.9655425	0.35960492
LKHA4_MOUSE	1.03751804	0.73717574
LNEBL_MOUSE	0.98516024	0.66806
LNP_MOUSE	0.91369094	0.1144901
LONM_MOUSE	1.00933699	0.97320616
LPPRC_MOUSE	1.06369256	0.12686758
LRC47_MOUSE	0.93331977	0.21386983
LRC4B_MOUSE	0.94974307	0.25142476
LRC57_MOUSE	1.00165862	0.76285802
LRC59_MOUSE	0.92082264	0.27640336
LRC8A_MOUSE	1.04081756	0.74252674
LRP1_MOUSE	1.05107247	0.26354775
LRRC7_MOUSE	0.94852172	0.21733151
LRRT4_MOUSE	0.93034258	0.12192532
LSAMP_MOUSE	1.0151648	0.95933566
LXN_MOUSE	0.98544172	0.61593876
LY6H_MOUSE	0.73551181	0.0012169
LYAG_MOUSE	0.96022741	0.4989338
LYPA2_MOUSE	1.62648895	0.16854832
M2OM_MOUSE	1.02219577	0.84290835
MA2C1_MOUSE	1.06035861	0.96505014

MA7D2_MOUSE	0.80489912	0.00460827
MAAI_MOUSE	1.15831227	0.08578306
MACF1_MOUSE	0.94708023	0.32818398
MADD_MOUSE	0.98573014	0.70255617
MAG_MOUSE	0.86701761	0.03794073
MAGI2_MOUSE	0.98109207	0.53730691
MAOM_MOUSE	1.01600988	0.86977351
MAON_MOUSE	1.00489047	0.84958449
MAOX_MOUSE	1.06690901	0.12073121
MAP1A_MOUSE	0.92195386	0.00325784
MAP1B_MOUSE	0.9432258	0.12478056
MAP1S_MOUSE	0.95264332	0.17528012
MAP4_MOUSE	0.89066556	0.01688194
MAP6_MOUSE	0.92475347	0.09264398
MARC2_MOUSE	1.11923826	0.03377912
MARCS_MOUSE	0.97101937	0.58943751
MARE1_MOUSE	1.04629156	0.30955014
MARE2_MOUSE	0.9926666	0.77606803
MARE3_MOUSE	0.98540773	0.58928631
MARK2_MOUSE	1.02383251	0.85802327
MAT2B_MOUSE	0.93985703	0.23022174
MBLC2_MOUSE	0.90343498	0.07978019
MBP_MOUSE	0.92328741	0.4454055
MCAT_MOUSE	1.04065071	0.47650239
MCCA_MOUSE	1.02846836	0.77068371
MCCB_MOUSE	1.07818015	0.362468
MCTS1_MOUSE	1.2107085	0.50599744
MCU_MOUSE	1.0260794	0.66581334
MDHC_MOUSE	0.97076836	0.37386438
MDHM_MOUSE	1.00730397	0.96203795
MECR_MOUSE	1.27092876	0.0299552
METK2_MOUSE	1.05221617	0.98342534
MFF_MOUSE	1.1429367	0.08036632
MFN2_MOUSE	1.01648063	0.95663788
MFR1L_MOUSE	0.94501509	0.19059654
MGLL_MOUSE	1.08016729	0.50201274
MGST3_MOUSE	1.1904103	0.15188327
MIA40_MOUSE	0.99153095	0.74890825
MIC13_MOUSE	1.11846878	0.18306348

MIC19_MOUSE	0.99062762	0.62120066
MIC25_MOUSE	1.04625483	0.74891666
MIC26_MOUSE	1.02690877	0.89991855
MIC27_MOUSE	1.0358291	0.2455327
MIC60_MOUSE	0.96442481	0.33929642
MICA3_MOUSE	0.88265171	0.04276538
MICU1_MOUSE	0.96058267	0.37202076
MICU3_MOUSE	1.03362737	0.7407846
MIF_MOUSE	1.01136338	0.8920451
MINK1_MOUSE	1.05338813	0.26692958
MIRO1_MOUSE	0.94261506	0.1258291
MIRO2_MOUSE	1.12920101	0.06287708
MK01_MOUSE	1.01717077	0.83954249
MK03_MOUSE	0.99686119	0.88195385
ML12B_MOUSE	0.90102778	0.07550757
MLC1_MOUSE	1.08359027	0.75267065
MLEC_MOUSE	1.00699818	0.8871345
MMSA_MOUSE	1.07307357	0.14809918
MOES_MOUSE	1.0951602	0.4149014
MOG_MOUSE	0.81062557	0.04203528
MOT1_MOUSE	1.11464269	0.75318346
MP2K1_MOUSE	0.98046129	0.58968413
MP2K2_MOUSE	0.9120673	0.03546896
MP2K4_MOUSE	1.02065888	0.92241121
MPC2_MOUSE	1.32217013	0.00070535
MPCP_MOUSE	1.06910064	0.12559273
MPI_MOUSE	0.97964985	0.65587716
MPP2_MOUSE	1.11963475	0.04303153
MPP3_MOUSE	0.94265337	0.10859245
MPP6_MOUSE	1.05433544	0.54897412
MPPA_MOUSE	1.02924742	0.74045795
MPPB_MOUSE	1.12859517	0.74639868
MRCKB_MOUSE	0.93370468	0.03820849
MSRA_MOUSE	1.56066202	0.24394602
MTAP2_MOUSE	0.96096004	0.29523747
MTCH1_MOUSE	0.97208549	0.42474593
MTCH2_MOUSE	1.02016669	0.75168631
MTEF2_MOUSE	1.17645415	0.24683986
MTMR1_MOUSE	0.93901725	0.14697626

MTMR2_MOUSE	0.90768323	0.16139349
MTMR5_MOUSE	0.97853735	0.46341999
MTOR_MOUSE	1.03595867	0.8085487
MTPN_MOUSE	0.95693534	0.2902891
MTX1_MOUSE	1.00711524	0.79443812
MTX2_MOUSE	1.03237818	0.64176554
MUTA_MOUSE	1.00531712	0.95113874
MY18A_MOUSE	1.02635245	0.86903555
MYCT_MOUSE	0.95986112	0.49220702
MYH10_MOUSE	0.9592996	0.36358753
MYH14_MOUSE	0.9453957	0.30542759
MYH9_MOUSE	0.92656046	0.30806453
MYL6_MOUSE	0.90022221	0.04429835
MYO1D_MOUSE	0.87790012	0.13191371
MYO5A_MOUSE	0.99772778	0.83136478
MYO6_MOUSE	0.92565205	0.16830803
MYPR_MOUSE	0.89549461	0.05094985
NAC1_MOUSE	1.06939221	0.26206924
NAC2_MOUSE	1.00869748	0.96067254
NACA_MOUSE	0.86900417	0.02119228
NAGAB_MOUSE	0.85394623	0.02971335
NAGK_MOUSE	1.10209212	0.68275629
NAKD2_MOUSE	1.03332278	0.30706702
NAMPT_MOUSE	1.11718972	0.43285243
NB5R1_MOUSE	1.09370359	0.24967383
NB5R3_MOUSE	1.0399061	0.25344097
NBEA_MOUSE	1.02745106	0.73967195
NCALD_MOUSE	0.8888428	0.05941141
NCAM1_MOUSE	1.00922019	0.93701912
NCAM2_MOUSE	1.06230671	0.06651004
NCAN_MOUSE	0.92976484	0.26067608
NCDN_MOUSE	0.97663871	0.29288803
NCEH1_MOUSE	1.01867947	0.63870012
NCKP1_MOUSE	1.02066555	0.68898707
NCPR_MOUSE	1.04230958	0.55276667
NCS1_MOUSE	1.02989954	0.90692259
NDKA_MOUSE	1.058308	0.16941974
NDKB_MOUSE	1.12502218	0.40143463
NDRG1_MOUSE	0.94785859	0.442828

NDRG2_MOUSE	0.99018196	0.64431898
NDRG3_MOUSE	0.89548669	0.08100714
NDRG4_MOUSE	0.95069216	0.27890409
NDUA1_MOUSE	1.0132651	0.88219742
NDUA2_MOUSE	1.03051931	0.95158121
NDUA4_MOUSE	1.14503956	0.00412785
NDUA5_MOUSE	1.12733723	0.27634888
NDUA6_MOUSE	1.08844412	0.17983409
NDUA7_MOUSE	0.98251045	0.64620272
NDUA8_MOUSE	1.07774647	0.28945013
NDUA9_MOUSE	1.15779457	0.16088299
NDUAA_MOUSE	1.06895499	0.30846433
NDUAB_MOUSE	1.12938014	0.12287275
NDUAC_MOUSE	1.0793315	0.25434492
NDUAD_MOUSE	1.0688768	0.20339444
NDUB1_MOUSE	1.12926102	0.13593936
NDUB3_MOUSE	1.08217393	0.29072781
NDUB4_MOUSE	1.07374921	0.379376
NDUB5_MOUSE	0.97754016	0.48708157
NDUB6_MOUSE	1.06170335	0.45320348
NDUB7_MOUSE	1.10087822	0.22268906
NDUB8_MOUSE	0.91689878	0.03792883
NDUB9_MOUSE	1.31423046	0.17222648
NDUBA_MOUSE	0.98479154	0.55529063
NDUBB_MOUSE	1.00731954	0.95369061
NDUC2_MOUSE	1.06758252	0.44422729
NDUF3_MOUSE	1.07974234	0.42007626
NDUS1_MOUSE	0.96650326	0.28283496
NDUS2_MOUSE	1.0630756	0.73636905
NDUS3_MOUSE	1.02627792	0.83733642
NDUS4_MOUSE	1.00218457	0.89041652
NDUS5_MOUSE	1.01526487	0.88259846
NDUS6_MOUSE	1.06807777	0.51588702
NDUS7_MOUSE	1.09640032	0.3709634
NDUS8_MOUSE	0.99676192	0.76320379
NDUV1_MOUSE	1.07947914	0.33487841
NDUV2_MOUSE	0.99474737	0.68649134
NEB2_MOUSE	0.93550828	0.21287939
NECA2_MOUSE	0.87940408	0.03012099

NECP1_MOUSE	0.95406575	0.21445126
NEDD4_MOUSE	0.84642226	0.12338064
NEGR1_MOUSE	0.94515288	0.1781445
NEO1_MOUSE	0.97520147	0.69255203
NEUL_MOUSE	0.91627141	0.05404982
NEUM_MOUSE	0.95603322	0.32362414
NF1_MOUSE	0.95071121	0.10359784
NFASC_MOUSE	1.02754702	0.74451862
NFL_MOUSE	1.20207472	0.0390092
NFM_MOUSE	1.20289993	0.05015881
NFS1_MOUSE	1.05719788	0.62294388
NFU1_MOUSE	0.89839858	0.06465322
NGEF_MOUSE	1.02519579	0.4896533
NHRF1_MOUSE	1.04269501	0.72023942
NICA_MOUSE	1.04294497	0.39422951
NIF3L_MOUSE	1.09041936	0.18156027
NIPS1_MOUSE	1.06533237	0.51417049
NIPS2_MOUSE	1.04139721	0.69284516
NIT1_MOUSE	1.10078875	0.23534919
NIT2_MOUSE	1.13577144	0.16474237
NLGN3_MOUSE	1.05900744	0.17582176
NLTP_MOUSE	1.06173948	0.1235345
NMDE1_MOUSE	0.97037595	0.38307683
NMDE2_MOUSE	0.95183862	0.33109324
NMDZ1_MOUSE	0.95947969	0.27012383
NMT2_MOUSE	1.06267801	0.901335
NNRD_MOUSE	0.98494453	0.5806195
NNRE_MOUSE	1.00790381	0.88367596
NNTM_MOUSE	6.67564333	0.05216051
NOE1_MOUSE	0.92599746	0.06978157
NOMO1_MOUSE	0.97066183	0.46179249
NP1L1_MOUSE	0.88747556	0.02188194
NP1L4_MOUSE	0.85882754	0.01284868
NPL4_MOUSE	0.97171711	0.62481358
NPS3B_MOUSE	1.13234199	0.11440893
NPTN_MOUSE	1.03143162	0.66149391
NPTX1_MOUSE	0.90833515	0.01447777
NPTXR_MOUSE	0.88738283	0.07150321
NRCAM_MOUSE	1.14839269	0.31441831

NRX1A_MOUSE	1.01682435	0.94817938
NRX3A_MOUSE	1.00566944	0.95306512
NSF_MOUSE	0.99322478	0.65001745
NSF1C_MOUSE	0.95379788	0.26623312
NT5D3_MOUSE	1.05916555	0.21744058
NTF2_MOUSE	1.1785295	0.05905976
NTRI_MOUSE	0.98825748	0.57368287
NTRK2_MOUSE	1.09437487	0.3594508
NU1M_MOUSE	1.15263395	0.39224592
NU5M_MOUSE	1.16804406	0.24218301
NUCG_MOUSE	0.98424953	0.65962729
NUDC_MOUSE	1.2086636	0.00217095
NUDT3_MOUSE	0.92530622	0.06406152
OAT_MOUSE	1.12313648	0.24280878
OCAD1_MOUSE	0.92184018	0.08040909
ODB2_MOUSE	1.1042464	0.07019949
ODBA_MOUSE	0.99953326	0.83828165
ODBB_MOUSE	1.00248265	0.63956314
ODO1_MOUSE	1.02134296	0.86854725
ODO2_MOUSE	0.94457978	0.09527402
ODP2_MOUSE	0.96444011	0.20698769
ODPA_MOUSE	1.0249564	0.66129171
ODPB_MOUSE	0.99904408	0.76815612
ODPX_MOUSE	0.95428042	0.03605176
OGA_MOUSE	1.05758816	0.37620006
OGT1_MOUSE	0.99876822	0.84780331
OLA1_MOUSE	1.10552791	0.2503684
OMGP_MOUSE	0.94813176	0.40692267
OMP_MOUSE	0.75077186	0.07260789
OPA1_MOUSE	0.97592061	0.32270261
OSB10_MOUSE	0.98453597	0.68213975
OSBL1_MOUSE	1.02333705	0.89751222
OSBL8_MOUSE	0.99106234	0.65315863
OSCP1_MOUSE	1.05345987	0.61866299
OST48_MOUSE	1.01504547	0.97785333
OTUB1_MOUSE	0.95155946	0.36339172
OX2G_MOUSE	1.04870317	0.55787902
OXR1_MOUSE	0.96822046	0.37130483
OXSR1_MOUSE	0.90056682	0.10668183

P5CR2_MOUSE	1.02113577	0.86756015
P5CS_MOUSE	0.95373377	0.2186305
PA1B2_MOUSE	1.02100765	0.79967824
PA2G4_MOUSE	0.94860807	0.05160568
PABP1_MOUSE	0.95953523	0.19427743
PACN1_MOUSE	0.85681121	0.03486825
PACN2_MOUSE	0.9578468	0.46600372
PACS1_MOUSE	0.97262035	0.30162945
PACS2_MOUSE	1.0461893	0.91913398
PADI2_MOUSE	0.9481439	0.45997087
PAK1_MOUSE	0.94763079	0.25417587
PALM_MOUSE	0.93079139	0.2173068
PALM2_MOUSE	1.10413941	0.88475218
PARK7_MOUSE	0.95404056	0.18930165
PCBP1_MOUSE	1.00713177	0.95802775
PCBP2_MOUSE	0.93977297	0.08755091
PCCA_MOUSE	0.95424421	0.20346456
PCCB_MOUSE	1.00090596	0.81193898
PCKGM_MOUSE	0.95715302	0.25383051
PCLO_MOUSE	1.01693286	0.94870891
PCSK1_MOUSE	0.91762521	0.21548108
PCY2_MOUSE	0.96174223	0.37457413
PCYOX_MOUSE	1.15507254	0.17640501
PDC6I_MOUSE	0.95421676	0.24845789
PDCD5_MOUSE	0.76320979	0.00275629
PDCD6_MOUSE	0.97101167	0.36312258
PDE10_MOUSE	1.11043813	0.08992869
PDE1A_MOUSE	0.89786772	0.04629677
PDE1B_MOUSE	1.00526591	0.93548675
PDE2A_MOUSE	1.04812793	0.33920883
PDIA1_MOUSE	1.00575048	0.94270369
PDIA3_MOUSE	0.9756158	0.57172246
PDIA4_MOUSE	0.95474027	0.28739388
PDIA6_MOUSE	0.99920898	0.83359917
PDIP2_MOUSE	0.9673986	0.44706677
PDK1_MOUSE	0.93729388	0.14992757
PDK2_MOUSE	0.93077913	0.27097819
PDK3_MOUSE	0.93361175	0.15337018
PDP1_MOUSE	1.06257359	0.28100377

PDPR_MOUSE	1.00762373	0.96004196
PDXK_MOUSE	1.10611712	0.36480152
PEA15_MOUSE	1.36824969	0.61901669
PEBP1_MOUSE	0.93651747	0.0171186
PEX5R_MOUSE	0.97945021	0.47805376
PFD3_MOUSE	1.02434824	0.92730139
PFD5_MOUSE	0.96707755	0.3138578
PFD6_MOUSE	0.96528723	0.36871092
PFKAL_MOUSE	1.05272213	0.32877523
PFKAM_MOUSE	0.98169945	0.58441908
PFKAP_MOUSE	1.02725679	0.61691882
PGAM1_MOUSE	0.99935061	0.81874243
PGAM5_MOUSE	0.92499511	0.03368299
PGCB_MOUSE	1.13612543	0.30486573
PGES2_MOUSE	0.96041077	0.45285622
PGFS_MOUSE	1.06252775	0.18184908
PGK1_MOUSE	1.03335994	0.65176088
PGM1_MOUSE	1.0370261	0.64439547
PGM2L_MOUSE	0.94145415	0.07355529
PGP_MOUSE	0.93352441	0.06661916
PGPS1_MOUSE	1.05431775	0.45114758
PGRC1_MOUSE	0.96979417	0.50456131
PGRC2_MOUSE	0.89370458	0.06610713
PGTA_MOUSE	1.02648338	0.77741789
PHAR1_MOUSE	0.93084472	0.05922947
PHB_MOUSE	0.9865244	0.48785354
PHB2_MOUSE	1.06724223	0.33035951
PHF24_MOUSE	1.07333765	0.56219408
PHIPL_MOUSE	0.95514206	0.23890019
PHP14_MOUSE	0.9886719	0.595984
PHYIP_MOUSE	0.96376064	0.4341621
PI3R4_MOUSE	0.89167808	0.02270654
PI42A_MOUSE	0.94273072	0.10806918
PI42B_MOUSE	0.97617478	0.48190734
PI42C_MOUSE	1.00358304	0.80108792
PI4KA_MOUSE	1.05584289	0.3535877
PI51C_MOUSE	0.96523372	0.50100504
PICAL_MOUSE	0.96834221	0.38879483
PIMT_MOUSE	0.92375817	0.08782141

PIN1_MOUSE	1.06597268	0.44592309
PIPNA_MOUSE	0.98535021	0.61805095
PIPNB_MOUSE	1.02701707	0.88147985
PITM1_MOUSE	0.94288814	0.16538686
PK3C3_MOUSE	1.04634065	0.73536577
PKP4_MOUSE	1.03996398	0.57660734
PLAP_MOUSE	0.9774675	0.57516115
PLBL2_MOUSE	0.84508706	0.01699015
PLCB1_MOUSE	1.00613749	0.77020676
PLCG1_MOUSE	1.29537503	0.08387851
PLCX3_MOUSE	1.14424476	0.12723393
PLD3_MOUSE	0.97168916	0.48260632
PLEC_MOUSE	1.07899567	0.2027478
PLPL8_MOUSE	1.05821826	0.20074931
PLPP_MOUSE	1.04769601	0.42258384
PLPP3_MOUSE	1.15348835	0.03520309
PLPR4_MOUSE	0.98114661	0.61911718
PLRKT_MOUSE	0.99214784	0.75749344
PLSL_MOUSE	0.92644802	0.13896414
PLST_MOUSE	0.97279865	0.46286432
PLXA1_MOUSE	1.02748703	0.5906007
PLXA4_MOUSE	1.00085776	0.94848269
PLXB2_MOUSE	1.17344504	0.33905113
PMM1_MOUSE	0.93177184	0.31012612
PNPH_MOUSE	0.98586192	0.71419673
PNPT1_MOUSE	0.96942518	0.43589907
PP1A_MOUSE	1.0420291	0.44524157
PP1R7_MOUSE	0.9492785	0.23359999
PP2AA_MOUSE	0.98023118	0.57012664
PP2BA_MOUSE	0.95650745	0.1996156
PP2BB_MOUSE	0.92236217	0.06922296
PPAC_MOUSE	0.89862769	0.08841646
PPCE_MOUSE	0.88941128	0.11350971
PPCEL_MOUSE	0.91241081	0.20774792
PPGB_MOUSE	0.99277351	0.73645119
PPIA_MOUSE	0.9050587	0.01508338
PPIB_MOUSE	1.00714709	0.9466152
PPID_MOUSE	1.01164918	0.71325911
PPM1A_MOUSE	1.05648944	0.86269732

PPM1H_MOUSE	1.04288989	0.74915372
PPME1_MOUSE	0.94068711	0.10783277
PPP5_MOUSE	0.98668627	0.6034343
PPR21_MOUSE	0.91015258	0.24976518
PPR29_MOUSE	0.95123548	0.15439529
PPTC7_MOUSE	0.99441218	0.69922397
PRAF3_MOUSE	1.22893042	0.00097627
PRDX1_MOUSE	0.99648674	0.7490284
PRDX2_MOUSE	1.0048277	0.94308113
PRDX3_MOUSE	1.04002467	0.68350605
PRDX5_MOUSE	0.99238561	0.64325564
PRDX6_MOUSE	1.06887752	0.07476094
PREB_MOUSE	1.0305386	0.76752477
PREP_MOUSE	1.00554792	0.96084984
PREX1_MOUSE	1.07314831	0.27822383
PRIO_MOUSE	1.2152459	0.31692789
PROD_MOUSE	1.33184912	0.00683403
PROF1_MOUSE	0.99089886	0.68098301
PROF2_MOUSE	0.97319225	0.32864379
PRPS1_MOUSE	1.19313586	0.23268953
PRPTZ_MOUSE	0.94046504	0.32248869
PRRT2_MOUSE	0.93747582	0.10437303
PRRT3_MOUSE	0.98371067	0.59608915
PRS10_MOUSE	0.93512359	0.29038965
PRS4_MOUSE	1.02088613	0.85935263
PRS6A_MOUSE	0.91731684	0.17566909
PRS6B_MOUSE	1.00362165	0.86879271
PRS7_MOUSE	1.0002942	0.78615279
PRS8_MOUSE	1.02220178	0.75874328
PSA_MOUSE	1.00303462	0.89270577
PSA1_MOUSE	1.02998195	0.69003613
PSA2_MOUSE	1.08071953	0.61203594
PSA3_MOUSE	1.03955006	0.41789878
PSA4_MOUSE	0.97312791	0.42161853
PSA5_MOUSE	1.02069557	0.43074498
PSA6_MOUSE	0.97606106	0.35994854
PSA7_MOUSE	0.95031584	0.30712505
PSB1_MOUSE	0.97220788	0.53312792
PSB2_MOUSE	1.02259758	0.70455866

PSB3_MOUSE	1.03650417	0.60703967
PSB4_MOUSE	1.09031281	0.3767386
PSB5_MOUSE	1.06300824	0.54682094
PSB7_MOUSE	1.14197811	0.2033687
PSD11_MOUSE	0.98031312	0.55340841
PSD12_MOUSE	0.95514434	0.41985889
PSD13_MOUSE	1.02813493	0.65293081
PSD3_MOUSE	0.96496766	0.20095554
PSMD1_MOUSE	1.016873	0.79713294
PSMD2_MOUSE	1.00111208	0.88348815
PSMD3_MOUSE	1.02822311	0.36964501
PSMD4_MOUSE	0.90764278	0.09602632
PSMD5_MOUSE	1.09648268	0.71265602
PSMD6_MOUSE	1.10318572	0.03722242
PSMD8_MOUSE	0.96818102	0.56338811
PSMD9_MOUSE	1.00634363	0.87850663
PSME1_MOUSE	0.85349389	0.02720591
PTCD3_MOUSE	0.92077942	0.07914372
PTGR3_MOUSE	0.98930205	0.77100447
PTH2_MOUSE	1.40128853	0.03860765
PTN11_MOUSE	1.11394948	0.35950536
PTN5_MOUSE	1.02792282	0.75957576
PTPA_MOUSE	0.91739892	0.01995569
PTPR2_MOUSE	1.05497927	0.21468762
PTPRA_MOUSE	0.95736181	0.36575842
PTPRD_MOUSE	1.00970418	0.98280693
PTPRS_MOUSE	0.97560704	0.46096821
PUR6_MOUSE	0.96800801	0.44984594
PUR9_MOUSE	1.04132235	0.84339459
PURA_MOUSE	0.9363025	0.20114416
PURB_MOUSE	1.05287647	0.71801766
PYC_MOUSE	1.08013427	0.13587142
PYGB_MOUSE	1.08392465	0.01500789
PYGM_MOUSE	1.112087	0.02193498
QCR1_MOUSE	1.01169733	0.98801884
QCR2_MOUSE	0.98791128	0.61404823
QCR7_MOUSE	0.95733574	0.25947284
QCR8_MOUSE	1.09664253	0.65418238
QCR9_MOUSE	1.15712494	0.12183419

QORL2_MOUSE	1.44243371	0.1082091
RAB10_MOUSE	1.20621044	0.09095565
RAB12_MOUSE	1.08023048	0.04125747
RAB14_MOUSE	0.99635397	0.79720283
RAB18_MOUSE	1.00468172	0.70458126
RAB1A_MOUSE	0.90731352	0.06284606
RAB1B_MOUSE	0.95186358	0.18105308
RAB21_MOUSE	1.07540456	0.5658942
RAB23_MOUSE	1.01080441	0.91200033
RAB2A_MOUSE	0.98936085	0.59449412
RAB35_MOUSE	0.99362191	0.77694908
RAB3A_MOUSE	0.97120894	0.49888111
RAB3B_MOUSE	1.07653686	0.68951256
RAB3C_MOUSE	1.0447865	0.98895973
RAB4B_MOUSE	0.97944705	0.57335125
RAB5A_MOUSE	1.08860547	0.27483661
RAB5B_MOUSE	0.94031537	0.1614421
RAB5C_MOUSE	1.01405013	0.83503063
RAB6A_MOUSE	0.97803469	0.57123781
RAB6B_MOUSE	0.96473644	0.38382059
RAB7A_MOUSE	1.00676915	0.83160283
RAB8A_MOUSE	1.67570226	0.06205517
RABE1_MOUSE	0.85418023	0.05176806
RABL6_MOUSE	0.94818092	0.42021216
RAC1_MOUSE	1.0583443	0.83299825
RACK1_MOUSE	0.97590529	0.59709999
RADI_MOUSE	1.01189008	0.87431125
RALA_MOUSE	1.08575762	0.46861418
RAN_MOUSE	0.98695635	0.5309179
RANG_MOUSE	0.88422938	0.05793791
RAP2A_MOUSE	1.03196459	0.66591793
RAP2B_MOUSE	0.97326508	0.45540521
RASH_MOUSE	0.95613379	0.38487808
RASL1_MOUSE	0.99970923	0.89218374
RASM_MOUSE	1.10863888	0.14557324
RB11B_MOUSE	1.04332353	0.32837907
RB39B_MOUSE	0.99731684	0.7510797
RB6I2_MOUSE	0.92540326	0.11513112
RBBP9_MOUSE	1.05063415	0.73727939

RBGPR_MOUSE	0.95931623	0.28753297
RCN2_MOUSE	0.86323993	0.19989303
RD23B_MOUSE	1.03184009	0.88407089
RDH14_MOUSE	1.01932945	0.81068441
RENT1_MOUSE	1.13651544	0.32414873
RFIP2_MOUSE	0.97744292	0.53541153
RFIP5_MOUSE	0.82827197	0.004972
RFTN2_MOUSE	1.18123722	0.47660201
RGRF2_MOUSE	0.95621213	0.45574269
RGS7_MOUSE	1.06613564	0.45796778
RHG01_MOUSE	1.00414306	0.97513123
RHG32_MOUSE	0.99949117	0.83417063
RHG35_MOUSE	1.12397969	0.1681792
RHG44_MOUSE	0.99536762	0.74758901
RHOA_MOUSE	1.17126322	0.02563162
RHOB_MOUSE	1.13512493	0.03238656
RHOG_MOUSE	0.93688861	0.36250404
RIMB2_MOUSE	0.91544582	0.01857911
RIMS1_MOUSE	0.99564106	0.80008711
RINI_MOUSE	0.95266173	0.42019188
RL10_MOUSE	0.96034669	0.3751769
RL10A_MOUSE	1.00142439	0.98769481
RL12_MOUSE	0.95056575	0.34171317
RL13_MOUSE	1.28649532	0.00542686
RL14_MOUSE	1.00173738	0.93064614
RL17_MOUSE	1.13475299	0.01307892
RL21_MOUSE	0.9770437	0.56401543
RL3_MOUSE	1.05475442	0.1105932
RL38_MOUSE	0.96130319	0.51849438
RL4_MOUSE	1.02406662	0.76887408
RL5_MOUSE	1.01958825	0.87470122
RL6_MOUSE	1.04220487	0.48659376
RL7_MOUSE	1.0676172	0.40007301
RL7A_MOUSE	0.96774819	0.44636191
RL8_MOUSE	1.0821383	0.34926238
RLA0_MOUSE	0.8992624	0.01106995
RLA1_MOUSE	1.01523076	0.88680788
RLA2_MOUSE	0.87789947	0.16794652
RM01_MOUSE	1.2219823	0.04654935

RM12_MOUSE	0.85648028	0.0520232
RM41_MOUSE	0.87317058	0.03167622
RM50_MOUSE	0.89149707	0.03190142
RMD1_MOUSE	0.92883811	0.15227402
RMD3_MOUSE	0.94254166	0.15314214
RO60_MOUSE	1.11781037	0.68018988
ROBO2_MOUSE	0.82465028	0.0682884
ROCK2_MOUSE	0.95463743	0.39349243
ROGDI_MOUSE	1.0133779	0.99429488
RP3A_MOUSE	1.04420423	0.78576917
RPGF2_MOUSE	0.8806987	0.00810043
RPGF4_MOUSE	0.99700422	0.83229983
RPGP1_MOUSE	1.00151786	0.65251196
RPGP2_MOUSE	0.95419559	0.2416302
RPN1_MOUSE	1.05715667	0.595989
RPN2_MOUSE	1.04552002	0.35340898
RRAS2_MOUSE	0.98899762	0.6486666
RRFM_MOUSE	1.01450603	0.90289726
RS10_MOUSE	1.06915766	0.43863917
RS11_MOUSE	1.05037568	0.50982213
RS13_MOUSE	1.10662193	0.23947258
RS14_MOUSE	1.01254505	0.94049481
RS15A_MOUSE	1.16267155	0.03871847
RS18_MOUSE	1.03483303	0.67342642
RS19_MOUSE	0.99985735	0.72847647
RS2_MOUSE	1.03587276	0.43573595
RS21_MOUSE	0.92580756	0.17150523
RS24_MOUSE	0.86541691	0.05371257
RS27A_MOUSE	0.98427149	0.58368844
RS27L_MOUSE	1.07386022	0.19775328
RS3_MOUSE	0.99402437	0.68947858
RS3A_MOUSE	0.97666258	0.58078618
RS4X_MOUSE	1.1468839	0.03459434
RS6_MOUSE	1.02620985	0.75940861
RS7_MOUSE	1.31546195	0.06390383
RS8_MOUSE	0.95447842	0.22542809
RS9_MOUSE	1.13896196	0.02750796
RSSA_MOUSE	0.99236724	0.70690093
RT23_MOUSE	1.01594625	0.82428655

RT27_MOUSE	0.97009438	0.57369042
RT29_MOUSE	1.39907581	0.10900803
RT34_MOUSE	0.82251234	0.06443731
RT36_MOUSE	1.14315833	0.05350938
RT4I1_MOUSE	1.15681922	0.1500766
RTCB_MOUSE	1.01697094	0.59779571
RTN1_MOUSE	0.9446657	0.24741342
RTN3_MOUSE	0.88792286	0.01867472
RTN4_MOUSE	0.93562716	0.19360728
RUFY3_MOUSE	0.90360467	0.03603376
RUVB1_MOUSE	0.85495002	0.12017311
RYR2_MOUSE	0.96981415	0.45958464
S100B_MOUSE	1.07615757	0.79393762
S10AD_MOUSE	0.98142271	0.58792829
S12A2_MOUSE	0.96411232	0.36278421
S12A5_MOUSE	1.08186295	0.21279942
S14L2_MOUSE	1.31982417	0.03278987
S1PR1_MOUSE	1.07335572	0.6308228
S20A2_MOUSE	1.18114112	0.02850308
S2542_MOUSE	1.23876352	0.03489096
S2546_MOUSE	1.05755188	0.36558275
S2551_MOUSE	1.02868958	0.58016194
S27A4_MOUSE	0.99510606	0.83680909
S38A3_MOUSE	1.05171259	0.70640212
S39AA_MOUSE	0.93959314	0.40388843
S4A10_MOUSE	1.08297921	0.0026341
S4A4_MOUSE	1.03002366	0.45470768
S6A11_MOUSE	1.04479066	0.67881269
S6A17_MOUSE	0.98198456	0.55952686
S7A14_MOUSE	1.07678813	0.41793214
SAC1_MOUSE	1.05463631	0.25844817
SAHH_MOUSE	0.97767876	0.4836327
SAHH2_MOUSE	1.04947005	0.23387923
SAHH3_MOUSE	1.20571249	0.00609146
SAM50_MOUSE	1.01565246	0.8108083
SAR1B_MOUSE	1.01195537	0.90494495
SATT_MOUSE	1.27179318	0.00018258
SC22B_MOUSE	1.04644217	0.84820811
SC23A_MOUSE	1.02069283	0.70997051

SC6A1_MOUSE	1.04704687	0.44402493
SC6A9_MOUSE	1.04501706	0.99659996
SCAI_MOUSE	1.00814605	0.96001856
SCAM1_MOUSE	0.91149406	0.05312665
SCAM3_MOUSE	0.95695415	0.37448447
SCAM5_MOUSE	1.00947592	0.97083037
SCFD1_MOUSE	0.90936702	0.10148419
SCMC2_MOUSE	1.15307934	0.00565949
SCMC3_MOUSE	0.99125703	0.6576777
SCN1A_MOUSE	0.96107431	0.32573638
SCN2B_MOUSE	0.91962198	0.04399186
SCN9A_MOUSE	0.98267841	0.54947868
SCOT1_MOUSE	0.97196405	0.32820464
SCPDL_MOUSE	0.96689258	0.38797649
SCRN1_MOUSE	0.89931972	0.01521
SDHA_MOUSE	1.01064141	0.97560476
SDHB_MOUSE	1.01242014	0.87286453
SEM4A_MOUSE	1.2110218	0.22946015
SEPT11_MOUSE	1.02676427	0.89044176
SEPT2_MOUSE	1.10808339	0.03104252
SEPT3_MOUSE	0.98088199	0.43153585
SEPT4_MOUSE	0.93352012	0.34317225
SEPT5_MOUSE	0.98490978	0.53662693
SEPT6_MOUSE	0.98069654	0.49533197
SEPT7_MOUSE	0.97871305	0.56888885
SEPT8_MOUSE	1.05738837	0.3594926
SEPT9_MOUSE	0.96973026	0.16991516
SERA_MOUSE	0.92761969	0.08067915
SERC_MOUSE	0.98622933	0.66072066
SFXN1_MOUSE	1.06569193	0.36873902
SFXN3_MOUSE	0.98838765	0.60074401
SFXN5_MOUSE	1.05890098	0.59976321
SGIP1_MOUSE	1.02558421	0.63590875
SGSM1_MOUSE	1.01947642	0.8455668
SGT1_MOUSE	1.00218841	0.70255641
SGTB_MOUSE	1.04878091	0.50861808
SH3G1_MOUSE	0.81574453	0.05437591
SH3G2_MOUSE	0.84639204	0.01048259
SH3G3_MOUSE	0.82058712	0.01296663

SH3K1_MOUSE	0.85272449	0.10281332
SH3L3_MOUSE	1.07402202	0.66597029
SHAN1_MOUSE	0.98526062	0.57276709
SHAN2_MOUSE	0.96519912	0.36678548
SHAN3_MOUSE	0.96995783	0.4804189
SHLB1_MOUSE	0.90725642	0.06069977
SHLB2_MOUSE	0.92642359	0.11529593
SHPS1_MOUSE	1.07856662	0.152269
SHSA7_MOUSE	1.00105769	0.78460967
SI1L1_MOUSE	0.957487	0.3542715
SIR2_MOUSE	0.95892558	0.57611729
SIR3_MOUSE	1.12625971	0.27961822
SIR5_MOUSE	1.09770497	0.57014935
SKP1_MOUSE	0.91001624	0.09055613
SKT_MOUSE	0.99757909	0.71681481
SL9A1_MOUSE	1.03318016	0.69987297
SLIRP_MOUSE	1.03305584	0.51598989
SLK_MOUSE	0.99291245	0.64218009
SMAP2_MOUSE	0.91995148	0.04765165
SNAA_MOUSE	0.93173392	0.19459389
SNAB_MOUSE	0.97627099	0.49522152
SNAG_MOUSE	0.92826636	0.06734131
SND1_MOUSE	1.00211889	0.82368544
SNG1_MOUSE	0.93829235	0.08963179
SNG3_MOUSE	1.11486259	0.15167164
SNP25_MOUSE	0.8594587	0.05255507
SNP29_MOUSE	1.03890399	0.483857
SNP47_MOUSE	1.08553901	0.50412404
SNPH_MOUSE	0.94173791	0.14161218
SNX1_MOUSE	0.92577111	0.18448065
SNX12_MOUSE	1.00808218	0.85986006
SNX2_MOUSE	0.98818691	0.66347421
SNX27_MOUSE	0.95699162	0.27947091
SNX3_MOUSE	1.1333266	0.06478907
SNX30_MOUSE	2.21771871	0.0721492
SNX4_MOUSE	0.97005969	0.47716903
SNX5_MOUSE	1.02008195	0.81734289
SODM_MOUSE	1.08063166	0.477939
SOGA3_MOUSE	0.92122949	0.16849101

SORCN_MOUSE	0.94608544	0.14819572
SPB6_MOUSE	1.19075801	0.07879967
SPCS2_MOUSE	1.06168626	0.72908468
SPG7_MOUSE	1.0479023	0.50632023
SPN90_MOUSE	0.93930708	0.12426371
SPRE_MOUSE	0.97061794	0.33119798
SPRL1_MOUSE	1.0922769	0.94911159
SPRY4_MOUSE	0.91714582	0.18315361
SPTB1_MOUSE	0.94671099	0.29081609
SPTB2_MOUSE	0.99272696	0.68333013
SPTN1_MOUSE	0.98083127	0.5849659
SRBS1_MOUSE	0.98999119	0.61263145
SRBS2_MOUSE	1.05058638	0.56344619
SRC_MOUSE	0.99456918	0.64609853
SRC8_MOUSE	0.9315716	0.09193389
SRCN1_MOUSE	0.9731187	0.40206964
SRGP2_MOUSE	0.93186495	0.17842562
SRGP3_MOUSE	0.9410716	0.19740883
SRR_MOUSE	1.00273983	0.78911629
SSBP_MOUSE	1.02858033	0.96214633
SSDH_MOUSE	1.05336459	0.48457141
ST4A1_MOUSE	1.24981809	0.14885465
STAM1_MOUSE	0.96401984	0.28355118
STB5L_MOUSE	1.03537653	0.81945629
STIP1_MOUSE	0.90087173	0.02015739
STK24_MOUSE	1.09275702	0.50911679
STK39_MOUSE	0.9710908	0.43591398
STML2_MOUSE	1.03179651	0.87648485
STMN1_MOUSE	0.72510624	1.2867E-07
STRAP_MOUSE	0.94190064	0.36226366
STRN_MOUSE	0.94415223	0.23880527
STRN3_MOUSE	0.87127657	0.0786812
STRN4_MOUSE	0.94730094	0.27475718
STX12_MOUSE	0.90608217	0.18293903
STX1A_MOUSE	0.94308441	0.27267773
STX1B_MOUSE	0.92815793	0.21488608
STX7_MOUSE	0.96880581	0.47772908
STXB1_MOUSE	1.05753936	0.35767935
STXB3_MOUSE	0.893382	0.24011521

STXB5_MOUSE	0.99450876	0.71058841
SUCA_MOUSE	1.06217273	0.5478979
SUCB1_MOUSE	1.021981	0.77281985
SUCB2_MOUSE	1.05904375	0.26430523
SV2A_MOUSE	1.024776	0.81296736
SV2B_MOUSE	0.95634854	0.27412844
SVOP_MOUSE	0.94044311	0.25413697
SYAC_MOUSE	0.92403912	0.02220938
SYDC_MOUSE	1.04803505	0.79129583
SYEP_MOUSE	0.93075566	0.09574481
SYGP1_MOUSE	0.95533963	0.29042545
SYHC_MOUSE	0.90943028	0.07775275
SYIM_MOUSE	1.03859941	0.58928987
SYJ2B_MOUSE	0.98332061	0.5946796
SYK_MOUSE	1.33450634	0.61688277
SYLM_MOUSE	0.97177975	0.47096078
SYMM_MOUSE	1.09149155	0.16359392
SYN1_MOUSE	1.03637882	0.58926886
SYN2_MOUSE	1.0596935	0.64906204
SYN3_MOUSE	1.07163465	0.16762455
SYNC_MOUSE	0.94703939	0.26854893
SYNE1_MOUSE	0.92456657	0.072385
SYNJ1_MOUSE	1.02113633	0.85137508
SYNPO_MOUSE	0.99553727	0.84741596
SYNPR_MOUSE	0.96311067	0.36526735
SYPH_MOUSE	0.9845774	0.49400302
SYRC_MOUSE	1.0973343	0.20710731
SYSC_MOUSE	1.03284382	0.71494256
SYSM_MOUSE	1.04989415	0.71792161
SYT1_MOUSE	0.98533916	0.52971826
SYT12_MOUSE	0.92375458	0.03499333
SYT2_MOUSE	0.9861662	0.76967211
SYT3_MOUSE	0.98845584	0.73235031
SYT7_MOUSE	1.04949445	0.61120816
SYTC_MOUSE	1.00540656	0.84947119
SYTM_MOUSE	1.23043861	0.36645254
SYUA_MOUSE	0.85037165	0.01035041
SYUB_MOUSE	0.8592758	0.01747851
SYVC_MOUSE	0.94623633	0.45948154

SYWC_MOUSE	1.04217102	0.67100351
SYYM_MOUSE	1.13322252	0.05768868
T11L1_MOUSE	0.9461901	0.42538745
TACO1_MOUSE	1.06659555	0.68833384
TAGL3_MOUSE	1.01180859	0.85287693
TALDO_MOUSE	0.88202615	0.02335911
TANC2_MOUSE	0.94793374	0.33942391
TAU_MOUSE	0.92624884	0.22015272
TB10B_MOUSE	0.94753702	0.3484834
TBA4A_MOUSE	0.94867859	0.31322753
TBB3_MOUSE	1.09699947	0.6596472
TBB4A_MOUSE	0.90742209	0.16494622
TBB5_MOUSE	0.88218878	0.01988568
TBRG4_MOUSE	0.99649289	0.86518266
TCPA_MOUSE	0.98982054	0.69752925
TCPB_MOUSE	0.98585037	0.66673882
TCPD_MOUSE	1.00836233	0.88921639
TCPE_MOUSE	1.04022588	0.49950504
TCPG_MOUSE	0.99198598	0.72968721
TCPH_MOUSE	0.97792546	0.41557343
TCPQ_MOUSE	0.98445085	0.47690159
TCPR1_MOUSE	0.95944132	0.22282383
TCPZ_MOUSE	0.99504278	0.7495115
TCTP_MOUSE	0.89399001	0.16265678
TDRKH_MOUSE	0.96790514	0.37029702
TEBP_MOUSE	1.00688019	0.96098863
TECR_MOUSE	0.94458914	0.22841347
TENA_MOUSE	1.22311041	0.50258319
TENR_MOUSE	0.93551093	0.15694335
TERA_MOUSE	1.01049795	0.95930683
TFAM_MOUSE	0.92913588	0.26154215
TFR1_MOUSE	0.87528874	0.02695667
THEM4_MOUSE	1.00142319	0.85353249
THEM6_MOUSE	1.11408056	0.97465368
THIC_MOUSE	1.00423726	0.99748874
THIKA_MOUSE	1.04476178	0.44730613
THIL_MOUSE	0.98463867	0.55561864
THIM_MOUSE	1.02418541	0.91047678
THNS1_MOUSE	0.96415427	0.37261571

THOP1_MOUSE	0.90023	0.1180073
THTM_MOUSE	1.04462611	0.40216014
THTR_MOUSE	1.1169525	0.22702532
THY1_MOUSE	1.06909276	0.54064922
TIM10_MOUSE	0.96762335	0.56359936
TIM13_MOUSE	0.90944571	0.01948205
TIM29_MOUSE	1.11820605	0.06579198
TIM44_MOUSE	0.9690974	0.40315824
TIM50_MOUSE	0.94690242	0.05604746
TKT_MOUSE	0.9640878	0.26778813
TLN2_MOUSE	0.92267038	0.02838861
TM160_MOUSE	1.06120632	0.28164074
TM1L2_MOUSE	0.91096165	0.04149846
TMM11_MOUSE	0.99971579	0.9235086
TMM33_MOUSE	1.11270596	0.12955369
TMM65_MOUSE	0.99989303	0.76880802
TMOD2_MOUSE	1.00595735	0.93766663
TMX2_MOUSE	0.99040111	0.77287943
TMX3_MOUSE	1.00623572	0.95218588
TMX4_MOUSE	1.02786535	0.99878993
TNPO3_MOUSE	1.08265063	0.51795623
TOLIP_MOUSE	0.88994856	0.02700395
TOM1_MOUSE	1.0782633	0.30007052
TOM22_MOUSE	0.87148339	0.00639301
TOM40_MOUSE	0.92871867	0.31824035
TOM70_MOUSE	0.93413348	0.0500182
TP4A2_MOUSE	1.26450248	0.08853068
TPC_MOUSE	0.85941666	0.03571952
TPD52_MOUSE	0.88589387	0.05024765
TPD54_MOUSE	0.97256609	0.50168166
TPIS_MOUSE	0.96156479	0.30606936
TPM1_MOUSE	0.82158649	0.07685962
TPM3_MOUSE	0.83181788	0.12636424
TPP2_MOUSE	0.96435444	0.31314126
TPPP_MOUSE	0.98983512	0.74011714
TPPP3_MOUSE	0.87549819	0.00034584
TPRGL_MOUSE	1.00638025	0.93669913
TRAP1_MOUSE	1.0252592	0.79771354
TRFE_MOUSE	0.86161976	0.07740691

TRIM2_MOUSE	0.99869517	0.76358175
TRIM3_MOUSE	0.90037685	0.00616791
TRIO_MOUSE	0.98451337	0.57920402
TRPV2_MOUSE	1.06253942	0.75566225
TRXR1_MOUSE	1.182234	0.09563947
TRXR2_MOUSE	1.10992567	0.30197998
TS101_MOUSE	0.9987235	0.8092808
TSN_MOUSE	0.85806948	0.01495171
TSN2_MOUSE	0.78755327	0.01246341
TSN7_MOUSE	1.02855261	0.94388463
TSR2_MOUSE	0.78993627	0.01591154
TTC19_MOUSE	1.08804145	0.48822944
TTC7B_MOUSE	1.03321879	0.38739963
TTYH1_MOUSE	1.09244013	0.35325209
TTYH3_MOUSE	1.98762773	0.06407048
TWF1_MOUSE	0.96596414	0.3479013
TWF2_MOUSE	1.0414985	0.28373539
TXD12_MOUSE	0.90612918	0.21537386
TXND5_MOUSE	0.95008826	0.27725032
TXNL1_MOUSE	0.96188283	0.29792632
TXTP_MOUSE	0.99610194	0.83089066
TY3H_MOUSE	0.96575574	0.52756912
UB2L3_MOUSE	1.05690793	0.3724886
UB2V2_MOUSE	1.04748754	0.60753069
UBA1_MOUSE	0.95168029	0.28156258
UBA5_MOUSE	0.94309139	0.23308059
UBC12_MOUSE	0.95529765	0.14945501
UBE2N_MOUSE	0.99700888	0.79835596
UBE2O_MOUSE	0.99411847	0.84077818
UBP14_MOUSE	0.96358024	0.2858734
UBP5_MOUSE	0.97341633	0.38453958
UBP7_MOUSE	0.95512982	0.51509324
UBQL1_MOUSE	0.91287205	0.16683312
UBQL2_MOUSE	0.96792371	0.37771157
UBR4_MOUSE	1.07291953	0.30335954
UBXN6_MOUSE	1.09404209	0.43693386
UCHL1_MOUSE	0.94324162	0.13856474
UCHL3_MOUSE	0.97188485	0.48368161
UCRI_MOUSE	1.11489258	0.08478631

UGPA_MOUSE	1.0308431	0.88989633
ULA1_MOUSE	0.98113754	0.54432128
UN13A_MOUSE	1.01711656	0.60763339
UQCC1_MOUSE	0.92407424	0.02441843
USMG5_MOUSE	1.02403423	0.93235496
USO1_MOUSE	0.95990583	0.34881409
USP9X_MOUSE	0.9799593	0.40949594
VA0D1_MOUSE	1.024633	0.77378582
VAC14_MOUSE	1.11176354	0.31734126
VAMP1_MOUSE	0.89368993	0.07889834
VAMP2_MOUSE	0.87252571	0.08498467
VAPA_MOUSE	1.14396064	0.08326456
VAPB_MOUSE	0.97259054	0.42178386
VAS1_MOUSE	1.0109867	0.93260932
VAT1_MOUSE	1.12942398	0.02496447
VAT1L_MOUSE	1.08616339	0.0217056
VATA_MOUSE	0.97969198	0.53996233
VATB2_MOUSE	1.03428276	0.63523004
VATC1_MOUSE	1.01698142	0.7358551
VATD_MOUSE	1.01549774	0.91317265
VATE1_MOUSE	0.9533422	0.18390816
VATF_MOUSE	0.975514	0.31551618
VATG1_MOUSE	0.91381164	0.12155224
VATG2_MOUSE	0.92537756	0.12362507
VATH_MOUSE	1.04035333	0.51400887
VATL_MOUSE	1.43817644	0.0089681
VCAM1_MOUSE	1.1060851	0.154121
VCIP1_MOUSE	0.89452199	0.02421264
VDAC1_MOUSE	0.96794906	0.35573662
VDAC2_MOUSE	0.94635447	0.05771806
VDAC3_MOUSE	0.96134227	0.23916539
VGLU1_MOUSE	1.12942693	0.27134059
VGLU2_MOUSE	0.95270776	0.29404936
VIAAT_MOUSE	1.08785407	0.19056714
VIME_MOUSE	0.82043361	0.00221274
VINC_MOUSE	1.00728389	0.9974669
VISL1_MOUSE	0.91172632	0.03859952
VMA5A_MOUSE	1.04581816	0.84178722
VP13A_MOUSE	1.04297438	0.61735275

VP13C_MOUSE	0.92050447	0.236069
VP26A_MOUSE	1.01525119	0.95772505
VP26B_MOUSE	1.01425214	0.84112908
VPP1_MOUSE	0.99153302	0.66960457
VPS16_MOUSE	1.10985587	0.3395319
VPS29_MOUSE	1.07236188	0.55709807
VPS35_MOUSE	0.99336806	0.67153648
VPS45_MOUSE	1.07015371	0.0585805
VPS50_MOUSE	1.02366727	0.78410892
VPS51_MOUSE	1.03896638	0.61273325
VPS53_MOUSE	1.20042907	0.18981004
VTA1_MOUSE	0.92807145	0.03330006
VWA8_MOUSE	1.01780504	0.90252564
WASF1_MOUSE	0.96679698	0.28621079
WASF3_MOUSE	0.93731895	0.41155407
WASL_MOUSE	1.24999272	0.0501783
WBP2_MOUSE	1.0204201	0.9553125
WDFY3_MOUSE	1.10767713	0.26354883
WDR1_MOUSE	1.03237583	0.56281059
WDR37_MOUSE	1.05139273	0.3096423
WDR44_MOUSE	0.87542043	0.18360738
WDR47_MOUSE	1.00684118	0.93506322
WDR48_MOUSE	1.01385339	0.85738402
WDR7_MOUSE	0.98605399	0.64379707
WFS1_MOUSE	1.01617449	0.87820962
WIPI2_MOUSE	1.03539309	0.4650419
WNK2_MOUSE	1.12406668	0.17475338
XPO1_MOUSE	1.07081961	0.58406158
XPO2_MOUSE	0.99901198	0.81706332
XPO7_MOUSE	1.06797572	0.83036433
XPP1_MOUSE	0.92329385	0.03712718
YKT6_MOUSE	0.96367688	0.32493949
ZC21A_MOUSE	0.97277742	0.48864905
ZNT3_MOUSE	0.9808544	0.46353926
ZNT9_MOUSE	1.02329844	0.64256207
ZO1_MOUSE	1.11821229	0.04610989
ZO2_MOUSE	0.88341097	0.02445254
