# Supplementary material for: Identification of substrates of palmitoyl protein thioesterase 1 highlights roles of depalmitoylation in disulfide bond formation and synaptic function
Source: PLoS Biol. 2022 Mar 31;20(3):e3001590. doi: 10.1371/journal.pbio.3001590 (PMC9004782; doi:10.1371/journal.pbio.3001590)
Supplement: S2 Table — These data correspond to Fig 2B and are the expression ratios for proteins that exhibit significant changes in the palmitome (n = 242). Proteins marked with an asterisk (*) were removed from subsequent consideration as PPT1 substrates due to decreased palmitoylation (n = 4: HDHD2, S1PR1, SNX1, UBQL2; blue <1.5-fold change), presence in the CRAPome [97] (n = 9: 1433E, COF1, HNRPU, KPYM, RL13, RL22, RL4, RLA0, TCPD), lack of a cysteine residue (n = 2: NDUA6, SYUA), increased palmitoylation and protein expression (n = 4: ASAH1, CATD, SCRB2, and TPP1), or lack of detection in the synaptic proteome (n = 19: CKAP4, CPNE1, FHL1, GPC5B, ITM2B, ITM2C, LGI2, MAGI1, NSMA2, PP1G, PRDX4, R7BP, RB3GP, S39AC, TPPC3, VAMP7, XKR4, S1PR1, MYO6, PDPR; removed proteins S1PR1, HNRPU, and RL22 also fall in this category). The remaining 204 proteins are the final list of putative PPT1 substrates prior to the validation screen. p-Values were calculated using a 2-tailed t test. Red denotes significantly increased expression in the palmitome (>1.5-fold). KO, knockout; PPT1, palmitoyl protein thioesterase 1; WT, wild-type. (PDF) [file pbio.3001590.s003.pdf]

S2 Table. Proteins exhibiting significant changes in WT versus PPT1 KO synaptic palmitome. These data correspond to **Figure 2B** and are the expression ratios for proteins that exhibit significant changes in the palmitome (n = 242). Proteins marked with an asterisk (*) were removed from subsequent consideration as PPT1 substrates due to decreased palmitoylation (n = 4: HDHD2, S1PR1, SNX1, UBQL2 ; blue <1.5-fold change), presence in the CRAPome [97] (n = 9: 1433E, COF1, HNRPU, KPYM, RL13, RL22, RL4, RLA0, TCPD), lack of a cysteine residue (n = 2: NDUA6, SYUA), increased palmitoylation and protein expression (n = 4: ASAH1, CATD, SCRB2, and TPP1), or lack of detection in the synaptic proteome (n = 19: CKAP4, CPNE1, FHL1, GPC5B, ITM2B, ITM2C, LGI2, MAGI1, NSMA2, PP1G, PRDX4, R7BP, RB3GP, S39AC, TPPC3, VAMP7, XKR4, S1PR1, MYO6, PDPR; removed proteins S1PR1, HNRPU, and RL22 also fall in this category). The remaining 204 proteins are the final list of putative PPT1 substrates prior to the validation screen. P-values were calculated using a two-tailed t-test. Red denotes significantly increased expression in the palmitome (>1.5-fold).

UniProt ID	Average Ratio (KO/WT)	P value
HDHD2_MOUSE*	0.65809308	0.02504326
S1PR1_MOUSE*	0.43333627	0.01690137
SNX1_MOUSE*	0.55468914	0.0240519
UBQL2_MOUSE*	0.57153189	0.01479205
1433B_MOUSE	6.16513858	0.03447738
1433E_MOUSE*	1.7373229	0.04223438
6PGL_MOUSE	37.011627	0.04825557
ABHGA_MOUSE	1.73812986	0.02325394
ABR_MOUSE	2.06555242	0.0190207
ACON_MOUSE	2.37614763	0.0264551
ACSF2_MOUSE	6.85911933	0.04305497
ACTN1_MOUSE	2.94150451	0.00907073
ADDG_MOUSE	2.02630442	0.0064112
AIFM1_MOUSE	2.81649658	0.04296289
AL1L1_MOUSE	3.12190544	0.01219849
AL3A2_MOUSE	2.23955338	0.02893705
ANK3_MOUSE	2.44174507	0.02532274
AP2A1_MOUSE	1.65944151	0.02989611
AP2B1_MOUSE	2.39035809	0.02267046
AP2M1_MOUSE	2.50176909	0.04655494
ASAH1_MOUSE*	5.01610876	0.00167502
ASTN1_MOUSE	1.66472933	0.01603359
AT1A1_MOUSE	1.83121203	0.00714207
AT1A2_MOUSE	1.74425582	0.0026334
AT1A3_MOUSE	1.74595803	0.02985501
AT1B1_MOUSE	2.17994482	0.01875778
AT1B2_MOUSE	6.85159323	0.01769292

AT2B4_MOUSE	1.73884867	0.01144252
ATAD3_MOUSE	1.77376102	0.03722607
ATP5E_MOUSE	2.93731529	0.03721463
ATPG_MOUSE	3.39113512	0.03156482
ATPO_MOUSE	6.13811283	0.04397278
BAIP2_MOUSE	2.59538287	0.04601941
BASI_MOUSE	2.69458393	0.0156804
BRSK2_MOUSE	1.84487865	0.01505495
CA2D1_MOUSE	3.16785152	0.01823684
CAC1E_MOUSE	2.08654395	0.00724175
CADM2_MOUSE	1.97486452	0.00348435
CAPS1_MOUSE	2.47981609	0.04828016
CATD_MOUSE*	8.6144266	0.00377278
CBPE_MOUSE	3.12398966	0.04667749
CCG8_MOUSE	3.17187285	0.02036904
CCNY_MOUSE	3.50274587	0.01081474
CD47_MOUSE	2.98286693	0.00840764
CD81_MOUSE	2.55345522	0.04181171
CISD1_MOUSE	1.91992184	0.0499723
CISY_MOUSE	2.97152825	0.02582269
CKAP4_MOUSE*	1.8302586	0.03220663
CLH1_MOUSE	2.80442701	0.02324201
CLUS_MOUSE	7.87342416	0.02781312
CNDP2_MOUSE	1.57796766	0.03215849
COF1_MOUSE*	3.70220246	0.02701788
COTL1_MOUSE	2.24339821	0.02674421
COX2_MOUSE	1.66755743	0.04724754
COX41_MOUSE	8.04690807	0.02848797
CPNE1_MOUSE*	3.37323728	0.04085177
CRIP2_MOUSE	6.82769925	0.04955228
CSPG2_MOUSE	6.24611017	0.04521241
CTNA2_MOUSE	1.87486094	0.04632975
CXA1_MOUSE	2.91852558	0.03654657
CYFP1_MOUSE	11.9840583	0.04398267
DCE1_MOUSE	2.84288313	0.04296491
DCE2_MOUSE	1.87714304	0.0499237
DCLK1_MOUSE	2.62481528	0.02118323
DCTN1_MOUSE	1.56010131	0.01730595
DDB1_MOUSE	2.72411734	0.00475071

DEST_MOUSE	4.34731089	0.02196256
DHB4_MOUSE	1.69748231	0.04491371
DIP2B_MOUSE	1.96580728	0.03076635
DMXL2_MOUSE	1.63516189	0.03312546
DNJC5_MOUSE	5.11996889	0.01085887
DPP6_MOUSE	1.5301643	0.01851725
DYL2_MOUSE	4.19224278	0.02707414
DYN1_MOUSE	2.31587103	0.04280089
EFTU_MOUSE	1.52726819	0.00581057
ENTP2_MOUSE	2.19407876	0.00625933
EP15R_MOUSE	1.80511174	0.04214984
EPHA4_MOUSE	2460.83085	0.01654109
ERP29_MOUSE	17.3706319	0.02648587
F10A1_MOUSE	2.09881456	0.03346201
FA49B_MOUSE	2.07208701	0.0411204
FAS_MOUSE	2.07741976	0.01219864
FBX41_MOUSE	2.70989241	0.0363276
FHL1_MOUSE*	3.34333736	0.03506652
FUMH_MOUSE	2.42120508	0.04220973
GABR1_MOUSE	3.28428604	0.01480859
GABR2_MOUSE	3.43425887	0.03273567
GBB2_MOUSE	2.96271577	0.00257017
GBRG2_MOUSE	8.89414293	0.03526663
GNA13_MOUSE	2.49947339	0.00290725
GNAI1_MOUSE	3.3179847	0.02874637
GNAI2_MOUSE	4.67638206	0.03740986
GNAO_MOUSE	4.80553608	0.02535557
GNAQ_MOUSE	3.93664545	0.03285901
GNAZ_MOUSE	2.89572628	0.02645697
GPC5B_MOUSE*	4.4881951	0.02189772
GPDA_MOUSE	2.07136789	0.04772962
GPM6A_MOUSE	5.62523104	0.00678947
GRIA1_MOUSE	1.86606467	0.04587879
GRIA2_MOUSE	2.00334698	0.03965339
GRP78_MOUSE	1.71353245	0.00515339
GTR1_MOUSE	2.82450062	0.04301984
HCN1_MOUSE	1.84696478	0.03383597
HEXB_MOUSE	2.25030845	0.04017271
HNRPU_MOUSE*	3.08409018	0.03077163

HS12A_MOUSE	2.02599041	0.04575905
HSP74_MOUSE	2.09076683	0.02485589
HXK1_MOUSE	1.86417568	0.03223978
IMPA1_MOUSE	3.86612134	0.0392026
ITM2B_MOUSE*	7.39999446	0.00713478
ITM2C_MOUSE*	4.82790884	0.0202228
ITSN1_MOUSE	2.93919762	0.02534814
IVD_MOUSE	1.62426359	0.0289076
KCC2D_MOUSE	1.56455433	0.01699244
KCND2_MOUSE	3.5143829	0.00905985
KCRB_MOUSE	1.81458902	0.02929205
KI21A_MOUSE	1.84691714	0.0114136
KIF5C_MOUSE	1.66905138	0.03508453
KPYM_MOUSE*	2.01532364	0.01438118
KTN1_MOUSE	2.7855293	0.01301305
LAT1_MOUSE	4.00246603	0.03615308
LDHB_MOUSE	1.92755339	0.04998723
LETM1_MOUSE	1.85597866	0.04277988
LGI1_MOUSE	2.04735999	0.01299686
LGI2_MOUSE*	4.74635604	0.04678316
LIPA3_MOUSE	1.68592935	0.0239067
LIS1_MOUSE	2.0801692	0.02626258
LRRC7_MOUSE	1.93307508	5.4308E-05
M2OM_MOUSE	3.10655303	0.03588682
MADD_MOUSE	2.7802968	0.02157297
MAGI1_MOUSE*	2.71135344	0.00303765
MBLC2_MOUSE	7.94560385	0.01830526
MIA40_MOUSE	8.28682583	0.04383079
MPP2_MOUSE	2.37879293	0.02489248
MPP6_MOUSE	1.96903366	0.00068579
MRCKB_MOUSE	3.37056361	0.00453073
MYO6_MOUSE*	7.65775067	0.04038318
NAC1_MOUSE	1.60781165	0.0205655
NCALD_MOUSE	45.0986498	0.03333189
NCKP1_MOUSE	1.76101097	0.03686968
NDKA_MOUSE	5.18466229	0.01994241
NDUA6_MOUSE*	3.22566946	0.04169493
NDUS1_MOUSE	1.85921323	0.00543366
NF1_MOUSE	4.40251115	0.02493713

NFASC_MOUSE	1.52392687	0.03292294
NNTM_MOUSE*	5.15295413	0.00064755
NRCAM_MOUSE	1.71358651	0.01137874
NRX3A_MOUSE	2.86290478	0.02537298
NSF_MOUSE	1.79680977	0.01105155
NSMA2_MOUSE*	3.0656357	0.01513815
NTRI_MOUSE	4.06026601	0.03255085
OAT_MOUSE	2.38337287	0.04077862
ODO1_MOUSE	2.73375853	0.0474292
ODPX_MOUSE	1.55516198	0.01906577
OPA1_MOUSE	1.90523472	0.04247727
OXR1_MOUSE	1.65508292	0.00273805
PALM2_MOUSE	4.02528407	0.01746552
PCCA_MOUSE	1.52423112	0.03218611
PDE2A_MOUSE	1.70966649	0.01001083
PDIA4_MOUSE	3.02013745	0.0411904
PDPR_MOUSE*	2.17601378	0.00014154
PGM1_MOUSE	1.97753773	0.00126391
PI4KA_MOUSE	2.83055274	0.0147451
PLCB1_MOUSE	1.99490751	0.01982732
PLPR4_MOUSE	2.50426577	0.01188047
PNPT1_MOUSE	47.0149712	0.03136547
PP1G_MOUSE*	4.42574217	0.04759373
PP2BA_MOUSE	1.76925852	0.02415911
PRDX4_MOUSE*	4.45364429	0.0123701
PRDX6_MOUSE	1.6130104	0.0298685
PRRT3_MOUSE	2.28770195	0.02904083
PTPRD_MOUSE	2.86420019	0.03072229
PTPRS_MOUSE	2.25908928	0.02607941
PYGB_MOUSE	3.99445536	0.03208737
R7BP_MOUSE*	12.474933	0.04004716
RAB1A_MOUSE	3.9013582	0.04863898
RAB3B_MOUSE	11.741844	0.01679947
RAB7A_MOUSE	1.84884089	0.03543951
RAN_MOUSE	2.49778976	0.02187895
RAP2B_MOUSE	8.62180744	0.02144014
RASH_MOUSE	3.37978611	0.01763677
RB3GP_MOUSE*	4.54828995	0.01411416
RBGPR_MOUSE	3.97162065	0.03226824

RHOG_MOUSE	1.93802777	0.02717621
RL13_MOUSE*	4.26738647	0.01404968
RL22_MOUSE*	9.99948728	0.03805332
RL4_MOUSE*	2.71230165	0.03870093
RL5_MOUSE	2.24191057	0.0344874
RLA0_MOUSE*	8.16503281	0.03251642
RMD3_MOUSE	38.0510859	0.04528451
RPGF4_MOUSE	2.68455757	0.02402321
RPN1_MOUSE	4.6181334	0.01553258
S12A2_MOUSE	2.59519204	0.03958399
S39AC_MOUSE*	2.75310155	0.04764758
SAHH2_MOUSE	2.71243878	0.01356875
SATT_MOUSE	8.42850683	0.04403561
SC6A1_MOUSE	3.41449618	0.04661652
SCAM1_MOUSE	3.27589443	0.03360363
SCN1A_MOUSE	6.14798148	0.01534375
SCRB2_MOUSE*	3.72143443	0.00760874
SDHB_MOUSE	2.84421004	0.02049879
SEPT8_MOUSE	1.78476244	0.0198599
SFXN1_MOUSE	7.93326104	0.04248025
SFXN3_MOUSE	2.37253319	0.01622603
SND1_MOUSE	2.10071498	0.03792139
SPB6_MOUSE	2.54675778	0.02905788
SPRL1_MOUSE	1.70498504	0.03331293
SRBS2_MOUSE	2.21686344	0.04168358
SSDH_MOUSE	1.66631376	0.00500585
STXB1_MOUSE	1.75202274	0.03577848
SUCB2_MOUSE	3.5185261	0.04360612
SYIM_MOUSE	1.85343949	0.03479621
SYNJ1_MOUSE	2.17892072	0.02888376
SYNPR_MOUSE	6.44464358	0.01783558
SYT2_MOUSE	2.48418822	0.04940637
SYT7_MOUSE	2.72888773	0.01288555
SYUA_MOUSE*	8.72755643	0.04972285
TCPD_MOUSE*	1.7520083	0.01304949
TCPH_MOUSE	2.26558686	0.02937783
TERA_MOUSE	2.18260205	0.02571782
THIKA_MOUSE	13.1940085	0.03926997
THY1_MOUSE	3.3568611	0.02204422

TIM13_MOUSE	3.78323177	0.02091121
TPP1_MOUSE*	3.44625467	0.00473966
TPPC3_MOUSE*	2.46087779	0.01246953
UBA1_MOUSE	2.30346159	0.0187566
UBE2O_MOUSE	2.14510163	0.03154608
UCRI_MOUSE	4.83957182	0.0492247
USMG5_MOUSE	2.37515292	0.03253737
VA0D1_MOUSE	4.63836049	0.03338637
VAMP1_MOUSE	6.08333941	0.01721828
VAMP7_MOUSE*	15.422196	0.03328643
VATE1_MOUSE	2.81657735	0.03904979
VCIP1_MOUSE	4.69443448	0.03133417
VDAC2_MOUSE	2.92874791	0.0286335
VGLU2_MOUSE	1.96685604	0.00333991
VISL1_MOUSE	2.45048957	0.03958976
VPS29_MOUSE	25.4882128	0.04026089
VPS35_MOUSE	2.28353437	0.01015621
VTA1_MOUSE	39.3736025	0.04203121
WFS1_MOUSE	5.30342783	0.04325322
XKR4_MOUSE*	3.20447199	0.01798265
