# Supplementary material for: Identification of substrates of palmitoyl protein thioesterase 1 highlights roles of depalmitoylation in disulfide bond formation and synaptic function
Source: PLoS Biol. 2022 Mar 31;20(3):e3001590. doi: 10.1371/journal.pbio.3001590 (PMC9004782; doi:10.1371/journal.pbio.3001590)
Supplement: S3 Table — [x] Carbamidomethyl indicates location of palmitoylated cysteine (C) in peptide sequence. PPT1, palmitoyl protein thioesterase 1. (PDF) [file pbio.3001590.s004.pdf]

S3 Table. Formerly palmitoylated peptides identified for putative PPT1 substrates. [x] Carbamidomethyl indicates location of palmitoylated cysteine (C) in peptide sequence.

**Protein Name (Uniprot ID)**	**Peptide Sequence**	**Modifications**
ACON_MOUSE	CTTDHISAAGPWLK	[1] Carbamidomethyl
ACON_MOUSE	DVGGIVLANACGPCIGQWDRK	[11] Carbamidomethyl |[14] Carbamidomethyl
ACON_MOUSE	VAVPSTIHCDHLIEAQVGGEK	[9] Carbamidomethyl
ACON_MOUSE	VGLIGSCTNSSYEDMGR	[7] Carbamidomethyl
ACTN1_MOUSE	DGLGFCALIHR	[6] Carbamidomethyl
ACTN1_MOUSE	EGLLLWCQR	[7] Carbamidomethyl
AL1L1_MOUSE	ECDVLPDDTVSTLYNR	[2] Carbamidomethyl
AL1L1_MOUSE	IAVIGQSLFGQEVYCQLR	[15] Carbamidomethyl
AL1L1_MOUSE	LRGEDGESECVINYVEK	[10] Carbamidomethyl
AL1L1_MOUSE	SPLIIFADCDLNK	[9] Carbamidomethyl
AL1L1_MOUSE	VPGAWTEACGQK	[9] Carbamidomethyl
ANK3_MOUSE	DIEVLEGKPIYVDCYGNLAPLTK	[14] Carbamidomethyl
AP2A1_MOUSE	AADLLYAMCDR	[9] Carbamidomethyl
AP2A1_MOUSE	ACNQLGQFLQHR	[2] Carbamidomethyl
AP2A1_MOUSE	ALQVGCLLR	[6] Carbamidomethyl
AP2A1_MOUSE	HLCELLAQQF	[3] Carbamidomethyl
AP2A1_MOUSE	QSAALCLLR	[6] Carbamidomethyl
AP2A1_MOUSE	TVFEALQAPACHENMVK	[11] Carbamidomethyl
AP2A1_MOUSE	YLALESMCTLASSEFSHEAVK	[8] Carbamidomethyl
AP2B1_MOUSE	DVSSLFPDVVNCMQTDNLELK	[12] Carbamidomethyl
AP2M1_MOUSE	IPTPLNTSGVQVICMK	[14] Carbamidomethyl
AP2M1_MOUSE	MCDVMAAYFGK	[2] Carbamidomethyl
AP2M1_MOUSE	QSIAIDDCTFHQCVR	[8] Carbamidomethyl |[13] Carbamidomethyl
AP2M1_MOUSE	SYLSGMPECK	[9] Carbamidomethyl

ASTN1_MOUSE	TLDSLQGCNEK	[8] Carbamidomethyl
AT1A1_MOUSE	LIIVEGCQR	[7] Carbamidomethyl
AT1A1_MOUSE	NIAFFSTNCVEGTAR	[9] Carbamidomethyl
AT1A1_MOUSE	NLEAVETLGSTSTICSDK	[15] Carbamidomethyl
AT1A2_MOUSE	CIELSCGSVR	[1] Carbamidomethyl |[6] Carbamidomethyl
AT1A2_MOUSE	CSTILVQGK	[1] Carbamidomethyl
AT1A2_MOUSE	LCFVGLMSMIDPPR	[2] Carbamidomethyl
AT1A2_MOUSE	LIIVEGCQR	[7] Carbamidomethyl
AT1A2_MOUSE	NICFFSTNCVEGTAR	[3] Carbamidomethyl |[9] Carbamidomethyl
AT1A2_MOUSE	NLEAVETLGSTSTICSDK	[15] Carbamidomethyl
AT1A2_MOUSE	VLGFCQLNLPSGKFPR	[5] Carbamidomethyl
AT1A3_MOUSE	ACVIHGTDLK	[2] Carbamidomethyl
AT1A3_MOUSE	CATILLQGK	[1] Carbamidomethyl
AT1A3_MOUSE	CIELSSGSVK	[1] Carbamidomethyl
AT1A3_MOUSE	KYNTDCVQGLTHSK	[6] Carbamidomethyl
AT1A3_MOUSE	LIIVEGCQR	[7] Carbamidomethyl
AT1A3_MOUSE	MSVEEVCRK	[7] Carbamidomethyl
AT1A3_MOUSE	NITFFSTNCVEGTAR	[9] Carbamidomethyl
AT1A3_MOUSE	NLEAVETLGSTSTICSDK	[15] Carbamidomethyl
AT1A3_MOUSE	SPDCTHDNPLETR	[4] Carbamidomethyl
AT1A3_MOUSE	SSHTWVALSHIAGLCNR	[15] Carbamidomethyl
AT1A3_MOUSE	VLGFCHYYLPEEQFPK	[5] Carbamidomethyl
AT1A3_MOUSE	YNTDCVQGLTHSK	[5] Carbamidomethyl
AT1B1_MOUSE	DDMIFEDCGNVPSEPK	[8] Carbamidomethyl
AT1B1_MOUSE	DSAQKDDMIFEDCGNVPSEPK	[13] Carbamidomethyl
AT1B1_MOUSE	YNPNVLPVQCTGK	[10] Carbamidomethyl
AT1B2_MOUSE	FLNVTPNVEVNVECR	[14] Carbamidomethyl
AT1B2_MOUSE	KSCGQVVEEWK	[3] Carbamidomethyl

AT1B2_MOUSE	SCGQVVEEWK	[2] Carbamidomethyl
AT1B2_MOUSE	TQLGDCSGIGDPTHYGYSTGQPCVFIK	[6] Carbamidomethyl |[23] Carbamidomethyl
AT2B4_MOUSE	GADAVAQISAHYGGVQEICTR	[19] Carbamidomethyl
AT2B4_MOUSE	HLDACETMGNATAICSDK	[5] Carbamidomethyl |[15] Carbamidomethyl
AT2B4_MOUSE	TECGLLGFVTDLK	[3] Carbamidomethyl
ATPG_MOUSE	GLCGAIHSSVAK	[3] Carbamidomethyl
ATPO_MOUSE	GEVPCTVTTASPLDDAVLSELK	[5] Carbamidomethyl
CADM2_MOUSE	IIPSTPFPQEGQALTLTCESK	[18] Carbamidomethyl
CAPS1_MOUSE	HGMDEFISSNPCNFDHASLFEMVQR	[12] Carbamidomethyl
CAPS1_MOUSE	IVYCTMEVEGGEK	[4] Carbamidomethyl
CAPS1_MOUSE	KFEHQLLYNACQLDNPDEQAAQIR	[11] Carbamidomethyl
CAPS1_MOUSE	LCSMEMGQEHQYHSK	[2] Carbamidomethyl
CAPS1_MOUSE	LMASDMIESCVK	[10] Carbamidomethyl
CATD_MOUSE	AIGAVPLIQGEYMIPCEK	[16] Carbamidomethyl
CATD_MOUSE	GGCEAIVDTGTSLLVGPVEEVK	[3] Carbamidomethyl
CATD_MOUSE	ILDIACWVHHK	[6] Carbamidomethyl
CBPE_MOUSE	SGTAHEYSSCPDDAIFQSLAR	[10] Carbamidomethyl
CD81_MOUSE	NSLCPSGGNILTPLLQQDCHQK	[4] Carbamidomethyl |[19] Carbamidomethyl
CD81_MOUSE	TFHETLNCCGSNALTTLTTTILR	[8] Carbamidomethyl |[9] Carbamidomethyl
CISD1_MOUSE	KFPFCDGAHIK	[5] Carbamidomethyl
CISY_MOUSE	GYSIPECQK	[7] Carbamidomethyl
CLH1_MOUSE	CNEPAVWSQLAK	[1] Carbamidomethyl
CLH1_MOUSE	EDKLECSEELGDLVK	[6] Carbamidomethyl
CLH1_MOUSE	EVCFACVDGK	[3] Carbamidomethyl |[6] Carbamidomethyl
CLH1_MOUSE	EVCFACVDGK	[3] Nethylmaleimide+water |[6] Carbamidomethyl
CLH1_MOUSE	EVCFACVDGK	[3] Carbamidomethyl |[6] Nethylmaleimide+water
CLH1_MOUSE	GQCDLELINVCNENSLFK	[3] Carbamidomethyl |[11] Carbamidomethyl
CLH1_MOUSE	HSSLAGCQIINYR	[7] Carbamidomethyl

CLH1_MOUSE	IHEGCEEPATHNALAK	[5] Carbamidomethyl
CLH1_MOUSE	LECSEELGDLVK	[3] Carbamidomethyl
CLH1_MOUSE	RDPHLACVAYER	[7] Carbamidomethyl
CLH1_MOUSE	VIQCFAETGQVQK	[4] Carbamidomethyl
CLH1_MOUSE	YESLELCRPVLQQGR	[7] Carbamidomethyl
CRIP2_MOUSE	ASSVTTFTGEPNMCPR	[14] Carbamidomethyl
CSPG2_MOUSE	FTFEEAEAECTSR	[10] Carbamidomethyl
CTNA2_MOUSE	LESIISGAALMADSSCTR	[16] Carbamidomethyl
CYFP1_MOUSE	DCPDNAEEYER	[2] Carbamidomethyl
DCE1_MOUSE	NLLSCENSDQGAR	[5] Carbamidomethyl
DCE2_MOUSE	LCALLYGDSGKPAEGGGSVTSR	[2] Carbamidomethyl
DCE2_MOUSE	MMGVPLQCSALLVR	[8] Carbamidomethyl
DCTN1_MOUSE	QSCTILISTMNK	[3] Carbamidomethyl
DMXL2_MOUSE	EIAALHEICNHESVIK	[9] Carbamidomethyl
DMXL2_MOUSE	VGCPVLALEVLSK	[3] Carbamidomethyl
DMXL2_MOUSE	VINLSQYGPACFGQEHAR	[11] Carbamidomethyl
DPP6_MOUSE	GENQGQTFTCGSALSPITDFK	[10] Carbamidomethyl
DYL2_MOUSE	KYNPTWHCIVGR	[8] Carbamidomethyl
DYL2_MOUSE	NADMSEDMQQDAVDCATQAMEK	[15] Carbamidomethyl
DYN1_MOUSE	ENCLILAVSPANSDLANSDALK	[3] Carbamidomethyl
DYN1_MOUSE	QLELACETQEEVDSWK	[6] Carbamidomethyl
ENTP2_MOUSE	AGQSLVECLEQALR	[8] Carbamidomethyl
FAS_MOUSE	ACVDTALENLSTLK	[2] Carbamidomethyl
FAS_MOUSE	FVFTPHMEAECLSESTALQK	[11] Carbamidomethyl
FAS_MOUSE	GYDYGPQFQGICEATLEGEQGK	[12] Carbamidomethyl
FAS_MOUSE	LGMLSPDGTCR	[10] Carbamidomethyl
FAS_MOUSE	SSCTIIPLMK	[3] Carbamidomethyl
GABR2_MOUSE	VFCCAFEESMFGSK	[3] Carbamidomethyl |[4] Carbamidomethyl

GBB2_MOUSE	ACGDSTLTQITAGLDPVGR	[2] Carbamidomethyl
GBB2_MOUSE	ELPGHTGYLSCCR	[11] Carbamidomethyl |[12] Carbamidomethyl
GBB2_MOUSE	KACGDSTLTQITAGLDPVGR	[3] Carbamidomethyl
GBB2_MOUSE	TFVSGACDASIK	[7] Carbamidomethyl
GBB2_MOUSE	VSCLGVTDDGMAVATGSWDSFLK	[3] Carbamidomethyl
GBRG2_MOUSE	DCASFFCCFEDCR	[2] Carbamidomethyl |[7] Carbamidomethyl |[8] Carbamidomethyl |[12] Carbamidomethyl
GNAI1_MOUSE	DSGVQACFNR	[7] Carbamidomethyl
GNAI1_MOUSE	EIYTHFTCATDTK	[8] Carbamidomethyl
GNAI1_MOUSE	IIHEAGYSEEECK	[12] Carbamidomethyl
GNAI1_MOUSE	LFDSICNNK	[6] Carbamidomethyl
GNAI2_MOUSE	EIYTHFTCATDTK	[8] Carbamidomethyl
GNAI2_MOUSE	IIHEDGYSEEECR	[12] Carbamidomethyl
GNAI2_MOUSE	LFDSICNNK	[6] Carbamidomethyl
GNAI2_MOUSE	LWADHGVQACFGR	[10] Carbamidomethyl
GNAI2_MOUSE	RLWADHGVQACFGR	[11] Carbamidomethyl
GNAO_MOUSE	LWGDSGIQECFNR	[10] Carbamidomethyl
GNAO_MOUSE	MHESLMLFDSICNNK	[12] Carbamidomethyl
GNAO_MOUSE	MVCDVVSR	[3] Carbamidomethyl
GNAQ_MOUSE	IIYSHFTCATDTENIR	[8] Carbamidomethyl
GNAQ_MOUSE	SLWNDPGIQECYDR	[11] Carbamidomethyl
GNAQ_MOUSE	TLESIMACCLSEEAK	[8] Carbamidomethyl |[9] Carbamidomethyl
GNAZ_MOUSE	LWADPGAQACFGR	[10] Carbamidomethyl
GPM6A_MOUSE	ICTASENFLR	[2] Carbamidomethyl
GPM6A_MOUSE	KICTASENFLR	[3] Carbamidomethyl
GRIA1_MOUSE	GFCLIPQQSINEAIR	[3] Carbamidomethyl
GRIA1_MOUSE	RGNAGDCLANPAVPWGQGIDIQR	[7] Carbamidomethyl
GRIA2_MOUSE	RGNAGDCLANPAVPWGQGVEIER	[7] Carbamidomethyl
HS12A_MOUSE	EPECIHVMR	[4] Carbamidomethyl

HSP74_MOUSE	GCALQCAILSPAFK	[2] Carbamidomethyl |[6] Carbamidomethyl
HSP74_MOUSE	SVMDATQIAGLNCLR	[13] Carbamidomethyl
HXK1_MOUSE	AAQLCGAGMAAVVEK	[5] Carbamidomethyl
HXK1_MOUSE	AILQQLGLNSTCDDSILVK	[12] Carbamidomethyl
HXK1_MOUSE	ATDCVGHDVATLLR	[4] Carbamidomethyl
HXK1_MOUSE	KLPVGFTFSFPCR	[12] Carbamidomethyl
HXK1_MOUSE	MPLGFTFSFPCK	[11] Carbamidomethyl
IMPA1_MOUSE	YPCHSFIGEESVAAGEK	[3] Carbamidomethyl
KCRB_MOUSE	FCTGLTQIETLFK	[2] Carbamidomethyl
KI21A_MOUSE	TVNTEPEMMQCLK	[11] Carbamidomethyl
KIF5C_MOUSE	SLEPCDNTPIIDNITPVVDGISAEK	[5] Carbamidomethyl
LDHB_MOUSE	VIGSGCNLDSAR	[6] Carbamidomethyl
LETM1_MOUSE	GEEITKEEIDILSDACSK	[16] Carbamidomethyl
LGI1_MOUSE	DFDCIITEFAK	[4] Carbamidomethyl
LIS1_MOUSE	LLASCSADMTIK	[5] Carbamidomethyl
LIS1_MOUSE	MVRPNQDGTLIASCSNDQTVR	[14] Carbamidomethyl
LRRC7_MOUSE	SMCAPLPVAAQSTTLPSLSGR	[3] Carbamidomethyl
MIA40_MOUSE	GSDCIDQFR	[4] Carbamidomethyl
MPP2_MOUSE	DLELTPTSGTLCGSLSGK	[12] Carbamidomethyl
MPP2_MOUSE	RDLELTPTSGTLCGSLSGKK	[13] Carbamidomethyl
MPP2_MOUSE	VCVLDVNPQAVK	[2] Carbamidomethyl
MPP6_MOUSE	DWDNSGPFCGTISNK	[9] Carbamidomethyl
MPP6_MOUSE	TCILDVNPQALK	[2] Carbamidomethyl
NDUS1_MOUSE	DCFIVYQGHHGDVGAPMADVILPGAAYTEK	[2] Carbamidomethyl
NDUS1_MOUSE	DLLNKVDSDNLCTEEIFPTEGAGTDLR	[12] Carbamidomethyl
NDUS1_MOUSE	MCLVEIEK	[2] Carbamidomethyl
NDUS1_MOUSE	MLFLLGADGGCITR	[11] Carbamidomethyl
NDUS1_MOUSE	VDSDNLCTEEIFPTEGAGTDLR	[7] Carbamidomethyl

NFASC_MOUSE	DNILIECEAK	[7] Carbamidomethyl
NFASC_MOUSE	DQGSYTCMASTELDQDLAK	[7] Carbamidomethyl
NFASC_MOUSE	ITNVSEEDSGEYFCLASNK	[14] Carbamidomethyl
NFASC_MOUSE	LDCPFFGSPIPTLR	[3] Carbamidomethyl
NFASC_MOUSE	SGGRPEEYEGEYQCFAR	[14] Carbamidomethyl
NFASC_MOUSE	TRLDCPFFGSPIPTLR	[5] Carbamidomethyl
NRCAM_MOUSE	AETYEGVYQCTAR	[10] Carbamidomethyl
NRCAM_MOUSE	DSTGTYTCVAR	[8] Carbamidomethyl
NRCAM_MOUSE	TLQITHVSEADSGNYQCIAK	[17] Carbamidomethyl
NSF_MOUSE	CPTDELSLSNCAVVNEK	[1] Carbamidomethyl |[11] Carbamidomethyl
NSF_MOUSE	GILLYGPPGCGK	[10] Carbamidomethyl
NSF_MOUSE	SQLSCVVVDDIER	[5] Carbamidomethyl
NSF_MOUSE	THPSVVPGCIAFSLPQR	[9] Carbamidomethyl
NSF_MOUSE	VFPPEIVEQMGCK	[12] Carbamidomethyl
NTRI_MOUSE	EQSGEYECSASNDVAAPVVR	[8] Carbamidomethyl
NTRI_MOUSE	GTLQCEASAVPSAEFQWFK	[5] Carbamidomethyl
ODO1_MOUSE	AEQFYCGDTEGK	[6] Carbamidomethyl
ODO1_MOUSE	DVVVDLVCYR	[8] Carbamidomethyl
ODO1_MOUSE	ELEQIFCQFDSKLEAADEGSGDMK	[7] Carbamidomethyl
ODO1_MOUSE	FGLEGCEVLIPALK	[6] Carbamidomethyl
ODO1_MOUSE	ICEEAFTR	[2] Carbamidomethyl
ODO1_MOUSE	RFGLEGCEVLIPALK	[7] Carbamidomethyl
ODO1_MOUSE	SMTCPSTGLEEDVLFHIGK	[4] Carbamidomethyl
ODO1_MOUSE	SMTCPSTGLEEDVLFHIGK	[2] Oxidation (M)|[4] Carbamidomethyl
ODO1_MOUSE	VVNAPIFHVNSDDPEAVMYVCK	[21] Carbamidomethyl
ODO1_MOUSE	YPNAELAWCQEEHK	[9] Carbamidomethyl
ODPX_MOUSE	DVSAPPPVSKPPAPTQPSPQPQIPCPAR	[25] Carbamidomethyl
ODPX_MOUSE	STVPHAYATADCDLGAVLK	[12] Carbamidomethyl

OPA1_MOUSE	EGCTVSPETISLNVK	[3] Carbamidomethyl
PCCA_MOUSE	MADEAVCVGPAPTSK	[7] Carbamidomethyl
PDE2A_MOUSE	ATDQVVALACAFNK	[10] Carbamidomethyl
PDE2A_MOUSE	LVCEDPPHELPQEGK	[3] Carbamidomethyl
PI4KA_MOUSE	ICWQAAIFK	[2] Carbamidomethyl
PI4KA_MOUSE	QNTTLGATQLTERPACVK	[16] Carbamidomethyl
PLCB1_MOUSE	EVIEAIAECAFK	[9] Carbamidomethyl
PLCB1_MOUSE	LTDVAEECQNNQLK	[8] Carbamidomethyl
PLCB1_MOUSE	RVETALEACSLPSSR	[9] Carbamidomethyl
PLCB1_MOUSE	VVLPSLACLR	[8] Carbamidomethyl
PLPR4_MOUSE	NAEGSTVTCTGSIR	[9] Carbamidomethyl
PP2BA_MOUSE	FKEPPAYGPMCDILWSDPLEDFGNEK	[11] Carbamidomethyl
PRDX6_MOUSE	DFTPVCTTELGR	[6] Carbamidomethyl
PTPRD_MOUSE	TATMLCAASGNPDPEITWFK	[6] Carbamidomethyl
PTPRD_MOUSE	TPVDQTGVSGGVASFICQATGDPRPK	[17] Carbamidomethyl
PTPRD_MOUSE	VCLQPIR	[2] Carbamidomethyl
PTPRD_MOUSE	YECVATNSAGTR	[3] Carbamidomethyl
PTPRS_MOUSE	TATMLCAASGNPDPEITWFK	[6] Carbamidomethyl
PTPRS_MOUSE	VCLQPIR	[2] Carbamidomethyl
PYGB_MOUSE	TCAYTNHTVLPEALER	[2] Carbamidomethyl
PYGB_MOUSE	TCFETFPDK	[2] Carbamidomethyl
RAP2B_MOUSE	ALAEEWSCPFMETSAK	[8] Carbamidomethyl
RASH_MOUSE	LNPPDESGPGCMSCK	[11] Carbamidomethyl |[14] Carbamidomethyl
RHOG_MOUSE	YLECSALQQDGVK	[4] Carbamidomethyl
S39AC_MOUSE	YFGTSSSQCMETK	[9] Carbamidomethyl
SAHH2_MOUSE	LCVPAMNVNDSVTK	[2] Carbamidomethyl
SC6A1_MOUSE	QCDNPWNTDR	[2] Carbamidomethyl
SDHB_MOUSE	CHTIMNCTQTCPK	[1] Carbamidomethyl |[7] Carbamidomethyl |[11] Carbamidomethyl

SEPT8_MOUSE	ELEEETNAFNCR	[11] Carbamidomethyl
SEPT8_MOUSE	QYPWGVVQVENENHCDFVK	[15] Carbamidomethyl
SEPT8_MOUSE	RRELEEETNAFNCR	[13] Carbamidomethyl
SEPT8_MOUSE	STLMNTLFNTTFETEEASHHEECVR	[23] Carbamidomethyl
SRBS2_MOUSE	DLMNSEVICSVK	[9] Carbamidomethyl
SSDH_MOUSE	EVGEVLCTDPLVSK	[7] Carbamidomethyl
SSDH_MOUSE	LGTVADCGVPEAR	[7] Carbamidomethyl
STXB1_MOUSE	AAHVFFTDSCPDALFNELVK	[10] Carbamidomethyl
STXB1_MOUSE	LAEQIATLCATLK	[9] Carbamidomethyl
STXB1_MOUSE	YSTHLHLAEDCMK	[11] Carbamidomethyl
SYIM_MOUSE	VHFVPGWDCHGLPIETK	[9] Carbamidomethyl
SYNJ1_MOUSE	TSPCQSPTVPEYSAPSLPIRPSR	[4] Carbamidomethyl
SYNPR_MOUSE	EVLLLMSACK	[9] Carbamidomethyl
SYNPR_MOUSE	LQQVTFEVPTCEGK	[11] Carbamidomethyl
SYT2_MOUSE	LGDICTSLR	[5] Carbamidomethyl
SYT2_MOUSE	LTVCILEAK	[4] Carbamidomethyl
TERA_MOUSE	AIANECQANFISIK	[6] Carbamidomethyl
TERA_MOUSE	LGDVISIQPCPDVK	[10] Carbamidomethyl
TERA_MOUSE	QAAPCVLFFDELDSIAK	[5] Carbamidomethyl
THY1_MOUSE	VTSLTACLVNQNLR	[7] Carbamidomethyl
UBA1_MOUSE	DNPGVVTCLDEAR	[8] Carbamidomethyl
VA0D1_MOUSE	NIVWIAECIAQR	[8] Carbamidomethyl
VDAC2_MOUSE	SCSGVEFSTSGSSNTDTGK	[2] Carbamidomethyl
VDAC2_MOUSE	WCEYGLTFTEK	[2] Carbamidomethyl
VGLU2_MOUSE	ILQGLVEGVTYPACHGIWSK	[14] Carbamidomethyl
