# Supplementary material for: Identification of substrates of palmitoyl protein thioesterase 1 highlights roles of depalmitoylation in disulfide bond formation and synaptic function
Source: PLoS Biol. 2022 Mar 31;20(3):e3001590. doi: 10.1371/journal.pbio.3001590 (PMC9004782; doi:10.1371/journal.pbio.3001590)
Supplement: S4 Table — Proteins identified in the secondary validation screen that were not identified as putative substrates in the primary screen. PPT1, palmitoyl protein thioesterase 1. (PDF) [file pbio.3001590.s005.pdf]

S4 Table. Residual PPT1 substrates. Proteins identified in the secondary validation screen that were not identified as putative substrates in the primary screen.

Uniprot ID	Gene Name
1433E_MOUSE	*Ywhae*
1433F_MOUSE	*Ywhah*
1433G_MOUSE	*Ywhag*
1433S_MOUSE	*Sfn*
1433T_MOUSE	*Ywhaq*
1433Z_MOUSE	*Ywhaz*
2A5E_MOUSE	*Ppp2r5e*
2AAA_MOUSE	*Ppp2r1a*
2AAB_MOUSE	*Ppp2r1b*
2ABA_MOUSE	*Ppp2r2a*
2ABB_MOUSE	*Ppp2r2b*
2ABD_MOUSE	*Ppp2r2d*
2ABG_MOUSE	*Ppp2r2c*
3HIDH_MOUSE	*Hibadh*
4F2_MOUSE	*Slc3a2*
A2MG_MOUSE	*A2m*
A4_MOUSE	*App*
AAK1_MOUSE	*Aak1*
AATC_MOUSE	*Got1*
AATM_MOUSE	*Got2*
ACAD9_MOUSE	*Acad9*
ACADL_MOUSE	*Acadl*
ACADM_MOUSE	*Acadm*
ACADV_MOUSE	*Acadvl*
ACLY_MOUSE	*Acly*
ACO10_MOUSE	*Acot10*
ACO13_MOUSE	*Acot13*
ACOT9_MOUSE	*Acot9*
ACPM_MOUSE	*Ndufab1*
ACSL6_MOUSE	*Acsl6*
ACTA_MOUSE	*Acta2*
ACTB_MOUSE	*Actb*
ACTC_MOUSE	*Actc1*
ACTN2_MOUSE	*Actn2*
ACTN4_MOUSE	*Actn4*
ACYP1_MOUSE	*Acyp1*

ADA22_MOUSE	*Adam22*
ADA23_MOUSE	*Adam23*
ADDA_MOUSE	*Add1*
ADDB_MOUSE	*Add2*
ADT1_MOUSE	*Slc25a4*
ADT2_MOUSE	*Slc25a5*
AFG2H_MOUSE	*Spata5*
AFG32_MOUSE	*Afg3l2*
AGFG1_MOUSE	*Agfg1*
AGK_MOUSE	*Agk*
AHSA1_MOUSE	*Ahsa1*
AINX_MOUSE	*Ina*
AK1A1_MOUSE	*Akr1a1*
AKAP5_MOUSE	*Akap5*
AL1B1_MOUSE	*Aldh1b1*
AL7A1_MOUSE	*Aldh7a1*
ALBU_MOUSE	*Alb*
ALDH2_MOUSE	*Aldh2*
ALDOA_MOUSE	*Aldoa*
ALDOC_MOUSE	*Aldoc*
AMPH_MOUSE	*Amph*
AMPL_MOUSE	*Lap3*
AMRP_MOUSE	*Lrpap1*
ANK2_MOUSE	*Ank2*
ANS1B_MOUSE	*Anks1b*
ANXA5_MOUSE	*Anxa5*
ANXA6_MOUSE	*Anxa6*
ANXA7_MOUSE	*Anxa7*
AOFB_MOUSE	*Maob*
AP180_MOUSE	*Snap91*
AP1B1_MOUSE	*Ap1b1*
AP2A2_MOUSE	*Ap2a2*
AP2S1_MOUSE	*Ap2s1*
AP3B2_MOUSE	*Ap3b2*
AP3D1_MOUSE	*Ap3d1*
AP3M2_MOUSE	*Ap3m2*
APOE_MOUSE	*Apoe*
ARC1A_MOUSE	*Arpc1a*
ARF1_MOUSE	*Arf1*

ARF2_MOUSE	*Arf2*
ARF5_MOUSE	*Arf5*
ARFG1_MOUSE	*Arfgap1*
ARM10_MOUSE	*Armc10*
ARMC1_MOUSE	*Armc1*
ARP2_MOUSE	*Actr2*
ARP3_MOUSE	*Actr3*
ARP3B_MOUSE	*Actr3b*
ARP5L_MOUSE	*Arpc5l*
ARPC2_MOUSE	*Arpc2*
ARPC3_MOUSE	*Arpc3*
ARPC4_MOUSE	*Arpc4*
ARPC5_MOUSE	*Arpc5*
ARSB_MOUSE	*Arsb*
ASGL1_MOUSE	*Asrgl1*
ASNA_MOUSE	*Asna1*
ASSY_MOUSE	*Ass1*
AT2A1_MOUSE	*Atp2a1*
AT2A2_MOUSE	*Atp2a2*
AT2B1_MOUSE	*Atp2b1*
AT2B2_MOUSE	*Atp2b2*
AT5F1_MOUSE	*Atp5pb*
AT8A1_MOUSE	*Atp8a1*
ATIF1_MOUSE	*ATP5IF1*
ATLA1_MOUSE	*Atl1*
ATP5H_MOUSE	*Atp5pd*
ATP5L_MOUSE	*Atp5mg*
ATPA_MOUSE	*Atp5f1a*
ATPB_MOUSE	*Atp5f1b*
ATPD_MOUSE	*Atp5f1d*
ATPK_MOUSE	*Atp5mf*
ATPMD_MOUSE	*Atp5md*
AUHM_MOUSE	*Auh*
AUXI_MOUSE	*Dnajc6*
B2L13_MOUSE	*Bcl2l13*
BACH_MOUSE	*Acot7*
BASP1_MOUSE	*Basp1*
BCAS1_MOUSE	*Bcas1*
BCS1_MOUSE	*Bcs1l*

BDH_MOUSE	*Bdh1*
BIN1_MOUSE	*Bin1*
BIN2_MOUSE	*Bin2*
BIP_MOUSE	*Hspa5*
BORG4_MOUSE	*Cdc42ep4*
BPHL_MOUSE	*Bphl*
BSN_MOUSE	*Bsn*
C1QBP_MOUSE	*C1qbp*
C1TM_MOUSE	*Mthfd1l*
C2C2L_MOUSE	*C2cd2l*
CAD13_MOUSE	*Cdh13*
CADH2_MOUSE	*Cdh2*
CADM1_MOUSE	*Cadm1*
CADM3_MOUSE	*Cadm3*
CADM4_MOUSE	*Cadm4*
CAH2_MOUSE	*Ca2*
CALB1_MOUSE	*Calb1*
CALB2_MOUSE	*Calb2*
CALL3_MOUSE	*Calml3*
CALM1_MOUSE	*Calm1*
CALR_MOUSE	*Calr*
CALU_MOUSE	*Calu*
CALX_MOUSE	*Canx*
CAMKV_MOUSE	*Camkv*
CANB1_MOUSE	*Ppp3r1*
CAND1_MOUSE	*Cand1*
CAP1_MOUSE	*Cap1*
CAP2_MOUSE	*Cap2*
CAPZB_MOUSE	*Capzb*
CATB_MOUSE	*Ctsb*
CAZA1_MOUSE	*Capza1*
CAZA2_MOUSE	*Capza2*
CBR1_MOUSE	*Cbr1*
CC136_MOUSE	*Ccdc136*
CC50A_MOUSE	*Tmem30a*
CC50B_MOUSE	*Tmem30b*
CD166_MOUSE	*Alcam*
CDC37_MOUSE	*Cdc37*
CDC42_MOUSE	*Cdc42*

CEND_MOUSE	*Cend1*
CH10_MOUSE	*Hspe1*
CH60_MOUSE	*Hspd1*
CHP1_MOUSE	*Chp1*
CLAP2_MOUSE	*Clasp2*
CLCA_MOUSE	*Clta*
CLCB_MOUSE	*Cltb*
CLD11_MOUSE	*Cldn11*
CLIC4_MOUSE	*Clic4*
CMC1_MOUSE	*Slc25a12*
CMTD1_MOUSE	*Comtd1*
CN37_MOUSE	*Cnp*
CNRP1_MOUSE	*Cnrip1*
CNTN1_MOUSE	*Cntn1*
CNTN2_MOUSE	*Cntn2*
CNTP2_MOUSE	*Cntnap2*
CO1A1_MOUSE	*Col1a1*
CO3_MOUSE	*C3*
CO4B_MOUSE	*C4b*
COF1_MOUSE	*Cfl1*
COQ9_MOUSE	*Coq9*
COR1A_MOUSE	*Coro1a*
COR1B_MOUSE	*Coro1b*
COR1C_MOUSE	*Coro1c*
COX5A_MOUSE	*Cox5a*
COX5B_MOUSE	*Cox5b*
CPLX1_MOUSE	*Cplx1*
CPLX2_MOUSE	*Cplx2*
CPLX3_MOUSE	*Cplx3*
CPNE6_MOUSE	*Cpne6*
CRK_MOUSE	*Crk*
CRKL_MOUSE	*Crkl*
CRYAB_MOUSE	*Cryab*
CRYM_MOUSE	*Crym*
CSK21_MOUSE	*Csnk2a1*
CSK2B_MOUSE	*Csnk2b*
CSKI1_MOUSE	*Caskin1*
CSN1_MOUSE	*Gps1*
CSN2_MOUSE	*Cops2*

CSN4_MOUSE	*Cops4*
CSPG5_MOUSE	*Cspg5*
CSRP1_MOUSE	*Csrp1*
CTBP1_MOUSE	*Ctbp1*
CTNB1_MOUSE	*Ctnnb1*
CTND2_MOUSE	*Ctnnd2*
CTTB2_MOUSE	*Cttnbp2*
CX6B1_MOUSE	*Cox6b1*
CY1_MOUSE	*Cyc1*
CYC_MOUSE	*Cycs*
CYC2_MOUSE	*Cyct*
CYFP2_MOUSE	*Cyfip2*
DBNL_MOUSE	*Dbnl*
DC1I1_MOUSE	*Dync1i1*
DC1I2_MOUSE	*Dync1i2*
DC1L1_MOUSE	*Dync1li1*
DCTN2_MOUSE	*Dctn2*
DDAH1_MOUSE	*Ddah1*
DECR_MOUSE	*Decr1*
DEMA_MOUSE	*Dmtn*
DESM_MOUSE	*Des*
DHE3_MOUSE	*Glud1*
DHPR_MOUSE	*Qdpr*
DIRA2_MOUSE	*Diras2*
DLDH_MOUSE	*Dld*
DLG1_MOUSE	*Dlg1*
DLG2_MOUSE	*Dlg2*
DLG3_MOUSE	*Dlg3*
DLG4_MOUSE	*Dlg4*
DLGP4_MOUSE	*Dlgap4*
DMXL1_MOUSE	*Dmxl1*
DNM1L_MOUSE	*Dnm1l*
DOC2B_MOUSE	*Doc2b*
DOPD_MOUSE	*Ddt*
DPP10_MOUSE	*Dpp10*
DPYL1_MOUSE	*Crmp1*
DPYL2_MOUSE	*Dpysl2*
DPYL3_MOUSE	*Dpysl3*
DPYL4_MOUSE	*Dpysl4*

DPYL5_MOUSE	*Dpysl5*
DREB_MOUSE	*Dbn1*
DUS3_MOUSE	*Dusp3*
DYHC1_MOUSE	*Dync1h1*
DYN3_MOUSE	*Dnm3*
E41L1_MOUSE	*Epb41l1*
E41L2_MOUSE	*Epb41l2*
E41L3_MOUSE	*Epb41l3*
EAA1_MOUSE	*Slc1a3*
EAA2_MOUSE	*Slc1a2*
ECHA_MOUSE	*Hadha*
ECHB_MOUSE	*Hadhb*
ECHM_MOUSE	*Echs1*
EF1A1_MOUSE	*Eef1a1*
EF1A2_MOUSE	*Eef1a2*
EF1G_MOUSE	*Eef1g*
EF2_MOUSE	*Eef2*
EFHD1_MOUSE	*Efhd1*
EFHD2_MOUSE	*Efhd2*
EHD1_MOUSE	*Ehd1*
EHD3_MOUSE	*Ehd3*
ELMO2_MOUSE	*Elmo2*
ENOA_MOUSE	*Eno1*
ENOG_MOUSE	*Eno2*
ENPL_MOUSE	*Hsp90b1*
EPN1_MOUSE	*Epn1*
ERC2_MOUSE	*Erc2*
ERMIN_MOUSE	*Ermn*
ERP44_MOUSE	*Erp44*
ETFA_MOUSE	*Etfa*
ETFB_MOUSE	*Etfb*
ETFD_MOUSE	*Etfdh*
EXOG_MOUSE	*Exog*
EZRI_MOUSE	*Ezr*
FABP5_MOUSE	*Fabp5*
FABPH_MOUSE	*Fabp3*
FAHD2_MOUSE	*Fahd2*
FKB1A_MOUSE	*Fkbp1a*
FKBP2_MOUSE	*Fkbp2*

FLOT1_MOUSE	*Flot1*
FLOT2_MOUSE	*Flot2*
FRIH_MOUSE	*Fth1*
FSCN1_MOUSE	*Fscn1*
G3P_MOUSE	*Gapdh*
G3PT_MOUSE	*Gapdhs*
G6PI_MOUSE	*Gpi*
GABT_MOUSE	*Abat*
GAK_MOUSE	*Gak*
GAL3A_MOUSE	*Gatd3a*
GANAB_MOUSE	*Ganab*
GARS_MOUSE	*Gars1*
GBB1_MOUSE	*Gnb1*
GBB3_MOUSE	*Gnb3*
GBRA1_MOUSE	*Gabra1*
GD1L1_MOUSE	*Gdap1l1*
GDAP1_MOUSE	*Gdap1*
GDIA_MOUSE	*Gdi1*
GDIB_MOUSE	*Gdi2*
GDIR1_MOUSE	*Arhgdia*
GELS_MOUSE	*Gsn*
GEPH_MOUSE	*Gphn*
GFAP_MOUSE	*Gfap*
GGT7_MOUSE	*Ggt7*
GHC1_MOUSE	*Slc25a22*
GHC2_MOUSE	*Slc25a18*
GIT1_MOUSE	*Git1*
GLNA_MOUSE	*Glul*
GLO2_MOUSE	*Hagh*
GLOD4_MOUSE	*Glod4*
GLPK_MOUSE	*Gk*
GLPK2_MOUSE	*Gk2*
GLRX5_MOUSE	*Glrx5*
GLSK_MOUSE	*Gls*
GLU2B_MOUSE	*Prkcsh*
GMFB_MOUSE	*Gmfb*
GMFG_MOUSE	*Gmfg*
GNA11_MOUSE	*Gna11*
GNA12_MOUSE	*Gna12*

GNAS1_MOUSE	*Gnas*
GNB5_MOUSE	*Gnb5*
GP158_MOUSE	*Gpr158*
GPC1_MOUSE	*Gpc1*
GPD1L_MOUSE	*Gpd1l*
GPDM_MOUSE	*Gpd2*
GPM6B_MOUSE	*Gpm6b*
GRB2_MOUSE	*Grb2*
GRIN1_MOUSE	*Gprin1*
GRM3_MOUSE	*Grm3*
GRP75_MOUSE	*Hspa9*
GRPE1_MOUSE	*Grpel1*
GSTM1_MOUSE	*Gstm1*
GSTM3_MOUSE	*Gstm3*
GSTM5_MOUSE	*Gstm5*
GSTM6_MOUSE	*Gstm6*
GSTP1_MOUSE	*Gstp1*
GSTP2_MOUSE	*Gstp2*
GTR3_MOUSE	*Slc2a3*
GUAD_MOUSE	*Gda*
H4_MOUSE	*H4c1*
HBA_MOUSE	*Hba*
HBAZ_MOUSE	*Hbz*
HBB1_MOUSE	*Hbb-b1*
HCD2_MOUSE	*Hsd17b10*
HCDH_MOUSE	*Hadh*
HDHD2_MOUSE	*Hdhd2*
HECAM_MOUSE	*Hepacam*
HEMH_MOUSE	*Fech*
HGS_MOUSE	*Hgs*
HIBCH_MOUSE	*Hibch*
HINT2_MOUSE	*Hint2*
HMGCL_MOUSE	*Hmgcl*
HMOX2_MOUSE	*Hmox2*
HNRPK_MOUSE	*Hnrnpk*
HOME1_MOUSE	*Homer1*
HOME3_MOUSE	*Homer3*
HPCA_MOUSE	*Hpca*
HPCL4_MOUSE	*Hpcal4*

HPRT_MOUSE	*Hprt1*
HS105_MOUSE	*Hsph1*
HS12B_MOUSE	*Hspa12b*
HS71A_MOUSE	*Hspa1a*
HS74L_MOUSE	*Hspa4l*
HS90A_MOUSE	*Hsp90aa1*
HS90B_MOUSE	*Hsp90ab1*
HSP72_MOUSE	*Hspa2*
HSP7C_MOUSE	*Hspa8*
ICAM5_MOUSE	*Icam5*
IDH3A_MOUSE	*Idh3a*
IDHC_MOUSE	*Idh1*
IDHG1_MOUSE	*Idh3g*
IDHP_MOUSE	*Idh2*
IF4B_MOUSE	*Eif4b*
IF4H_MOUSE	*Eif4h*
IF5A1_MOUSE	*Eif5a*
IF5A2_MOUSE	*Eif5a2*
IGSF8_MOUSE	*Igsf8*
IMB1_MOUSE	*Kpnb1*
IPO5_MOUSE	*Ipo5*
IPYR_MOUSE	*Ppa1*
IPYR2_MOUSE	*Ppa2*
IQEC1_MOUSE	*Iqsec1*
IQEC2_MOUSE	*Iqsec2*
ITIH2_MOUSE	*Itih2*
K0513_MOUSE	*Kiaa0513*
KAD1_MOUSE	*Ak1*
KAD2_MOUSE	*Ak2*
KAD3_MOUSE	*Ak3*
KAD4_MOUSE	*Ak4*
KAP2_MOUSE	*Prkar2a*
KAP3_MOUSE	*Prkar2b*
KCAB2_MOUSE	*Kcnab2*
KCC2A_MOUSE	*Camk2a*
KCC2B_MOUSE	*Camk2b*
KCC2G_MOUSE	*Camk2g*
KCRS_MOUSE	*Ckmt2*
KCRU_MOUSE	*Ckmt1*

KCY_MOUSE	*Cmpk1*
KIF2A_MOUSE	*Kif2a*
KLC1_MOUSE	*Klc1*
KLC2_MOUSE	*Klc2*
KPCB_MOUSE	*Prkcb*
KPCG_MOUSE	*Prkcg*
KPYM_MOUSE	*Pkm*
KPYR_MOUSE	*Pklr*
L1CAM_MOUSE	*L1cam*
LACTB_MOUSE	*Lactb*
LANC2_MOUSE	*Lancl2*
LASP1_MOUSE	*Lasp1*
LDHA_MOUSE	*Ldha*
LDHC_MOUSE	*Ldhc*
LGUL_MOUSE	*Glo1*
LIGO1_MOUSE	*Lingo1*
LIN7A_MOUSE	*Lin7a*
LIPA2_MOUSE	*Ppfia2*
LNEBL_MOUSE	*Nebl*
LONM_MOUSE	*Lonp1*
LPPRC_MOUSE	*Lrpprc*
LRC59_MOUSE	*Lrrc59*
LSAMP_MOUSE	*Lsamp*
LXN_MOUSE	*Lxn*
LY6H_MOUSE	*Ly6h*
LYAG_MOUSE	*Gaa*
LYRIC_MOUSE	*Mtdh*
MAG_MOUSE	*Mag*
MAOM_MOUSE	*Me2*
MAON_MOUSE	*Me3*
MAP1A_MOUSE	*Map1a*
MAP1B_MOUSE	*Map1b*
MAP4_MOUSE	*Map4*
MAP6_MOUSE	*Map6*
MARC2_MOUSE	*Marc2*
MARCS_MOUSE	*Marcks*
MARE1_MOUSE	*Mapre1*
MARE2_MOUSE	*Mapre2*
MARE3_MOUSE	*Mapre3*

MBP_MOUSE	*Mbp*
MCU_MOUSE	*Mcu*
MDHC_MOUSE	*Mdh1*
MDHM_MOUSE	*Mdh2*
MFF_MOUSE	*Mff*
MFN2_MOUSE	*Mfn2*
MFR1L_MOUSE	*Mtfr1l*
MGLL_MOUSE	*Mgll*
MIC13_MOUSE	*Micos13*
MIC19_MOUSE	*Chchd3*
MIC25_MOUSE	*Chchd6*
MIC26_MOUSE	*Apoo*
MIC60_MOUSE	*Immt*
MIF_MOUSE	*Mif*
MK01_MOUSE	*Mapk1*
MK03_MOUSE	*Mapk3*
MK04_MOUSE	*Mapk4*
ML12B_MOUSE	*Myl12b*
MMSA_MOUSE	*Aldh6a1*
MOG_MOUSE	*Mog*
MP2K1_MOUSE	*Map2k1*
MPCP_MOUSE	*Slc25a3*
MPPA_MOUSE	*Pmpca*
MTAP2_MOUSE	*Map2*
MTCH1_MOUSE	*Mtch1*
MTCH2_MOUSE	*Mtch2*
MTCL1_MOUSE	*Mtcl1*
MTX1_MOUSE	*Mtx1*
MTX2_MOUSE	*Mtx2*
MUTA_MOUSE	*Mmut*
MY18A_MOUSE	*Myo18a*
MYH10_MOUSE	*Myh10*
MYL6_MOUSE	*Myl6*
MYL6B_MOUSE	*Myl6b*
MYL9_MOUSE	*Myl9*
MYO5A_MOUSE	*Myo5a*
MYO5B_MOUSE	*Myo5b*
MYPR_MOUSE	*Plp1*
NAC2_MOUSE	*Slc8a2*

NB5R1_MOUSE	*Cyb5r1*
NB5R3_MOUSE	*Cyb5r3*
NCAM1_MOUSE	*Ncam1*
NCAM2_MOUSE	*Ncam2*
NCAN_MOUSE	*Ncan*
NCDN_MOUSE	*Ncdn*
NCEH1_MOUSE	*Nceh1*
NCKPL_MOUSE	*Nckap1l*
NCS1_MOUSE	*Ncs1*
NDKB_MOUSE	*Nme2*
NDRG1_MOUSE	*Ndrg1*
NDRG2_MOUSE	*Ndrg2*
NDRG3_MOUSE	*Ndrg3*
NDUA2_MOUSE	*Ndufa2*
NDUA4_MOUSE	*Ndufa4*
NDUA5_MOUSE	*Ndufa5*
NDUA7_MOUSE	*Ndufa7*
NDUA8_MOUSE	*Ndufa8*
NDUA9_MOUSE	*Ndufa9*
NDUAA_MOUSE	*Ndufa10*
NDUAC_MOUSE	*Ndufa12*
NDUAD_MOUSE	*Ndufa13*
NDUB3_MOUSE	*Ndufb3*
NDUB4_MOUSE	*Ndufb4*
NDUB5_MOUSE	*Ndufb5*
NDUB6_MOUSE	*Ndufb6*
NDUB7_MOUSE	*Ndufb7*
NDUB8_MOUSE	*Ndufb8*
NDUB9_MOUSE	*Ndufb9*
NDUBA_MOUSE	*Ndufb10*
NDUBB_MOUSE	*Ndufb11*
NDUC2_MOUSE	*Ndufc2*
NDUF2_MOUSE	*Ndufaf2*
NDUS2_MOUSE	*Ndufs2*
NDUS3_MOUSE	*Ndufs3*
NDUS4_MOUSE	*Ndufs4*
NDUS5_MOUSE	*Ndufs5*
NDUS6_MOUSE	*Ndufs6*
NDUS7_MOUSE	*Ndufs7*

NDUS8_MOUSE	*Ndufs8*
NDUV1_MOUSE	*Ndufv1*
NDUV2_MOUSE	*Ndufv2*
NEB2_MOUSE	*Ppp1r9b*
NECP1_MOUSE	*Necap1*
NECT1_MOUSE	*Nectin1*
NEGR1_MOUSE	*Negr1*
NEUM_MOUSE	*Gap43*
NFH_MOUSE	*Nefh*
NFL_MOUSE	*Nefl*
NFM_MOUSE	*Nefm*
NFS1_MOUSE	*Nfs1*
NFU1_MOUSE	*Nfu1*
NHRF1_MOUSE	*Slc9a3r1*
NIPS1_MOUSE	*Nipsnap1*
NIPS2_MOUSE	*Nipsnap2*
NLGN2_MOUSE	*Nlgn2*
NLGN3_MOUSE	*Nlgn3*
NMDE2_MOUSE	*Grin2b*
NMDZ1_MOUSE	*Grin1*
NNRD_MOUSE	*Naxd*
NNRE_MOUSE	*Naxe*
NOE1_MOUSE	*Olfm1*
NP1L1_MOUSE	*Nap1l1*
NP1L4_MOUSE	*Nap1l4*
NPTN_MOUSE	*Nptn*
NPTX1_MOUSE	*Nptx1*
NPTXR_MOUSE	*Nptxr*
NRX1A_MOUSE	*Nrxn1*
NRX1B_MOUSE	*Nrxn1*
NRX3B_MOUSE	*Nrxn3*
NSF1C_MOUSE	*Nsfl1c*
NT5C_MOUSE	*Nt5c*
NT5D3_MOUSE	*Nt5dc3*
NTRK2_MOUSE	*Ntrk2*
OCAD1_MOUSE	*Ociad1*
OCAD2_MOUSE	*Ociad2*
ODO2_MOUSE	*Dlst*
ODP2_MOUSE	*Dlat*

ODPA_MOUSE	*Pdha1*
ODPB_MOUSE	*Pdhb*
OMGP_MOUSE	*Omg*
OMP_MOUSE	*Omp*
OST48_MOUSE	*Ddost*
OTUB1_MOUSE	*Otub1*
OX2G_MOUSE	*Cd200*
PA1B2_MOUSE	*Pafah1b2*
PACN1_MOUSE	*Pacsin1*
PACS1_MOUSE	*Pacs1*
PAK1_MOUSE	*Pak1*
PALM_MOUSE	*Palm*
PARK7_MOUSE	*Park7*
PCBP1_MOUSE	*Pcbp1*
PCBP2_MOUSE	*Pcbp2*
PCCB_MOUSE	*Pccb*
PCLO_MOUSE	*Pclo*
PDCD6_MOUSE	*Pdcd6*
PDIA1_MOUSE	*P4hb*
PDIA3_MOUSE	*Pdia3*
PDIA6_MOUSE	*Pdia6*
PDXK_MOUSE	*Pdxk*
PEA15_MOUSE	*Pea15*
PEBP1_MOUSE	*Pebp1*
PFKAL_MOUSE	*Pfkl*
PFKAM_MOUSE	*Pfkm*
PFKAP_MOUSE	*Pfkp*
PGAM1_MOUSE	*Pgam1*
PGAM2_MOUSE	*Pgam2*
PGCB_MOUSE	*Bcan*
PGES2_MOUSE	*Ptges2*
PGK1_MOUSE	*Pgk1*
PGM2L_MOUSE	*Pgm2l1*
PGRC1_MOUSE	*Pgrmc1*
PHB_MOUSE	*Phb*
PHB2_MOUSE	*Phb2*
PHF24_MOUSE	*Phf24*
PHIPL_MOUSE	*Phyhipl*
PI51C_MOUSE	*Pip5k1c*

PIMT_MOUSE	*Pcmt1*
PIPNA_MOUSE	*Pitpna*
PKP4_MOUSE	*Pkp4*
PLEC_MOUSE	*Plec*
PP1A_MOUSE	*Ppp1ca*
PP1R7_MOUSE	*Ppp1r7*
PP2AB_MOUSE	*Ppp2cb*
PP2BB_MOUSE	*Ppp3cb*
PP2BC_MOUSE	*Ppp3cc*
PPIA_MOUSE	*Ppia*
PPIB_MOUSE	*Ppib*
PRDX1_MOUSE	*Prdx1*
PRDX2_MOUSE	*Prdx2*
PRDX3_MOUSE	*Prdx3*
PRDX5_MOUSE	*Prdx5*
PROF1_MOUSE	*Pfn1*
PROF2_MOUSE	*Pfn2*
PRRT2_MOUSE	*Prrt2*
PSA_MOUSE	*Npepps*
PSA1_MOUSE	*Psma1*
PSA5_MOUSE	*Psma5*
PSA7_MOUSE	*Psma7*
PSD3_MOUSE	*Psd3*
PTN11_MOUSE	*Ptpn11*
PTPR2_MOUSE	*Ptprn2*
PTPRZ_MOUSE	*Ptprz1*
PURA_MOUSE	*Pura*
PURB_MOUSE	*Purb*
PYC_MOUSE	*Pc*
PYGM_MOUSE	*Pygm*
QCR1_MOUSE	*Uqcrc1*
QCR2_MOUSE	*Uqcrc2*
QCR7_MOUSE	*Uqcrb*
QCR8_MOUSE	*Uqcrq*
RAB10_MOUSE	*Rab10*
RAB14_MOUSE	*Rab14*
RAB2A_MOUSE	*Rab2a*
RAB3A_MOUSE	*Rab3a*
RAB3C_MOUSE	*Rab3c*

RAB5B_MOUSE	*Rab5b*
RAB5C_MOUSE	*Rab5c*
RAB6A_MOUSE	*Rab6a*
RAC1_MOUSE	*Rac1*
RAC2_MOUSE	*Rac2*
RACK1_MOUSE	*Rack1*
RALA_MOUSE	*Rala*
RANG_MOUSE	*Ranbp1*
RAP2A_MOUSE	*Rap2a*
RASL1_MOUSE	*Rasal1*
RB11A_MOUSE	*Rab11a*
RB11B_MOUSE	*Rab11b*
RB27B_MOUSE	*Rab27b*
RCN2_MOUSE	*Rcn2*
RD23B_MOUSE	*Rad23b*
RGS6_MOUSE	*Rgs6*
RGS7_MOUSE	*Rgs7*
RHG01_MOUSE	*Arhgap1*
RHOA_MOUSE	*Rhoa*
RHOB_MOUSE	*Rhob*
RIDA_MOUSE	*Rida*
RILP_MOUSE	*Rilp*
RIMS1_MOUSE	*Rims1*
RL13_MOUSE	*Rpl13*
RLA2_MOUSE	*Rplp2*
RP3A_MOUSE	*Rph3a*
RS18_MOUSE	*Rps18*
RS19_MOUSE	*Rps19*
RS28_MOUSE	*Rps28*
RT23_MOUSE	*Mrps23*
RT36_MOUSE	*Mrps36*
RTN1_MOUSE	*Rtn1*
RTN3_MOUSE	*Rtn3*
RTN4_MOUSE	*Rtn4*
RUFY3_MOUSE	*Rufy3*
S12A5_MOUSE	*Slc12a5*
S4A10_MOUSE	*Slc4a10*
S4A4_MOUSE	*Slc4a4*
S4A8_MOUSE	*Slc4a8*

S6A11_MOUSE	*Slc6a11*
S6A17_MOUSE	*Slc6a17*
SAHH3_MOUSE	*Ahcyl2*
SAM50_MOUSE	*Samm50*
SC22B_MOUSE	*Sec22b*
SC6A6_MOUSE	*Slc6a6*
SCMC3_MOUSE	*Slc25a23*
SCN2A_MOUSE	*Scn2a*
SCN2B_MOUSE	*Scn2b*
SCOT1_MOUSE	*Oxct1*
SCPDL_MOUSE	*Sccpdh*
SCRN1_MOUSE	*Scrn1*
SDHA_MOUSE	*Sdha*
SEP11_MOUSE	*Septin11*
SEPT3_MOUSE	*Septin3*
SEPT4_MOUSE	*Septin4*
SEPT5_MOUSE	*Septin5*
SEPT6_MOUSE	*Septin6*
SEPT7_MOUSE	*Septin7*
SEPT9_MOUSE	*Septin9*
SERA_MOUSE	*Phgdh*
SERC_MOUSE	*Psat1*
SFXN5_MOUSE	*Sfxn5*
SGIP1_MOUSE	*Sgip1*
SGTA_MOUSE	*Sgta*
SH3G1_MOUSE	*Sh3gl1*
SH3G2_MOUSE	*Sh3gl2*
SHAN1_MOUSE	*Shank1*
SHAN2_MOUSE	*Shank2*
SHAN3_MOUSE	*Shank3*
SHLB2_MOUSE	*Sh3glb2*
SHPS1_MOUSE	*Sirpa*
SHSA7_MOUSE	*Shisa7*
SIR2_MOUSE	*Sirt2*
SKP1_MOUSE	*Skp1*
SLIRP_MOUSE	*Slirp*
SMCE1_MOUSE	*Smarce1*
SNAA_MOUSE	*Napa*
SNAB_MOUSE	*Napb*

SNAG_MOUSE	*Napg*
SNG1_MOUSE	*Syngr1*
SNG3_MOUSE	*Syngr3*
SNP23_MOUSE	*Snap23*
SNP25_MOUSE	*Snap25*
SNPH_MOUSE	*Snph*
SNX1_MOUSE	*Snx1*
SODC_MOUSE	*Sod1*
SODM_MOUSE	*Sod2*
SOGA3_MOUSE	*Soga3*
SPRE_MOUSE	*Spr*
SPTB1_MOUSE	*Sptb*
SPTB2_MOUSE	*Sptbn1*
SPTN1_MOUSE	*Sptan1*
SRC8_MOUSE	*Cttn*
SRCN1_MOUSE	*Srcin1*
SRR_MOUSE	*Srr*
SSBP_MOUSE	*Ssbp1*
STAM1_MOUSE	*Stam*
STIP1_MOUSE	*Stip1*
STMN1_MOUSE	*Stmn1*
STX12_MOUSE	*Stx12*
STX1A_MOUSE	*Stx1a*
STX1B_MOUSE	*Stx1b*
STX2_MOUSE	*Stx2*
SUCA_MOUSE	*Suclg1*
SUCB1_MOUSE	*Sucla2*
SV2A_MOUSE	*Sv2a*
SV2B_MOUSE	*Sv2b*
SYAC_MOUSE	*Aars*
SYBU_MOUSE	*Sybu*
SYGP1_MOUSE	*Syngap1*
SYN1_MOUSE	*Syn1*
SYN2_MOUSE	*Syn2*
SYN3_MOUSE	*Syn3*
SYNPO_MOUSE	*Synpo*
SYPH_MOUSE	*Syp*
SYSC_MOUSE	*Sars*
SYT1_MOUSE	*Syt1*

SYUB_MOUSE	*Sncb*
TAGL_MOUSE	*Tagln*
TAGL2_MOUSE	*Tagln2*
TAGL3_MOUSE	*Tagln3*
TALDO_MOUSE	*Taldo1*
TAU_MOUSE	*Mapt*
TBA4A_MOUSE	*Tuba4a*
TBB2A_MOUSE	*Tubb2a*
TBB3_MOUSE	*Tubb3*
TBB4A_MOUSE	*Tubb4a*
TBB4B_MOUSE	*Tubb4b*
TBB5_MOUSE	*Tubb5*
TBB6_MOUSE	*Tubb6*
TBC8B_MOUSE	*Tbc1d8b*
TCPA_MOUSE	*Tcp1*
TCPB_MOUSE	*Cct2*
TCPD_MOUSE	*Cct4*
TCPE_MOUSE	*Cct5*
TCPG_MOUSE	*Cct3*
TCPQ_MOUSE	*Cct8*
TCPW_MOUSE	*Cct6b*
TCPZ_MOUSE	*Cct6a*
TCTP_MOUSE	*Tpt1*
TENR_MOUSE	*Tnr*
THEM4_MOUSE	*Them4*
THIL_MOUSE	*Acat1*
THIM_MOUSE	*Acaa2*
THIO_MOUSE	*Txn*
THIOM_MOUSE	*Txn2*
THTM_MOUSE	*Mpst*
THTR_MOUSE	*Tst*
TIM44_MOUSE	*Timm44*
TIM50_MOUSE	*Timm50*
TKT_MOUSE	*Tkt*
TLN2_MOUSE	*Tln2*
TM1L2_MOUSE	*Tom1l2*
TMM65_MOUSE	*Tmem65*
TMOD2_MOUSE	*Tmod2*
TMOD3_MOUSE	*Tmod3*

TNNC2_MOUSE	*Tnnc2*
TOLIP_MOUSE	*Tollip*
TOM1_MOUSE	*Tom1*
TOM22_MOUSE	*Tomm22*
TOM70_MOUSE	*Tomm70*
TPD52_MOUSE	*Tpd52*
TPD54_MOUSE	*Tpd52l2*
TPIS_MOUSE	*Tpi1*
TPM2_MOUSE	*Tpm2*
TPM3_MOUSE	*Tpm3*
TPM4_MOUSE	*Tpm4*
TPPP_MOUSE	*Tppp*
TPRGL_MOUSE	*Tprg1l*
TRAP1_MOUSE	*Trap1*
TRFE_MOUSE	*Tf*
TTHY_MOUSE	*Ttr*
TTYH1_MOUSE	*Ttyh1*
TWF2_MOUSE	*Twf2*
TXTP_MOUSE	*Slc25a1*
UB2V1_MOUSE	*Ube2v1*
UBA1Y_MOUSE	*Uba1y*
UBE2N_MOUSE	*Ube2n*
UBP5_MOUSE	*Usp5*
UCHL1_MOUSE	*Uchl1*
UCHL3_MOUSE	*Uchl3*
UCHL4_MOUSE	*Uchl4*
UGPA_MOUSE	*Ugp2*
VAMP2_MOUSE	*Vamp2*
VAMP3_MOUSE	*Vamp3*
VAPA_MOUSE	*Vapa*
VAPB_MOUSE	*Vapb*
VAT1L_MOUSE	*Vat1l*
VATA_MOUSE	*Atp6v1a*
VATB2_MOUSE	*Atp6v1b2*
VATC1_MOUSE	*Atp6v1c1*
VATD_MOUSE	*Atp6v1d*
VATE2_MOUSE	*Atp6v1e2*
VATF_MOUSE	*Atp6v1f*
VATG2_MOUSE	*Atp6v1g2*

VATH_MOUSE	*Atp6v1h*
VDAC1_MOUSE	*Vdac1*
VDAC3_MOUSE	*Vdac3*
VGLU1_MOUSE	*Slc17a7*
VGLU3_MOUSE	*Slc17a8*
VINC_MOUSE	*Vcl*
VP26B_MOUSE	*Vps26b*
VPP1_MOUSE	*Atp6v0a1*
VPP2_MOUSE	*Atp6v0a2*
WASF1_MOUSE	*Wasf1*
WASF3_MOUSE	*Wasf3*
WBP2_MOUSE	*Wbp2*
WDR1_MOUSE	*Wdr1*
WDR7_MOUSE	*Wdr7*
YKT6_MOUSE	*Ykt6*
