# Supplementary material for: Identification of substrates of palmitoyl protein thioesterase 1 highlights roles of depalmitoylation in disulfide bond formation and synaptic function
Source: PLoS Biol. 2022 Mar 31;20(3):e3001590. doi: 10.1371/journal.pbio.3001590 (PMC9004782; doi:10.1371/journal.pbio.3001590)
Supplement: S5 Table — Protein localization and disulfide bond data were gathered from the UniProt entry for each protein. Carbamidomethyl (carba.) sites that were not validated in the tertiary screen are indicated by an asterisk (*). Disulfide bonds validated by a dNEM moiety in the tertiary screen are indicated (X). GPI, GPi anchor; MM, mitochondrial membrane; SPM, synaptic plasma membrane; PPT1, palmitoyl protein thioesterase 1. (PDF) [file pbio.3001590.s006.pdf]

S5 Table. Carbamidomethyl-modified peptides for high-confidence PPT1 substrates. Protein localization and disulfide bond data were gathered from the UniProt entry for each protein. Carbamidomethyl (carba.) sites that were *not* validated in the tertiary screen are indicated by an asterisk (*). Disulfide bonds validated by a dNEM moiety in the tertiary screen are indicated (X). Mitochondrial membrane (MM), synaptic plasma membrane (SPM), GPi anchor (GPI).

UniProt Accession	Peptide	Carba. site in peptide	Protein localization	Peptide site in protein	Peptide localization	Cysteine site	Disulfide Bond	dNEM
AT1A1_MOUSE	LIIVEG**C**QR	7	SPM	699-707	Cytoplasmic	705		
AT1A2_MOUSE	LIIVEG**C**QR	7	SPM	696-704	Cytoplasmic	702		
AT1A3_MOUSE	LIIVEG**C**QR	7	SPM	689-697	Cytoplasmic	705		
AT1A3_MOUSE	VLGF**C**HYYLPEEQFPK	5	SPM	542-557	Cytoplasmic	546		
AT1A3_MOUSE	YNTD**C**VQGLTHSK	5	SPM	45-56	Cytoplasmic	49		
AT1B1_MOUSE	DSAQKDDMIFED**C**GNVPSEPK	13	SPM	114-134	Extracellular	126	to 149	X
AT1B1_MOUSE	YNPNVLPVQ**C**TGK	10	SPM	205-217	Extracellular	214	to 277	
AT1B2_MOUSE	TQLGD**C**SGIGDPTHYGYSTGQP**C**VFIK	6<br>23	SPM	155-181	Extracellular	160<br>177	to 177<br>to 160	X
AT1B2_MOUSE	FLNVTPNVEVNVE**C**R	14	SPM	248-262	Extracellular	261	to 200	X
ATPO_MOUSE	GEVP**C**TVTTASPLDDAVLSELK	5	MM	137-158		141		
BASI_MOUSE	SGEYS**C**IFLPEPVGR	6	SPM	198-212	Extracellular	203	to 157	
CADM2_MOUSE	IIPSTPFPQEGQALTLT**C**ESK	18	SPM	231-251	Extracellular	248	to 296	
CADM2_MOUSE	AYLTVLGVPEKPQISGFSSPVMEGDLMQLT**C**K	31	SPM	116-147	Extracellular	146	to 203	
CD81_MOUSE	TFHETLN**CC**GSNALTTLTTTILR	8<br>9	SPM	149-171	Extracellular	156<br>157	to 190<br>to 175	
CISD1_MOUSE	KFPF**C**DGAHIK	5	MM	79-89		83		
DYL2_MOUSE	NADMSEDMQQDAVD**C**ATQAMEK	15	Cytosolic	10-31		24		
GBB2_MOUSE	VS**C**LGVTDDGMAVATGSWDSFLK	3	Cytosolic	315-337		317		
GPM6A_MOUSE	KI**C**TASENFLR	3	SPM	190-200	Extracellular	192	to 174	X
GRIA1_MOUSE	RGNAGD**C**LANPAVPWGQGIDIQR	7	SPM	317-339	Extracellular	323	to 75	
KCRB_MOUSE	F**C**TGLTQIETLFK	2	Cytosolic	253-265		254		
LDHB_MOUSE	VIGSG**C**NLDSAR	6	Cytosolic	159-170		164		
LGI1_MOUSE	DFD**C**IITEFAK	4	Secreted	218-228		221		
NDKA_MOUSE	GDF**C**IQVGR	4	Nucleus	106-114		109		
NDUS1_MOUSE	M**C**LVEIEK	2*	MM	77-84		78		

NFASC_MOUSE	SGGRPEEYEGEYQ**C**FAR	14	SPM	105-121	Extracellular	118	to 63	
NRCAM_MOUSE	DSTGTYT**C**VAR	8*	SPM	512-521	Extracellular	519	to 470	
NRCAM_MOUSE	TLQITHVSEADSGNYQ**C**IAK	17	SPM	318-337	Extracellular	334	to 286	
NTRI_MOUSE	GTLQ**C**EASAVPSAEFQWFK	5	SPM-GPI	239-257	Extracellular	243	to 295	
NTRI_MOUSE	EQSGEYE**C**SASNDVAAPVVR	8	SPM-GPI	194-213		201	to 257	
STXB1_MOUSE	AAHVFFTDS**C**PDALFNELVK	10	Cytosolic	101-120		110		
THY1_MOUSE	VTSLTA**C**LVNQNLR	7	SPM-GPI	22-35	Extracellular	28	to 131	X
VDAC2_MOUSE	W**C**EYGLTFTEK	2	SPM	76-86	SPM	77		
VDAC2_MOUSE	WNTDNTLGTEIAIEDQI**C**QGLK	18	SPM	87-108	SPM	104		
VISL1_MOUSE	EFI**C**ALSITSR	4*	Cytosolic	84-94		87		
VISL1_MOUSE	SDPSIVLLLQ**C**DIQK	11	Cytosolic	177-191		187		
