# Supplementary figures and images for: Identification of substrates of palmitoyl protein thioesterase 1 highlights roles of depalmitoylation in disulfide bond formation and synaptic function
Source: PLoS Biol. 2022 Mar 31;20(3):e3001590. doi: 10.1371/journal.pbio.3001590 (PMC9004782; doi:10.1371/journal.pbio.3001590)

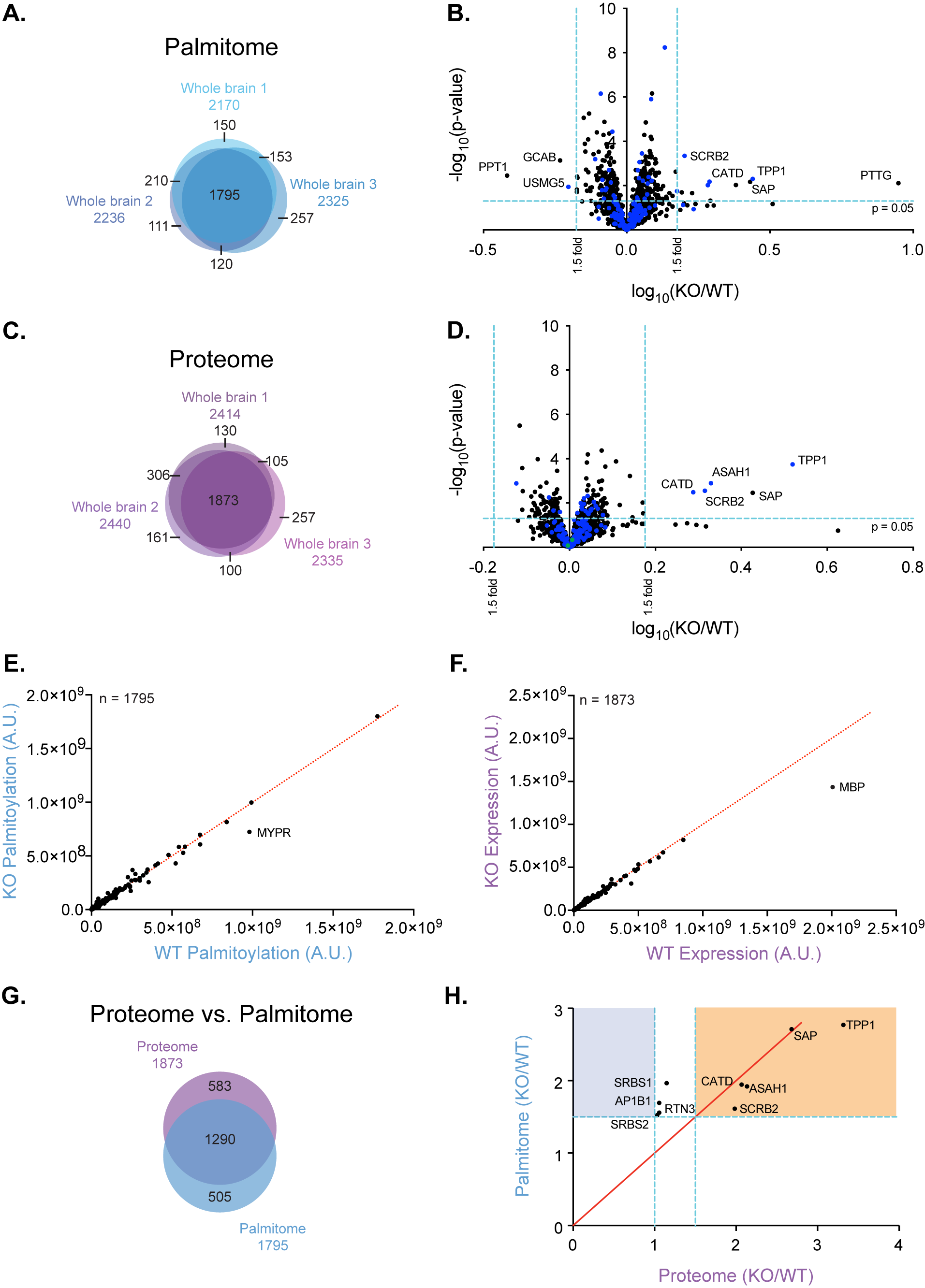

Supplement: S1 Fig — (A) Venn diagram of 3 independent palmitome experiments exhibits 1,795 common proteins. (B) Volcano plot of fold change between genotypes (PPT1 KO/WT) for common proteins with putative synaptic PPT1 substrates in blue (Fig 2B, S2 Table, S1 Data). A total of 15 palmitoylated proteins are significantly differentially expressed in whole brain (3 decreased, including PPT1; 12 increased; 1.5-fold, p < 0.05; blue lines). (C) Venn diagram of 3 independent proteome experiments exhibits 1,873 common proteins. (D) Volcano plot of fold change between genotypes (KO/WT) for common proteins with putative synaptic PPT1 substrates in blue (Fig 2B, S2 Table, S1 Data). A total of 5 proteins are significantly up-regulated (1.5-fold, p < 0.05; blue lines). Other depalmitoylating enzymes (green points) do not display compensatory up-regulation of protein expression. (E) Palmitoylated protein expression was highly correlated between genotypes for palmitome hits (m = 0.9753 ± 0.0030; R2 = 0.9857; S1 Data). (F) Protein expression was highly correlated between genotypes for proteome hits (m = 0.8675 ± 0.0037; R2 = 0.7711). When the MBP outlier was removed, this correlation was improved (m = 0.9802 ± 0.0023; R2 = 0.9916; S1 Data). Red lines indicate 1:1 WT to PPT1 KO protein expression ratio. (G) Venn diagram of 1,290 proteins common between whole brain proteome and palmitome (n = 3 each). (H) Protein expression levels compared to palmitoylation levels for significantly changed proteins in palmitome. Proteins in orange region are significantly increased (1.5-fold; p < 0.05) in both the proteome and palmitome. Proteins in blue region display decreased or unchanged protein expression and increased palmitoylation. Red line indicates equal expression and palmitoylation levels (x = y; S1 Data). KO, knockout; PPT1, palmitoyl protein thioesterase 1; WT, wild-type. (TIF) [file pbio.3001590.s007.tif]

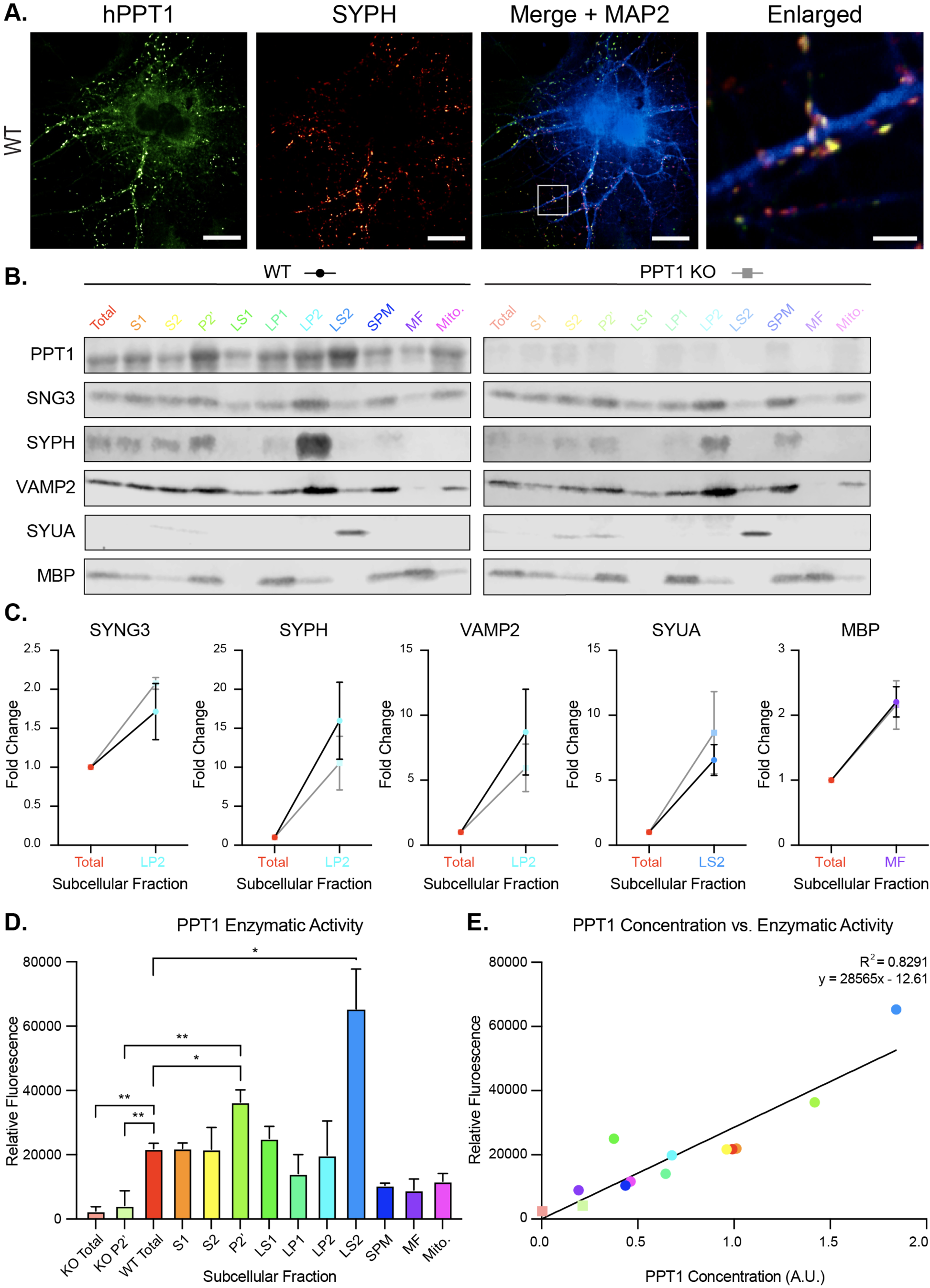

Supplement: S2 Fig — (A) hPPT1 (green) colocalizes with synaptophysin 1 (SYPH; red) in MAP2+ neurites in WT mouse primary neuronal culture (50 μm scale bars; 10 μm scale bar in higher magnification image). Lentiviral transduction of human PPT1 was performed due to lack of commercially available antibodies that can detect mouse PPT1 by ICC (including the custom antibody used for western blotting). (B) Endogenous PPT1 is present in all synaptic fractions of a subcellular fractionation of whole brains (8 WT and 8 PPT1 KO mice; age = 2 months; run in biological triplicate) with the highest level in synaptic cytosol (LS2). (C) Immunoblot quantifications of 3 subcellular fractionation experiments (S1 Data). Markers of synaptic subcompartments are appropriately localized and enriched (fold change from total) for both genotypes (WT, red; PPT1 KO, black), with no difference between genotypes: SNG3, synaptophysin 1 (SYPH), and synaptobrevin 2 (VAMP2) in synaptic vesicle enriched fraction (LP2); α-synuclein (SYUA) in synaptic cytosolic fraction (LS2); MBP in MF. (D) WT synaptic subcompartment fractions display significant PPT1 enzymatic activity, most notably the synaptic cytosol (LS2). Negligible enzymatic activity was detected in PPT1 KO total and P2’ fractions shown for comparison. Bars represent the mean of 3 technical and 3 biological replicates normalized to substrate-free controls, with SEM error bars (* p < 0.05; ** p < 0.01; S1 Data). (E) Average PPT1 protein concentration determined by quantitative immunoblotting (B) is highly correlated with average PPT1 enzymatic activity (D) (y = 28565x – 12.61; R2 = 0.8291; S1 Data). ICC, immunocytochemistry; KO, knockout; LS1, LP1, crude synaptic cytosol and membrane; LP2, synaptic vesicles; LS2, synaptic cytosol; MBP, myelin basic protein; MF, myelin fraction; Mito, mitochondria; P2’, crude synaptosomes; PPT1, palmitoyl protein thioesterase 1; S1, first supernatant; S2, second supernatant; SNG3, synaptogyrin 3; SPM, synaptic plasma membrane; WT, wi [file pbio.3001590.s008.tif]

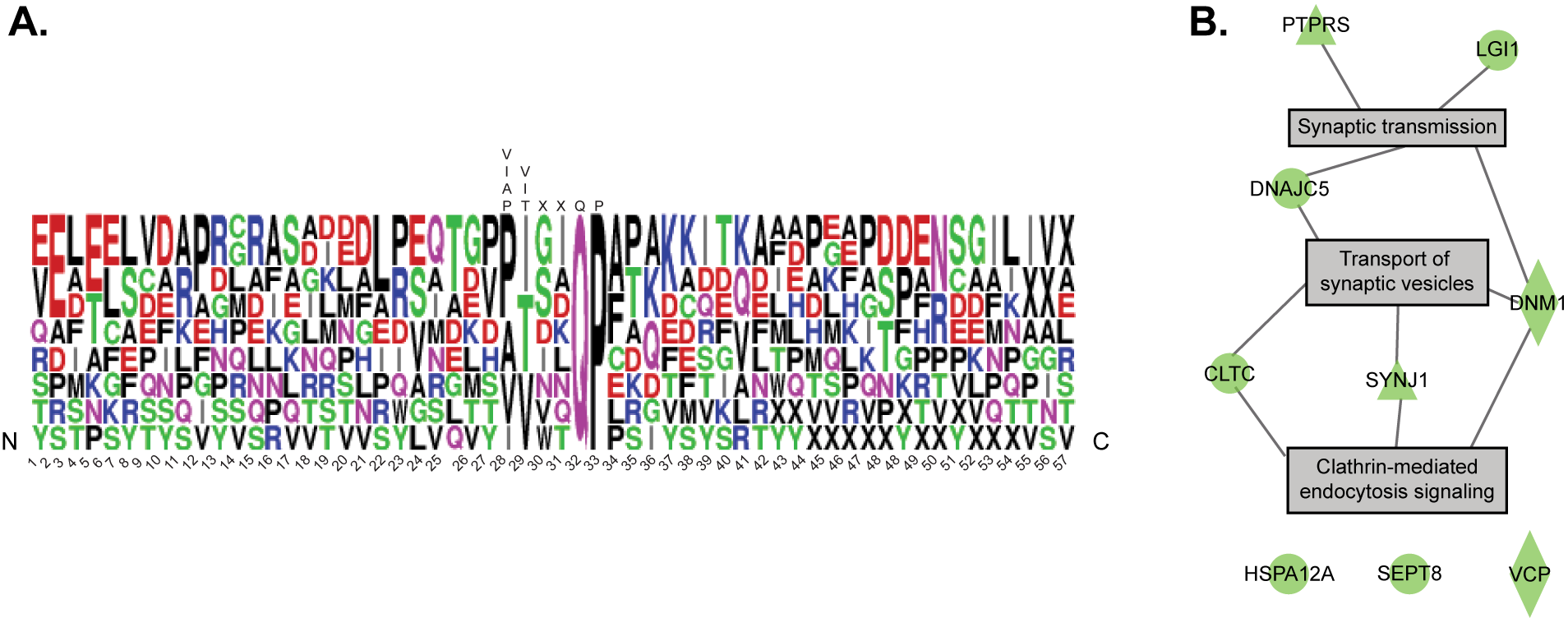

Supplement: S3 Fig — (A) Alignment of DHHC17 recognition motif for 9 endocytic PPT1 substrates with 26 amino acids on either side. The motif is noted above the logo plot. There are no other motifs surrounding the DHHC17 motif. (B) IPA of DHHC17 motif-containing proteins identifies clathrin-mediated endocytosis signaling as the top enriched pathway. These pathways account for 6 of the 9 motif-containing proteins. PPT1, palmitoyl protein thioesterase 1. (TIF) [file pbio.3001590.s009.tif]
