# Supplementary material for: Identification of substrates of palmitoyl protein thioesterase 1 highlights roles of depalmitoylation in disulfide bond formation and synaptic function
Source: PLoS Biol. 2022 Mar 31;20(3):e3001590. doi: 10.1371/journal.pbio.3001590 (PMC9004782; doi:10.1371/journal.pbio.3001590)
Supplement: Attachment — Submitted filename: Response to Reviewers.pdf [file pbio.3001590.s010.pdf]

RESPONSE TO REVIEWERS

We thank all the Reviewers for their consideration and careful review of our manuscript "Identification of PPT1 substrates highlights roles of depalmitoylation in disulfide bond formation and synaptic function" as a Methods and Resources article. We appreciate the constructive comments and experimental suggestions. We are especially grateful that all Reviewers felt that our manuscript would be a useful resource to the scientific community. We are also thankful that several Reviewers highlighted the importance of the work. We have addressed the Reviewers' comments in a point-by-point manner below, in italics.

Reviewer remarks:

Reviewer #1:

Gorenberg et al performed the acyl-RAC assay to systematically identify S-palmitoylated proteins from wild-type and PPT1 knockout mouse brains. They identified and validated PPT1 substrates in the brain, which include CSPa, Goa, NRCAM, CADM2, GluA1 and so on. Furthermore, the authors propose that depalmitoylation of transmembrane PPT1 substrates regulates their disulfide bond formation. Thus, this paper first provided a useful resource of global palmitoylated substrates of PPT1.

We thank the Reviewer for this evaluation of our manuscript as a useful resource that expands the repertoire of known palmitoylated proteins and PPT1 substrates.

Addressing the following points would strengthen this paper.

1. In Fig. 1C, SNAP-25 levels seems similar between +hydroxylamine (HA) and -HA samples. Because SNAP-25 is a representative palmitoylated protein, I am concerned about the specificity of the acyl-RAC assay.

Acyl-RAC is a well-documented method to purify palmitoylated proteins and has been used in numerous studies (Henderson et al., Acta Neuropathol, 131, 2016; Wan et al., Chem. Biol. 20, 2013; Forrester et al., J. Lipid Res. 52, 2011). Indeed, by cross comparisons with CCS-Palm and Swiss Palm, 100% of our high confidence PPT1 substrate hits and 94% of our medium confidence hits were previously identified as palmitoylated proteins (Tables 1 & 2) strongly suggesting specificity. Furthermore, endophilin and actin, two non-palmitoylated proteins, did not purify on the Thiopropyl Sepharose beads, indicating that this assay allows us to selectively isolate palmitoylated proteins. Regarding SNAP-25, SNARES in general are notorious for being 'sticky' proteins. We agree that SNAP-25 does not show a large difference between the +HA/-HA lanes unlike CSPα. This is one of the reasons why we chose only to compare KO+HA/WT+HA by setting stringent criteria (cut off: 1.5 fold and must be found in 9 replicates) to rule out any non-specific interactions. By our criteria, SNAP-25 is not a PPT1 substrate, supporting our assay design.

2. Identified cysteine depalmitoylation sites by PPT1 include extracellular cysteines (Figs. 3E and 4A) and cytosolic ones (e.g, CSPa, Goa, dynamin-1), suggesting that PPT1 acts at the lumen side of ER and cytosol side. To clarify it, the authors should show the endogenous PPT1 localization using knockout-validated antibody or tagged knock-in approaches like SLENDR (Mikuni et al, Cell 2016) and ORANGE (Willems et al, PLOS BIOL 2020).

We agree with the Reviewer that our data does suggest that PPT1 acts both in the secretory pathway and in the cytosol. We originally tried to answer this question by immunostaining.

*However, the lack of knockout-validated commercially available antibodies for mouse PPT1 makes these experiments to confirm the endogenous PPT1 localization difficult and has hindered the field as a whole. In attempts to immunostain for PPT1, we tested 10 commercial antibodies (Abgent (AP2538b), Abgent (AP2538a), Aviva (OAAB15942), Thermo (PA5-12228), LSBio (LS-C314992), LSBio(LS-C749749), Santa Cruz (SC-21258), GeneTex (GTX110677), ProteinTech (10887-1-AP), Bioss (bs-6619R)). Frustratingly, they all had substantial non-specific reactivity or no signal. Having exhausted commercial options, we generated 2 antibodies against mouse PPT1, affinity purified them, and confirmed their specificity using PPT1 KO samples. We then blotted synaptic fractions (**Rebuttal Figure 1/Fig. S2B**) and showed PPT1 is indeed detectable in the synaptic cytosol as well as the synaptic vesicle fraction (LS2), suggesting that it may act on both sides of the secretory pathway. Unfortunately, our efforts to get our custom antibody to work in ICC or IHC applications failed.*

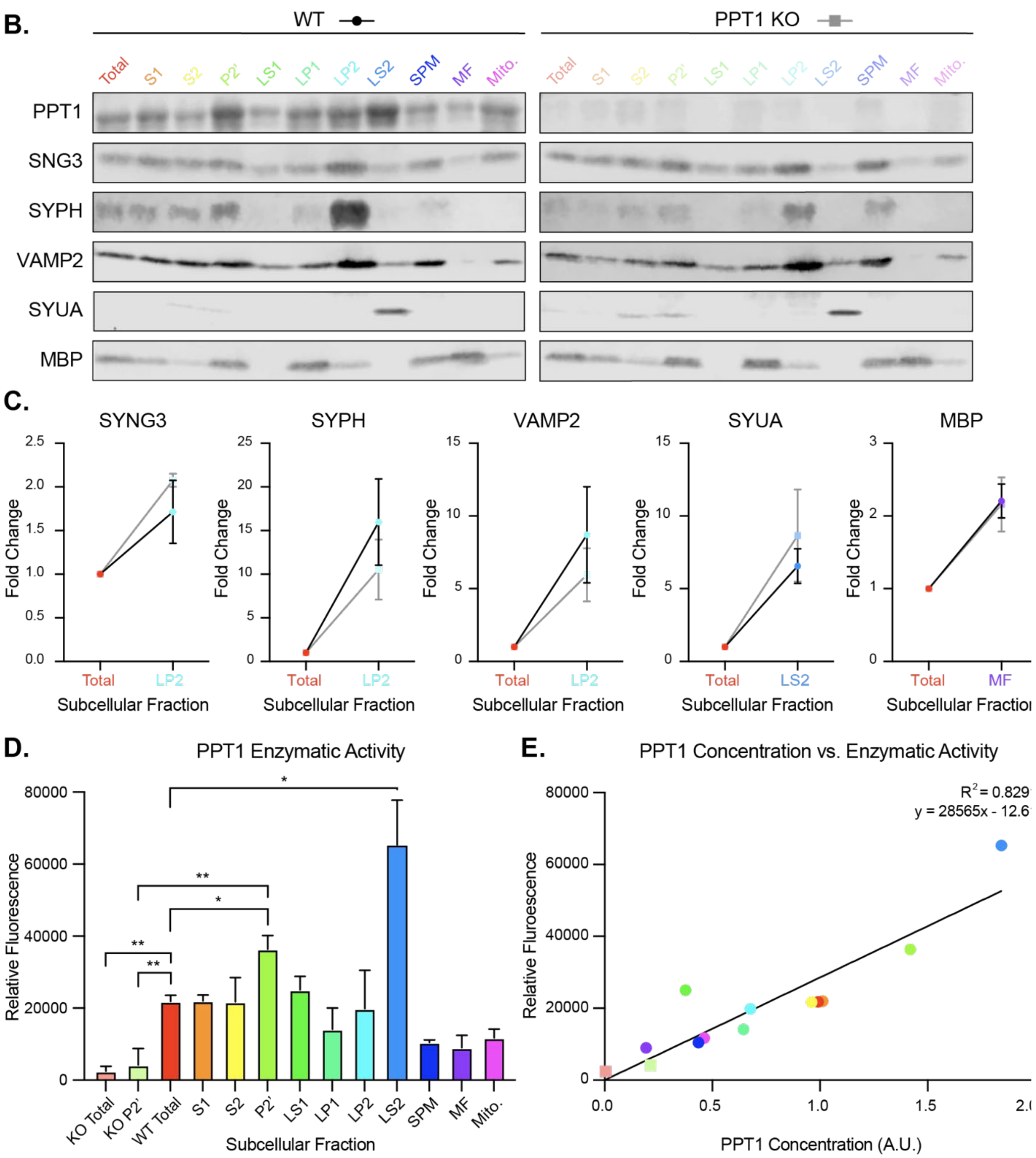

Rebuttal Figure 1 (Supplemental Fig. S2B-E): *B) PPT1 is present in WT samples and absent in PPT1 KO. PPT1 is found in all subcellular fractions but enriched in synaptosomes (P2') and synaptic cytosol (LS2) C) Subcellular fractions are enriched for markers of fraction purity. D-E) PPT1 enzymatic activity, correlating with PPT1 protein expression, is detected in subcellular fractions, and is enriched in synaptosomes (P2') and synaptic cytosol (LS2) compared to the whole brain (WT total) and PPT1 KO. WT total activity is significantly higher than KO total and P2' ** p<0.01. PPT1 activity in synaptosomes (P2') is significantly higher than KO P2' (** p<0.01) and WT total (* p<0.05). PPT1 activity is enriched in WT synaptic cytosol (LS2) compared to WT total homogenate (* p<0.05).*

*In response to Reviewer #2, we performed a PPT1 enzymatic activity assay (Van Diggelen, O.P. et al. Mol Genet Metab 66, 240-244, 1999) and show that PPT1 is indeed active in synaptic cytosol and synaptic vesicle fractions (**Rebuttal Figure 1/New Fig. S2D**). Furthermore, we demonstrate that PPT1 activity corresponds to protein levels determined by quantitative immunoblotting with our custom antibody (**Rebuttal Figure 1/New Fig. S2E**).*

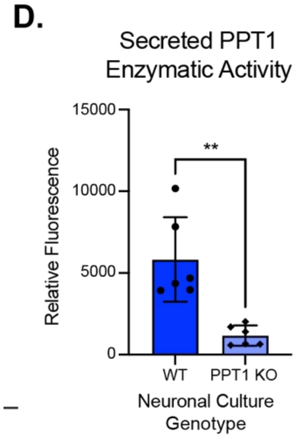

Rebuttal Figure 2 (Fig. 4D): D) Activity of PPT1 enzyme measured from filtered WT or PPT1 KO primary neuronal culture medium.

*We also demonstrated that secreted PPT1 enzyme activity is detectable in the filtered medium of WT primary neuronal cultures and absent in PPT1 KO cultures (**Rebuttal Figure 2/New Fig. 4D**), supporting our finding of both extracellular and cytosolic PPT1 depalmitoylation sites. We agree that the next steps to further characterize the subcellular location of PPT1 will involve CRISPR based knock-in strategies such as SLENDR and ORANGE, but the time and expertise required for these experiments is substantial and is beyond the scope of the current manuscript.*

3. Figure numbers of Fig. S2 are not correct. Please carefully check the manuscript.

We apologize for this oversight and have carefully checked figure references to amend this error.

<u>Reviewer #2:</u>

Infantile neuronal ceroid lipofuscinosis (INCL) is a uniformly fatal neurodegenerative lysosomal storage disease (LSD) caused by inactivating mutations in the CLN1 gene. CLN1 encodes palmitoyl-protein thioesterase-1 (PPT1), a lysosomal depalmitoylating enzyme. It has been proposed that PPT1-deficiency leads to lysosomal accumulation of S-palmitoylated proteins (constituents of ceroid) leading to INCL pathogenesis. Despite the discovery in 1995, that inactivating mutations in the CLN1 gene cause INCL, the substrates of PPT1 that accumulate in the lysosome and other organelles have remained unidentified, not for a lack of trying. In this manuscript, Gorenberg and colleagues used mass spectrometric analyses of proteins in synaptosomes purified from the brain tissues of WT and Cln1-/- mice and identify the putative substrates of PPT1. The identification of the substrates of this enzyme may unravel the pathogenic mechanism(s) underlying INCL and would be a major advance in this area of research. From this standpoint, the study of Gorenberg and colleagues attempt to provide important information, which, if validated, may help us to understand the mechanism of this devastating disease. While at the outset it appears to be an important study, some important questions need to be addressed.

We are thankful that the Reviewer recognizes the importance of our findings and states that identification of the substrates of PPT1 would be a major advance that may help us understand the mechanism of CLN1. We are appreciative of the Reviewer's careful reading of the manuscript and experimental suggestions.

Major points-

A. Using unbiased proteomic approaches, the authors claim to have identified 9 distinct classes of PPT1 substrates in synaptosomes purified from WT and Cln1-/- mouse brain. They show that Ppt1-deficiency in Cln1-/- mouse brain causes these substrates to accumulate in synaptosomes. Notably, the authors chose to use purified synaptosomes from the brain of 129 Cln1-/- mice and to identify the proteins accumulated in synaptosomes from the brain of these mice. Since genetic background may influence gene expression, it is prudent to use mice with identical genetic background. This is the reason why many investigators have first generated 129 Cln1-/- mice because the ES cells used to target the Cln1 gene was derived from 129 mice (See Gupta et al. PNAS 2001). In subsequent studies, the genetic background was converted to C57 by backcrossing 129 Cln1-/- mice with C57 WT mice >10 times (see the Methods in Dearborn, J.T. et al. Sci Rep. 5, 12752, 2015). The WT littermates from C57 Cln1-/- mice provided a homogeneous C57 genetic background. Alternatively, the authors could have used purified synaptosomes from 129 Cln1-/- and their WT littermates which would have obviated the time-consuming backcrosses to obtain C57 genetic background for both WT and KO mice. This way, the results of their proteomic studies comparing the putative Ppt1-substrates in synaptosomal preparations would have been on a solid ground.

We apologize for not clearly stating the background of our PPT1 KO mice and the specific definition of "WT" for mice used in our experiments in the Methods section. We ensured that the experiments were controlled for genetic background. As noted by the Reviewer, we obtained the PPT1 KO mice (B6;129- Ppt1^{tm1Hof}/J) *from the Jackson Laboratory, which maintains this strain on a C57Bl/6;129S6 background. Since obtaining the PPT1 KO strain, we have performed 6 or more backcrosses to wild type C57Bl/6 mice and have selected only black mice for breeding. All mice in our colony have been black for several years, suggesting >95% C57Bl/6 background. We sent tail DNA of mice used for proteomics experiments to JAX for an independent quantification of the exact percentage of C57Bl/6 genetic background. JAX quantified the genetic background of the mice used in experiments in this study to be 98.66 to 99% C57Bl/6 (**Rebuttal Figure 3**). Overall, the C57Bl/6 is an appropriate control for these mice. More importantly, our primary synaptosome screen, which determined our list of putative PPT1 substrates, was performed using only WT and PPT1 KO littermate mice (from heterozygous PPT1 +/- crossings) as suggested by the Reviewer. We regret this small but critical omission and have made modifications to ensure that this information is accurately described in the text.*

SNP Genome Scanning Analysis Report

Project Info:

Project Number:	133423
SNP Panel:	Fixed
PI Name:	Sreeganga Chandra
PI Institution:	Yale University
Strain Name and ID:	

Backgrounds Present:	C57BL/6J x 129S1/SvImJ

Result summary:

Sample	129S1/SvImJ	C57BL/6J
1 761340	INC	INC
2 761340	1.01%	98.99%
3 761340	1.33%	98.67%
4 761340	1.34%	98.66%
B6J	0.00%	100.00%
129S	100.00%	0.00%
HET	50.00%	50.00%
NTC		

Rebuttal Figure 3: *SNP Genome Scanning Analysis Report. Independent quantification of C57BL/6J and 129S1/SvImJ genetic background by JAX. Sample 1: WT littermate of PPT1 KO used for synaptosome proteome - data not collected due to DNA quality; INC, inconclusive. Sample 2: Mouse used for whole brain proteome. Sample 3: Mouse used for disulfide bond tertiary screen proteome. Sample 4: Littermate from mouse used for second disulfide bond tertiary screen proteome replicate.*

B. The main organelle in which PPT1 (CLN1 gene product) is localized in cells has been clearly established to be the lysosome (Verkruyse and Hofmann. JBC. 271,15831-15836, 1996; Hellsten et al. EMBO J. 15,5240-5245, 1996) although trace amounts of PPT1 have also been reported in extra lysosomal sites, like the synaptosomes. This may be because a small % of the soluble lysosomal proteins are known to be secreted instead of being targeted to the lysosome (Ballabio & Gieselmann. Biochim Biophys Acta. 1793, 684-696, 2009). Moreover, the major pathological features of INCL include the accumulation of S-palmitoylated (S-acylated) proteins in the lysosome. Further, the S-acylated proteins are the major constituents of ceroid (called granular osmiophilic deposits or GRODS) (Galvin, N. et al. Pediatr Dev Pathol. 11, 185-192, 2008). This is a characteristic pathological finding in the brain of INCL patients and in that of the Cln1-/- mice.

*The Reviewer correctly points out that that PPT1 is found in lysosomes in non-neuronal cells, but the localization in neurons is more complex. There appear to be two pools, one that is trafficked by the secretory pathway to lysosomes or secreted, and one in the synaptic cytosol (**Rebuttal Figure 1/Fig. S2B**). In neurons, PPT1 is enriched in synapses (**Rebuttal Fig. 1/Fig. S2B**); the synaptic localization of PPT1 has been established by many groups (*Lehtovirta *et al. Hum. Mol. Gen 2001; Ahtianen et al., J. Comp. Neurol. 2003; Kim et al., J. Clin. Invest. 2008; Sapir et al., Front Cell Neurosci 13, 2019). Both our ICC data (Fig. S2A) and our palmitoylation data (Fig 2B) bear out the presence of PPT1 at the synapse. Further, in response to the Reviewer, we have now confirmed that PPT1 enzymatic activity is indeed enriched at synapses (**Rebuttal Fig. 1/New Fig. S2D**). It is well known that synapses, and presynaptic termini in particular, are devoid of mature lysosomes.*

We concur that the major pathological feature of INCL is accumulation of palmitoylated proteins in lipofuscin. This does not necessarily mean that a solely lysosomal localization of the mutated

gene product is a requisite for lipofuscin accumulation. For example, the NCL protein CLN4 encodes the synaptic vesicle protein CSPα, and CLN6 and CLN8 are ER proteins.

B (cont.). Furthermore, it has been reported that the S-palmitoylated proteins require depalmitoylation for their degradation by lysosomal acid hydrolases (Lu, J.Y. & Hofmann, S.L. J Lipid Res. 47, 1352-1357, 2006). The authors' suggestion that "Protein degradation does not require depalmitoylation by PPT1" is premature. An explanation is needed as to why intravenous administration of high-dose PPT1-enzyme to Cln1-/- mice reduces lysosomal storage of S-palmitoylated proteins and modestly prolongs survival in a preclinical mouse model of INCL (Hu, J. et al. Mol Genet Metab. 107, 213-221, 2012).

*The papers from the Hofmann lab do hypothesize that depalmitoylation is required for degradation of proteins. However, they did not identify the substrates for which this rule applies. It is presently dogma in the field that this a universal rule, that depalmitoylation is obligatory for degradation of all palmitoylated proteins. As we show in Fig. 2H, for the vast majority of PPT1 substrates, depalmitoylation is not needed for protein degradation in young mice (when there is no lipofuscin). Only 4 proteins appear to perhaps show depalmitoylation-dependent degradation: ASAH1, CATD, SCRB2, and TPP1, which each have links to lysosomal storage diseases. As the protein constituents of INCL lipofuscin are unknown (besides saposin, SAP), we do not know if lipofuscin contains thousands of palmitoylated proteins or just a few. Proteomic examinations of age-related neuronal and retinal lipofuscin have also identified ASAH1, CATD, SCRB2 and TPP1 (**Rebuttal Figure 4**), and various PPT1 substrates, supporting our tenet. It should be noted that with age, when lysosomal degradation is compromised, the degradation of many proteins will be impacted, not just PPT1 substrates (see Sleat et al., 2019). Thus, our hypothesis does not preclude that depalmitoylation is required for substrate degradation at more progressive stages of disease, when there is substantial lipofuscin accumulation.*

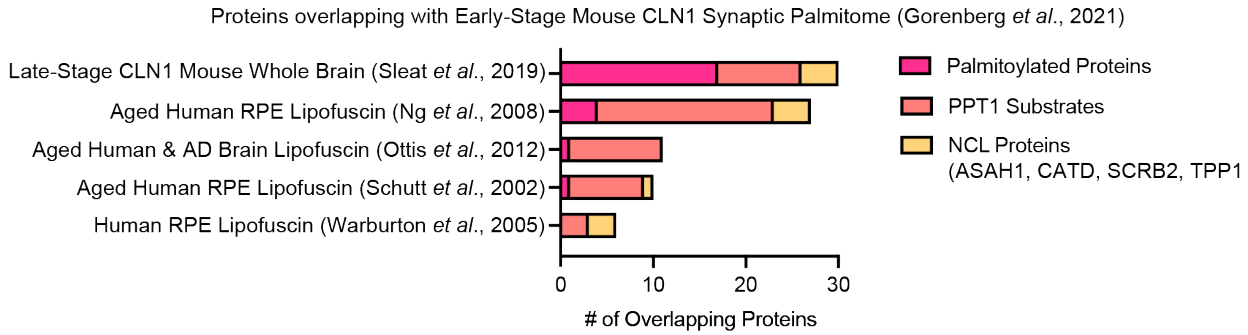

Rebuttal Figure 4: *Validated PPT1 substrates, palmitoylated proteins identified by our synaptosome Acyl RAC screen, and NCL-related proteins are found in proteomic datasets of late-stage CLN1 disease and purified human lipofuscin.*

PPT1 enzyme replacement therapy does decrease lipofuscin in Hu, J. et al. Mol Genet Metab. 107, 213-221, 2012, but only peripherally in liver and spleen. Furthermore, ERT had no effect on spinal cord or retinal autofluorescent storage material. We plan to investigate the protein component of NCL-related lipofuscin in subsequent studies and will also explore whether lipofuscin pathology is altered by modulation of ASAH1, CATD, SCRB2 and TPP1. For now, to be more precise, we have tempered our statement "Protein degradation does not require depalmitoylation by PPT1" to "Protein depalmitoylation is not required for the degradation of most PPT1 substrates in early CLN1 disease".

C. Since Ppt1 functions in an acidic environment of the lysosome, it would be important to present data showing: (i) the pH within the synaptosomes of WT and Cln1-/- mice and (ii) whether Ppt1 is enzymatically active in the synaptosomes from WT mice? In this regard, a very good assay is commercially available to evaluate the enzymatic activity of PPT1 (Van Diggelen, O.P. et al. Mol Genet Metab 66, 240-244, 1999).

The pH sensitivity of PPT1 is broad and recombinant PPT1 functions optimally at neutral pH (Verkruyse & Hofmann, J Biol Chem 271, 1996). Our own data also supports this finding that PPT1 functions at a neutral pH (Henderson et al., Acta Neuropathol., 2015).

*We thank the Reviewer for the experimental suggestion to measure PPT1 enzymatic activity in synaptosomes. We have successfully carried out this experiment and show that PPT1 is indeed enzymatically active in synaptosomes from WT mice (**Rebuttal Figure 1/New Figure S2D-E**). Using PPT1 KO synaptosomes, we confirm the specificity of the enzymatic activity.*

C. (cont.). Do S-palmitoylated proteins accumulate in the synaptosomes of Cln1-/- mice? The reason for determining PPT1 enzymatic activity in synaptosomes is that if in WT synaptosomes PPT1 activity cannot be detected then the identification of putative PPT1 substrates in synaptosomes may not have any relevance to the disease.

The accumulation of S-palmitoylated proteins in PPT1 KO synaptosomes is the basis of our primary screen, and these data are shown in Fig. 2B. Also, see our above point concerning the detection of PPT1 enzyme activity in WT and absence in PPT1 KO synaptosomes.

C. (cont). Since most of the PPT1 is localized in the lysosome and the method for purification of lysosomes from brain tissues is straight forward, the authors could identify the substrates of this enzyme using purified lysosomes. If the authors can confirm that the same 9 distinct classes of Ppt1-substrates, which they identified in the synaptosomes, also accumulate in the lysosomes from Cln1-/- mice, their proteomic data will be much more solid.

*In neurons, lysosomes are mainly found in soma, while endosomes and autophagosomes are found in synapses. Lysosomal purification will end up isolating most acidic organelles (endosomes, autophagosomes and lysosomes) of both neurons and glia. Furthermore, as Cln-/- lysosomes have an improper pH (Bagh et al., Nat. Commun. 8, 2016) and are enlarged (Sima et al., Orphanet J Rare Dis 13, 2018), they do not float at the same density as WT lysosomes in sucrose gradients, precluding accurate comparisons. Therefore, we cannot perform the proposed experiment. Instead, we compared our synaptic palmitome to several published proteomes of purified lipofuscin and late-stage NCL whole mouse brain (see **Rebuttal Figure 4** in response to comment 2B). PPT1 substrates were identified in these datasets and fall into 7 of the 9 UniProt classes we identified (**Rebuttal Figure 5**). ASAH1, CATD, SCRB2, and TPP1, the proteins we found to accumulate in both whole brains and synaptosomes, were also upregulated in most of these datasets. We believe these comparisons emphasize the disease relevance of our findings and suggest that PPT1 substrates accumulate with NCL disease progression.*

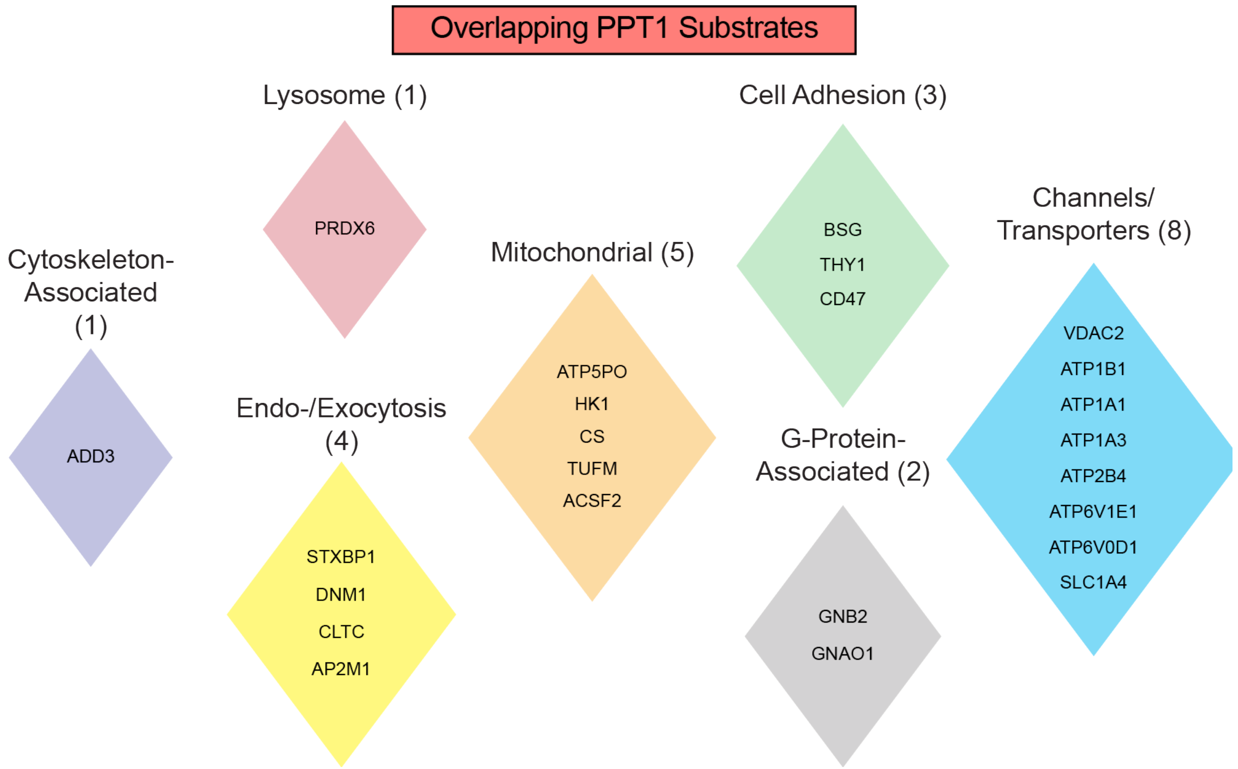

Rebuttal Figure 5: *The PPT1 substrates identified as overlapping with purified lipofuscin and late-stage CLN1 datasets (see Rebuttal Figure 4) are not solely lysosomal but participate in 7 of the 9 UniProt classes originally identified (Figure 3C).*

D. In the methods section of the manuscript, I could not find how the authors determined the purity of the synaptosome preparations? Purification of the intracellular organelles is difficult, especially from the brain and the authors should describe the methodology is detail.

*Purification of synaptosomes is a standard and well-established method (Huttner et al., 1983). We have previously done electron microscopy on these preparations and have shown that they mainly contain synaptic termini (see Vargas et al., 2017; Cell Reports). We have also shown that they are functional, i.e. release neurotransmitter when stimulated (Vargas et al., 2017; Cell Reports; Vargas et al., BioRxiv 2020). Furthermore, by western blotting of our synaptosome preparations, we observe enrichment of well-established synaptic markers, which is a standard method for monitoring purity. To demonstrate that we enrich for synaptic proteins and that there is no difference in WT and PPT1 KO synaptosomal preparations, we performed quantitative immunoblotting and added this information to the manuscript (**Rebuttal Figure 1/New Fig. S2C**). We have also edited the Methods and figure legends to reflect this information.*

E. The authors claim that they have identified ">100 novel PPT1 substrates". To confirm this finding, they used in vitro assays using recombinant PPT1 to demonstrate that those proteins are the substrates of Ppt1. This assumption may not be totally correct. For example, in in vitro assays the H-Ras protein has been reported to be depalmitoylated by PPT1 (Lu, J.Y. & Hofmann, S.L. J Biol Chem. 270,7251-7256,1995), whereas the enzyme that catalyzes the depalmitoylation of H-Ras in vivo is a cytosolic thioesterase, acyl-protein thioesterase-1 (APT1) (Duncan & Gilman J Biol Chem. 273,15830-7, 1998). For this reason alone, the authors could have used the proteins from purified lysosomes from Cln1-/- mouse brain and those from their WT littermates to authenticate the results from the synaptosomes.

We agree relying solely on in vitro data may lead to false positives. Therefore, we used in vivo increases in palmitoylation as the primary criterion to identify putative PPT1 substrates, then validated them with the in vitro depalmitoylation assay. The argument presented by the Reviewer was our reasoning for not categorizing "residual" proteins only identified in the second in vitro screen as PPT1 substrates. In support of our stringent two-step screen design, we did not identify H-Ras as a PPT1 substrate. Regarding purifying lysosomes from Cln-/- brains, refer to our response to point C.

F. Auto acylation (auto palmitoylation) and depalmitoylation may occur spontaneously without palmitoyl acyltransferases (called ZDHHCs) and palmitoyl-protein thioesterases, respectively. It has been suggested that auto acylation plays important roles in the dynamic thioesterification of some cellular proteins like the G Protein a-subunits (Duncan & Gilman JBC 271, 23594-23600, 1996). During the rigorous procedure of synaptosome isolation and purification some proteins may undergo auto acylation when under in vivo conditions non-enzymatic palmitoylation-depalmitoylation may occur spontaneously, albeit at a very low level. Furthermore, constitutive deacylation/reacylation cycle operates on S-palmitoylated proteins. Thus, just by comparing the level of S-palmitoylated proteins in synaptosome preparations from WT and Cln1-/- mice may not identify the specific substrates of PPT1.

Auto-palmitoylation, if it does indeed occur, is a slow process and requires elevated temperatures. To minimize such effects, we carry out all synaptosomal preparations rapidly and at 4°C. Furthermore, non-enzymatic catalyzed auto-acylation and -depalmitoylation are likely to be the same in both genotypes (WT and PPT1 KO). Our comparisons of relative palmitoylation levels between genotypes will thus normalize this baseline auto-catalysis, and proteins exhibiting such effects will not be identified in our primary KO/WT screen. Furthermore, we went beyond comparing the level of S-palmitoylated proteins in synaptosome preparations to identify specific substrates of PPT1, as we performed a secondary screen with direct PPT1-mediated depalmitoylation to validate our putative hits.

G. The authors claim that the "Identification of PPT1 substrates highlight roles of depalmitoylation in disulfide bond formation and synaptic function". Although it is a novel idea, I fail to understand the rationale and the validity of this statement. Disulfide bond formation in vivo primarily occurs within the endoplasmic reticulum (ER). It is catalyzed by a variety of oxidoreductases, including the members of the protein disulfide isomerase (PDI) family (Bechtel et al. ACS Chem Biol. 15, 543-553, 2020). In an oxidative environment of the in vitro experiments, it would be extremely difficult to prove that depalmitoylation promotes disulfide bond formation. It may be possible to validate this prediction using an assay system in which the whole process is performed under stringent (oxygen-free) nitrogen atmosphere so that the proteins do not have any contact with oxygen. The "Identification of PPT1 substrates highlight roles of depalmitoylation in disulfide bond formation and synaptic function" is of enormous importance in thiol biochemistry. However, it is critical that the authors provide solid evidence in support of this prediction.

Our discovery that depalmitoylation coincides with disulfide bond formation is novel and we agree that it is potentially of enormous importance in thiol biochemistry. We will carry out detailed studies on this topic in the future using select proteins. We have revisited our wording of this finding to ensure we do not overstate it; we emphasized that this is an early hypothesis and tempered suggestions that palmitoylation mediates disulfide bonding. Our findings are in line with new research that some disulfide bond formation may occur in the Golgi, especially for palmitoylated proteins (Betchel et al., ACS Chem. Biol. 2020). Further, the quaternary interactions of proteins may not occur until proteins have been trafficked to the appropriate destination (Biederer et al.,

Neuron 2017). Thus, the role of palmitoylation in disulfide bond formation may be involved in this post-ER process, as we suggest for the stabilization of IgG domains of synaptic adhesion molecules. Notably, the coincidence between disulfide bond formation and palmitoylation has been observed in the context of SOD1 maturation in ALS (Antinone et al., Sci Rep 7 2017). Replicating our synaptosome preparation and modified Acyl RAC assay under oxygen-free conditions is currently beyond our technical capabilities.

Minor points-

1. Throughout the manuscript, numerous references are cited inappropriately. This should be corrected.

We have checked all references and ensured they are cited appropriately.

2. In Figure 1A the schematic of Acyl RAC assay may not be necessary as it is a widely used assay method (Forrester et al. J. Lipid Res. 2010).

We believe the schematic of the Acyl RAC assay in Figure 1A is useful, as we make an atypical modification to this assay in Figure 5A which may otherwise be confusing for the non-expert reader.

<u>Reviewer #3:</u>

The authors used a relatively straightforward (compared to ABE capture) method, Acyl-Resin Assisted capture for selectively enriching previously palmitoylated proteins and subsequent mass spectrometry identifications. The method relies on alkylation of all free thiols by NEM , followed by cleaving of palmitate moiety by hydroxylamine, exposing free thiols , which are then selectively captured using thiol reactive beads. Captured proteins are then analysed by tryptic digestion and label-free quantitative mass spectrometry.

The authors used this method in an attempt to identifying substrates of PPT1 using PPT1 KO mouse model. The assumption here was proteins with increased palmitoylation in KO, as compared to wild type mouse, would be the targets for the PPT1. In parallel, a global proteome quantification is also performed in order to distinguish palmitoylation increase from protein expression level increase. While global protein and palmitome levels did not significantly altered in whole brain, the same experiment on enriched synaptosome revealed several significant changes. Authors follow up this data with additional filtering and comparison with other databases in order to validate this list of potential substrates of PPT1. While this list of potential substrates can be a resource for future targeted validation, the authors did not mention some of the major limitations of this Acyl-RAC workflow.

Although a number of proteome -wide palmitoylation studies have been performed recently by employing Acyl-RAC based purifications, this method is prone to false positives due to hydrolysis of the thioester bond of other cysteine modifications, such as nitrosylation and glutathionylation. Moreover, the method cannot distinguish other lipid adducts on proteins which also form thioester bonds with cysteine and once cleaved, free thiols, thereby may be co-purified with thiol reactive beads. Additionally, thioesters are common in active site cysteine of many proteins and therefore may be co-purified even if not palmitoylated. Therefore the presence of carbamidomethylated peptide in the Acyl-RAC purified proteins, still provide only an indirect evidence of (previously) palmitoylated proteins.

In spite of these major limitations to the workflow, authors try to systematically and methodically refine the list of palmitoylated proteins.

Acyl-RAC and ABE are the go-to methods for untargeted analysis of protein palmitoylation (Tewari et al., J. Vis. Exp. 2020; Edmonds et al., Sci. Rep. 2017; Forrester et al., J. Lipid. Res. 2011; Kang et al., Nature 2008; Wan et al., Nature Proc. 2007; Drisdel and Green, Biotech, 2004). We agree with the Reviewer's comments that the presence of carbamidomethylated peptides on their own do not always indicate palmitoylation. Therefore, we chose to compare PPT1 KO/WT ratios to rule out non-specific binding via cysteines. Only in the tertiary screen did we use the carbamidomethylated peptide data to infer relationships between depalmitoylation and disulfide bond formation. Here too, we used KO/WT ratios to infer palmitoylation and biological significance (See Figure 5G). In this more targeted interpretation of the data, we solely investigated stringently validated PPT1 substrates (i.e. AT1B2). Additionally, 100% of our high confidence PPT1 substrates were identified in the Swiss Palm database, which is a repository of palmitoylated proteins identified with AcylBiotinExchange, Acyl-RAC, or CLICK chemistry (Blanc et al., F1000Res *4, 2015). Many of these proteins were also identified as palmitoylated by ABE (Kang et al., Nature 456, 2008) and/or predicted to be palmitoylated using the CSS Palm bioinformatic tool (Ren et al., Protein Eng. Des. Sel. 21, 2008) (Table 1). We have edited the main text to include some of the caveats of Acyl-RAC (page 4, paragraph 2).*

However, there appears some inconsistency in the proteome and palmitome coverage in various mass spec experiments. Firstly, overall proteome coverage (of 1873 common proteins) in mouse brain both genotypes, is relatively low for the type of instrumentation used.

The proteome and palmitome coverage we achieved are likely due to the stringency of the criteria used to identify genuine hits (>2 peptides and Protein Score >100). Further, for various synaptosome preparations, detection of approximately ≥1000 proteins has precedence (Gulyássy et al., Amino Acids 52, 2020).

Secondly, while the first palmiotome screening of synapse identified 1378 proteins, the tertiary screening with dNEM identified in 3551 proteins in synapse and I wondered if there is any logical explanation for this. Probably protein sequence database search of MS data and preliminary filtering criteria have to be verified to prove these are genuine hits.

Data in the tertiary screen were analyzed at the peptide level rather than the protein level and replicate data were aggregated. Thus, this higher number of 3551 was not calculated in the same manner as the other screens. We apologize for this inconsistency and will include the modified number of total proteins detected so the screens are more easily comparable.

Currently data (PXD017270) on PRIDE is not accessible.

We apologize for this error. The accession and password for these data are provided here.
Project accession: PXD017270; **Username:** reviewer12457@ebi.ac.uk **Password:** RIV3vYAO
We will revisit our accessibility settings to ensure these data are publicly available, once published, as per PRIDE rules.

Reviewer #4:

This manuscript by Gorenberg et al describes very interesting new data regarding the potential substrates of palmitoyl protein thiosesterase 1. It has long been known that deficiency in this de-palmitoylating lysosomal hydrolase is the molecular cause of CLN1 disease, a fatal inherited and

profoundly neurodegenerative disorder of childhood. However, progress in understanding the pathogenesis of this disorder has been severely hampered by not knowing the normal substrates that PPT1 acts upon. This manuscript utilizes a novel methodological strategy combining Acyl Resin-Assisted Capture and mass spectrometry to identify these substrates. The new strategy is based on identifying proteins with increased palmitoylation in PPT1 deficient mouse brains, and then because other depalmitoylating enzymes exist, validating these targets via recombinant PPT1. This has revealed evidence that about 10% of palmitoylated proteins at the synapse appear to be PPT1 substrates. The authors have sorted these into nine separate classes that are related to the phenotypes of PPT1 deficient mice and CLN1 patients. There is also evidence that the depalmitoylation sites are most often cysteine residues in disulfide bonds, suggesting a role for PPT1 and palmitoylation in regulating such interactions.

These studies appear to have been conducted rigorously and are presented very clearly so that they are relatively simple to understand, even for a reader who is not a specialist in proteomic analysis. The figures are especially well presented with good use of color coding to present several complicated data sets. Scientifically, the manuscript is of considerable importance in presenting significant novel data about PPT1 substrates that has been lacking for some time. This has been made possible by the application of a new method that has resolved a problem the field has been facing for a long time. In this respect it will be of considerable interest not just for those studying this and similar disorders, but also more widely for studying the importance of palmitoylation at the synapse in a range of disorders. Nevertheless, there remain a few, mostly conceptual issues that the authors should address in a revised manuscript.

We thank the Reviewer for their assessment that our study was rigorously conducted, clearly presented, and will be of considerable importance to the field.

a) The authors conducted their study in PPT1 mice at 2 months of age, but no rationale for chosing this age is given. This age is relatively early in disease progression, and is a sensible choice over later stages when many more downstream changes might be evident. Please can the authors explain their rationale?

We chose to analyze PPT1 KO animals at 2 months for the very reasons listed by the Reviewer. An important consideration is that PPT1 KO mice do no accumulate lipofuscin or have overt neurodegeneration at this age. This allowed to us to ensure that there were no dramatic proteomic changes that would complicate interpretation of increases in palmitoylation, and thus identification of PPT1 substrates. We have now included our rationale for choosing this time point in the main text.

b) The study was performed using whole brain extracts. These will necessarily contain neurons in addition to different populations of glia (astrocytes, microglia, oligodendrocytes). How can the authors account this mixed cell population or control for this? Or is this not a complicating factor for the conclusions they have reached?

PPT1 KO microglia and astrocytes are dysfunctional in CLN1 disease and studies delineating their specific contributions to disease progression are underway (J. Lange et al., Acta Neuropathol Commun. 6, 2018). Although PPT1 KO affects glial cell populations, at this early disease timepoint we observed few changes to the whole brain proteome and palmitome, and thus conducted our subsequent screens with synaptosomes. Hence, we don't believe the contribution of glia is a complicating factor for the conclusions we have reached, but do not discount the potential importance of their contribution to disease progression.

c) Regionality is an important part of CLN1 pathogenesis, with markedly different onset and progression of pathology in the CLN1 brain and spinal cord. Have the authors considered including spinal cord samples in their analyses. Can the predict whether similar or different data may be produced?

This is an important suggestion, and we agree with the Reviewer that it may shed light on regional vulnerability in CLN1 pathogenesis. We conducted comparisons of our dataset to a recent proteomic analysis of PPT1 KO spinal cord by Nelvagal et al., Scientific Reports, 10 (2020). In 3-month PPT1 KO spinal cord, ASAH1 and TPP1 are also found to be upregulated, along with two of our validated PPT1 substrates, DCLK1 and HEXB, and a palmitoylated protein we also found to be upregulated in synaptosomes, MYO6. This indicates that the early proteomic changes we observed appear across the CNS. At the 7-month disease timepoint in this study, ASAH1 and TPP1 remain upregulated, and the number of accumulating PPT1 substrates that we identified expands to also include COTL1, DPP6, GNAI1, OAT, OXR1, PRDX6, PRRT3, SYNPR, SYT2, and VDAC2. An additional 10 palmitoylated proteins identified as upregulated in our screen are also upregulated in spinal cord at 7 months. The resource we have generated is thus a useful tool for interpreting CLN1 disease data to understand what observed effects may be a direct result of deficient depalmitoylation by PPT1. Further, these data underscore the relevance of our findings to CLN1 disease progression. We have cited this spinal cord data in the text.

d) Other depalmitoylating enzymes do not appear to be up regulated to compensate for lack of PPT1. Can the authors speculate further upon why this is the case? What does this tell us about the specificity of a depalmitoylating enzymes substrates?

Based on the few substrates identified for APTs and ABDHs, it does appear that there are distinct substrate repertoires for these depalmitoylating enzymes. This is clearly an incomplete picture and it is very possible that there is a subset of overlapping substrates. One possibility is that the different depalmitoylating enzymes are localized to different subcompartments of a cell, allowing for distinct substrates. Another is that different palmitoylation sites are regulated by different depalmitoylating enzymes, or that specificity is overlapping to facilitate greater combinatorial functionality. Our assay design can be easily adapted to APT and ABDH KO animals to find their substrates, and our study will be of utility to investigators studying these enzymes.

e) From a neuroscience perspective, most of the synaptic targets appear to be pre-synaptic rather than post synaptic. Can the authors speculate further on the functional or mechanistic basis of this selectivity? What implications does this have for PPT1 function or CLN1 disease pathogenesis?

*We also excitedly noted that most of the synaptic targets are pre-synaptic. This is consistent with previous findings and our own (Fig. S2) that PPT1 is axonally trafficked and localized (*Lehtovirta *et al. Hum. Mol. Gen 2001; Ahtianen et al., J. Comp. Neurol. 2003; Kim et al., J. Clin. Invest. 2008; Sapir et al., Front Cell Neurosci 13, 2019). We hypothesize that PPT1 is trafficked to the presynapse and acts along the way or at synapses to allow for functional maturation of presynaptic proteins or those exposed to the cleft. We speculate that CLN1 disease pathogenesis involves a 'dying-back' of neurons with axon retraction, like other neurodegenerative diseases such as Charcot-Marie Tooth disease. This assumption could be genetically tested by* Wld^{S} *crosses in the future.*

f) The authors predict that palmitoylated synaptic proteins traffic correctly in PPT1 mice, but may function poorly at the synapse due to absent or compromised depalmitoylation. This is a very

interesting suggestion. please can the authors expand upon the rationale for this, and what further evidence would be needed to prove this hypothesis?

We thank the Reviewer for their positive assessment. We hypothesized that palmitoylated synaptic proteins traffic correctly given our observation that they are appropriately localized in the expected subcellular fractions (Fig. 3D). However, we don't rule out some degree of mislocalization of PPT1 substrates, given evidence that the canonical PPT1 substrate V0AD1 misroutes to the plasma membrane (Bagh et al., Nat Commun 8, 2016). We have begun to test the hypothesis that they function poorly for the GluA1 subunit of the AMPA receptor (Fig. 3E) and for synaptic adhesion molecules (Fig. 4E). Future studies will test if GluA1 trafficking, assembly and function are altered in PPT1 KO neuronal cultures upon acute deletions of PPT1. Similarly, we will test if SynCAM self-assembly and synaptogenesis are altered in heterologous cultures of PPT1 KO. Each of these is an independent future study that builds on this resource. Similarly, we hope that our data will inspire and suggest new exciting directions for other investigators.

g) The higher power inserts of synaptic morphology in Figure 4D appear to lack a scale bar. Please can these be added.

Thank you for pointing out this omission. The higher power image is a 50 µM ROI as described in the figure legend. The scale bars have been added for clarity and readability.
